# New method for the identification of arbuscular mycorrhizal fungi by proteomic-based biotyping of spores using MALDI-TOF-MS

**DOI:** 10.1038/s41598-017-14487-6

**Published:** 2017-10-30

**Authors:** Thomas Crossay, Cyril Antheaume, Dirk Redecker, Lucie Bon, Nicolas Chedri, Clément Richert, Linda Guentas, Yvon Cavaloc, Hamid Amir

**Affiliations:** 1 Institut des Sciences Exactes et Appliquées (EA 7484), Université de Nouvelle-Calédonie, BP R4, 98851 Nouméa Nouvelle-Calédonie, France; 2 Plate-forme d’Analyse Chimique Strasbourg-Illkirch. Université de Strasbourg, F-67400 Illkirch, France; 3 Agroécologie, AgroSup Dijon, CNRS, INRA, Univ. Bourgogne Franche-Comté, F-21000 Dijon, France; 4 Institut Pasteur, Bacteriology Research Unit, 98800 Nouméa Nouvelle-Calédonie, France; 5 Bruker Corporation, Nouvelle-Calédonie, France

## Abstract

Arbuscular mycorrhizal fungi (AMF, Glomeromycota) are mutualistic symbionts associated with majority of land plants. These fungi play an important role in plant growth, but their taxonomic identification remains a challenge for academic research, culture collections and inoculum producers who need to certify their products. Identification of these fungi was traditionally performed based on their spore morphology. DNA sequence data have successfully been used to study the evolutionary relationships of AMF, develop molecular identification tools and assess their diversity in the environment. However, these methods require considerable expertise and are not well-adapted for “routine” quality control of culture collections and inoculum production. Here, we show that Matrix-Assisted Laser Desorption Ionisation Time of Flight Mass Spectrometry proteomic-based biotyping is a highly efficient approach for AMF identification. Nineteen isolates belonging to fourteen species, seven genera and five families were clearly differentiated by MALDI biotyping at the species level, and intraspecific differentiation was achieved for the majority. AMF identification by MALDI biotyping could be highly useful, not only for research but also in agricultural and environmental applications. Fast, accurate and inexpensive molecular mass determination and the possibility of automation make MALDI-TOF-MS a real alternative to conventional morphological and molecular methods for AMF identification.

## Introduction

Agriculture will face significant challenges in the 21^st^ century, largely due to the need to increase global food supply under the declining availability of soil, land and water resources, the environmental impacts of chemical inputs and the declining resources of mineral phosphate fertilizers. The main challenge is to develop and promote food and livelihood systems that have greater environmental, economic and social resilience to risk. Developing a sustainable crop production will require a shift from industrial crops, which generally rely on mono-cropping, the intensive application of commercial fertilizers, and the heavy use of pesticides and other inputs that are damaging to the environment, communities, and farm workers^1^. As a means towards developing sustainable agriculture, soil organisms that are considered plant bio-fertilizer have been used for biotechnological applications to agronomy and the environment. Arbuscular mycorrhizal fungi (AMF) are among these organisms and have been successfully used in both fields^2^. Fungi of the phylum Glomeromycota are ubiquitous hypogeous microorganisms that live in symbiosis with 80% of the world’s vascular land plants^3,4^. Despite their common occurrence and evidence of their association with land plants since their appearance 460 million years ago^5,6^, our knowledge of the genetics and diversity of AMF remains limited. Plant AMF symbiosis can be critically important in the development of sustainable agriculture, remediation of polluted lands and ecological restoration of degraded sites, such as mines^7^. As obligate biotrophs, AMF depend on host-derived carbon to complete their life cycle, and hosts have been estimated to transfer up to 20% to their photosynthetically fixed carbon to fungi^8^. In return for this substantial carbon cost, host plants obtain multiple benefits from their fungal partners: on average, 90% of the phosphorus and 60% of the nitrogen present in plants are sustained by these fungi^3^. The mycelial network of AMF extends into the soil volume and greatly increases the surface area for the uptake of immobile nutrients. AM symbioses also improve plant tolerance to drought and enhance their resistance to plant pathogens and their tolerance to heavy metals in polluted and metalliferous soils^7^. As a result, AMF are important determinants of plant nutrition and ecosystem productivity^9^.

The number of described AMF species is very low compared to other fungal phyla^10^. Currently, the Glomeromycota comprise approximately 270 known species^11^. Molecular diversity studies, however, have suggested the existence of 348 to 1,600 Glomeromycota species^10^. There are three possible reasons why unknown taxa have not been described to date. First, they may represent isolates that are difficult to grow^10^. Second, they may not have been discovered due to a lack of AMF sampling in many earth terrestrial regions. Third, only a small number of experts currently address morphological and molecular characterization of this group of fungi. Although various Glomeromycota species form different types of spores^12,13^, the type, mode of formation, subcellular structure, and characteristics of the spore components are frequently insufficient to identify fungi. Molecular characterization using DNA sequences was introduced in the 1990s^14^ to detect, identify and quantify AMF in the roots of plants. The main challenges in AMF research are the power of molecular markers used for quantification and identification of AMF at intra and inter-specific levels. Large intra-isolate variation of nuclear ribosomal genes^15,16^ complicates assignment of a single marker gene sequence to a fungal strain and assessment of the diversity of AMF in molecular field studies. The search for new marker genes has additionally been hampered by the great difficulty encountered in assembling the nuclear genome of *Rhizophagus irregularis* ^17^. An often-used fragment for resolving closely related species comprises the SSU rRNA gene, the whole internal transcribed spacer (ITS) rDNA region, including the 5.8 S rRNA gene, and the partial large subunit (LSU) rRNA gene, herein referred to as SSU–ITS–LSU^18^. As explained in Krüger *et al*.^19^, ITS1 and ITS2 regions are highly variable and must be excluded when different families are included in the same phylogenetic analysis; this is because alignment is impossible among higher taxa.

Morphological identification of AMF is time-consuming and requires considerable expertise, while DNA-based methods are still time-consuming and expensive.

A reliable and simple technique to discriminate AMF could help removing a roadblock on the way to large-scale AMF usage. A prerequisite for the application of AMF as biological fertilizer for agricultural and environmental uses is the ability to perform strict quality control of the inoculum. Quality control consists of identifying and quantifying which species of AMF are present in the inoculum and in determining the absence of pathogens. A quick, accurate taxonomic identification of AMF isolates is necessary for culture collections (IBG, INVAM, SAF, CICG, and GINCO) and research and for industrial certification.

An alternative approach to DNA-based isolate characterization, which to our knowledge has never been applied to AMF, is proteomic-based chemotaxonomic biotyping using MALDI-TOF mass spectrometry. This technique is already used in the fields of forensic and medical diagnosis. It allows rapid and accurate identification of microorganisms. The use of mass spectrometry to characterize microorganisms dates back to 1975^20^. Subsequently, methods were quickly optimized and used in clinical and environmental contexts. MALDI-TOF-MS biotyping has successfully been used to identify bacteria, yeast, fungi, and higher eukaryotes including insects, molluscs and fish^21–23^. In fact, MALDI-TOF-MS based identification of fungi provided more accurate results than morphology-based analyses^24^. Furthermore, this technology is less expensive, easier and faster than current DNA based-identification^25^. MALDI-TOF-MS and gene sequencing methods resulted in highly similar groupings of entomopathogenic fungus species of the genus *Metarhizium* ^26^, and similar results were observed for soil fungal species of the genus *Trichoderma* ^27^ and plant pathogenic fungi of the genus *Alternaria* ^28^. All these authors highlight the fact that identification by MALDI-TOF-MS is useful for culture collections because once the protein profiles of fungus isolates are obtained and included in a MALDI-TOF-MS library, matching is easily accomplished by simple comparison between stored profiles and a newly obtained profile. The limitation of the technology is that identification of new isolates is possible only if the spectral database contains protein mass fingerprints for the type strains of specific genera, species or subspecies^29^. However, the potential for this approach to be applied to green biotechnology has not been explored. In this study we rigorously evaluated the possibility to identifying AMF spores of nineteen isolates belonging to fourteen species, seven genera and five families by MALDI-TOF-MS biotyping.

## Results

### Phylogenetic analysis

**Figure 1:**
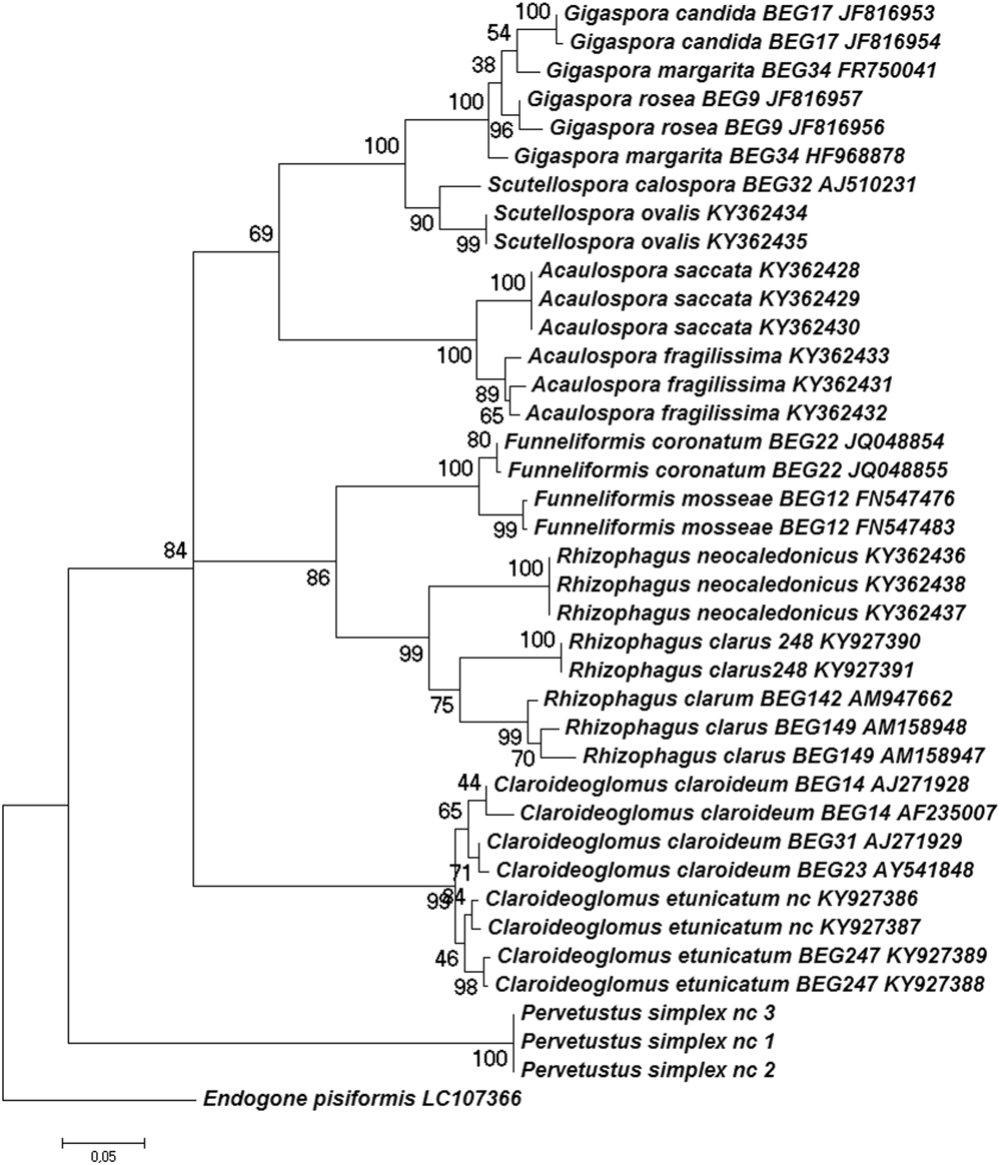
Phylogenetic tree of partial 18S-5.8S-partial 28 S rDNA sequences of 19 Glomeromycota isolates. Maximum likelihood (ML) analysis including *Endogone pisiformis* as outgroup. ML phylogenetic tree was based on individual partial small subunit–5.8S-partial large subunit (partial 18S-5.8S-partial 28 S) rDNA sequence. When partial 18S-5.8S-partial 28 S were not available, partial LSU sequences were used. Bootstrap values are given for each branch. Scale bar indicates the number of substitutions per site.

The partial 18S-5.8S-partial 28 S region of nuclear rRNA or the partial 28 S region of all isolates also analysed by MALDI was used for maximum likelihood phylogenetic analyses in MEGA and PhyML (Fig. 1). ITS1 and ITS2 regions were excluded from the alignment because they are highly variable. Phylogenetic analysis (Fig. 1) confirmed morphological identification, with the exception of two sequences of *G*. *margarita* species that were not recovered as a monophyletic clade.

### Analysis of intra- and inter-isolate spectrum variation of MALDI-TOF MS using the main spectrum (MSP) database

**Figure 2:**
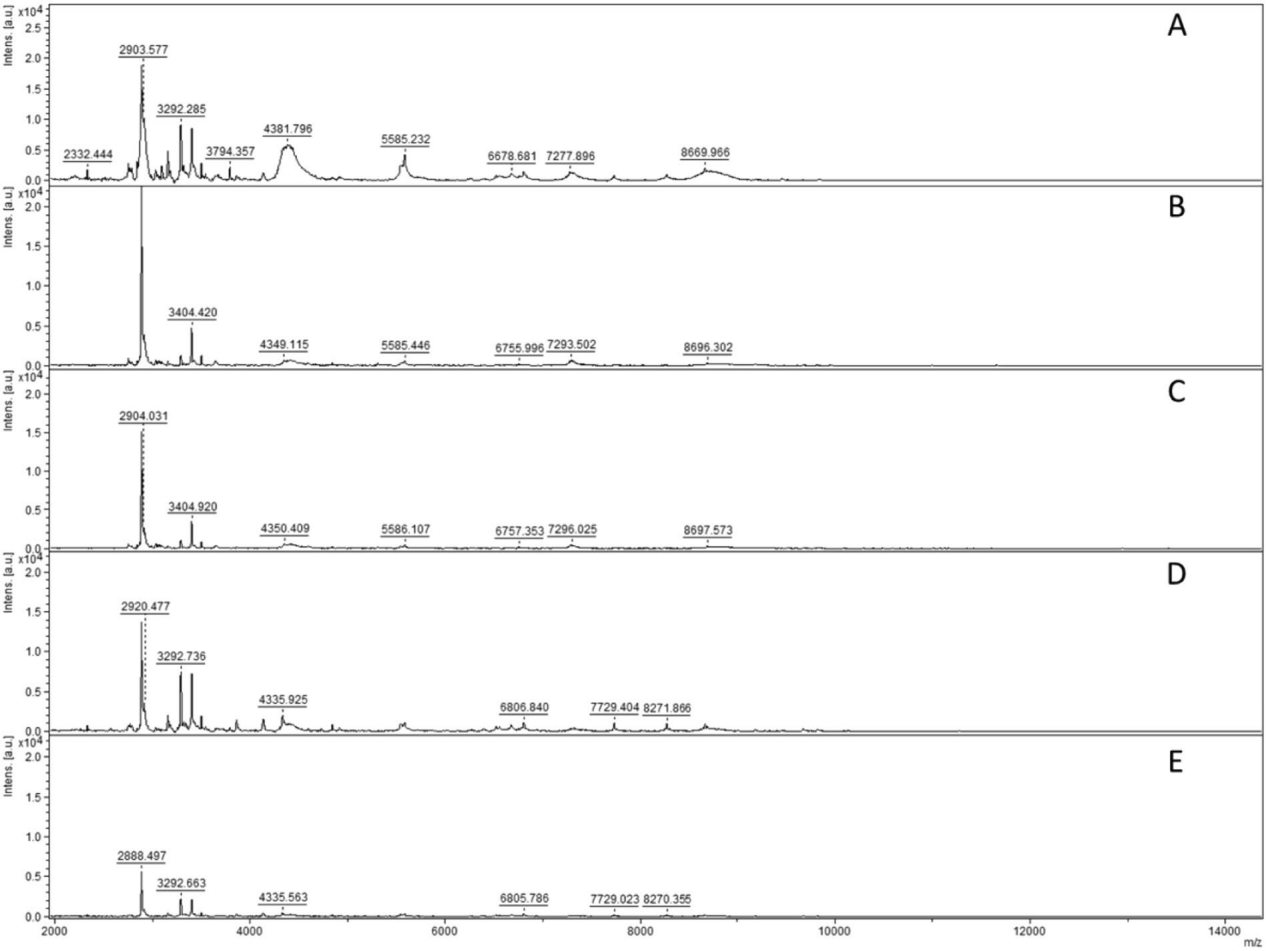
Limit of MALDI-TOF mass spectra detection using (**A**) 10, (**B**) 5, (**C**) 4, (**D**) 2 and (**E**) 1 crushed spores to extract *Scutellospora ovalis*.

We assessed the detection limit of MALDI-TOF-MS, by successively using 10, 5, 4, 2 and 1 spores of *Scutellospora ovalis*. A good signal to noise ratio was obtained with two spores of this species. For this isolate, we also verified spectral reproducibility for any number of spores (Fig. 2). Based on this result, we decided to normalize the amount of spore material per sample to a minimum volume equivalent to two spores of *Scutellospora ovalis* (estimated as 2270700 µm^3^) for all studied isolates. A single spore was used for species with a spore volume > 2270700 μm^3^. Origin, spore diameter, estimated volume of one spore and spore number used per sample of all isolates studied are presented in Supplementary Table 1.

**Figure 3:**
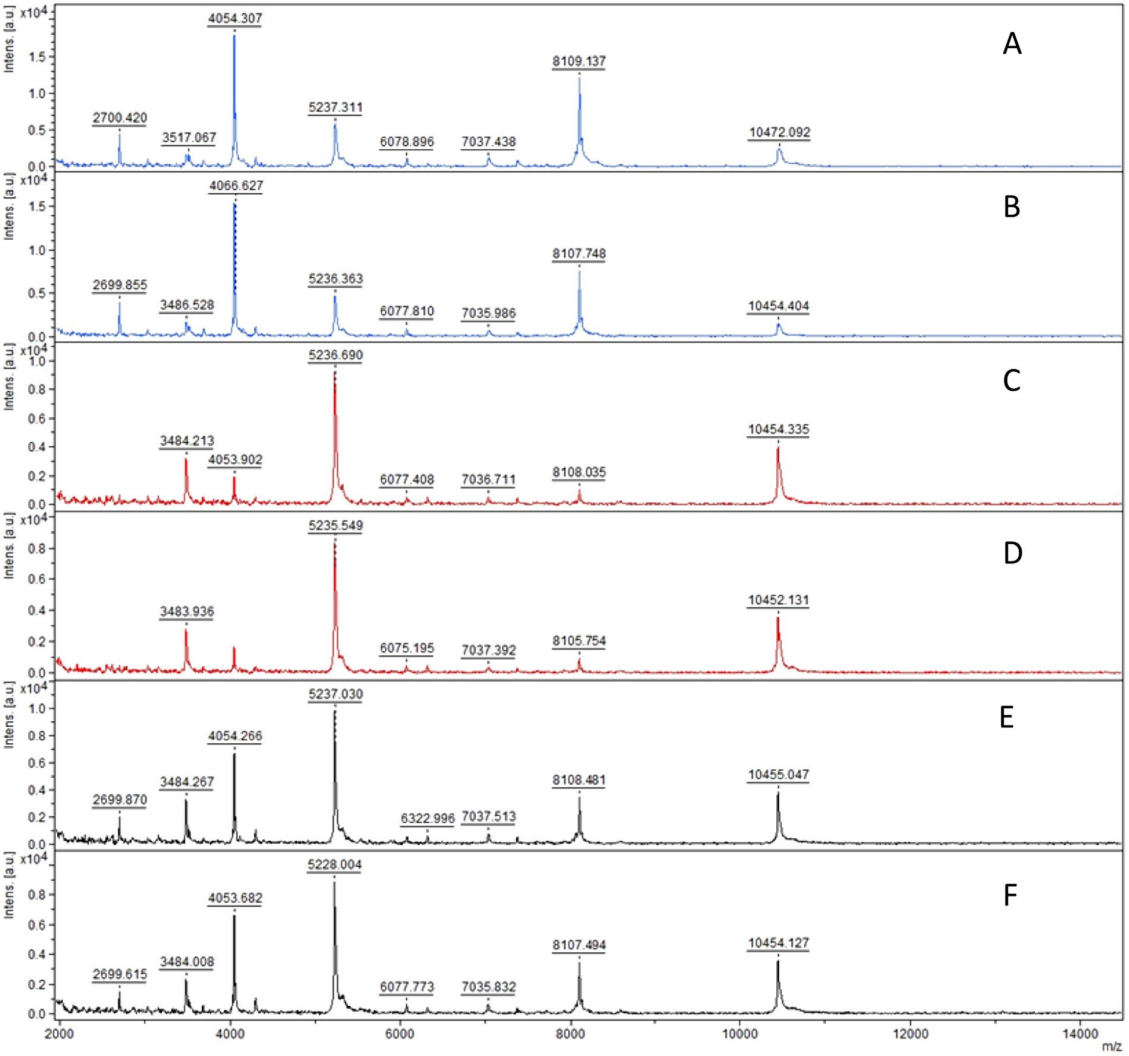
Comparison of mass spectra of two replicates each of mature and juvenile spores of *F*. *mosseae* BEG12. (**A**,**B**) Mature spores of *F*. *mosseae* BEG12; (**C**,**D**) juvenile spores of *F*. *mosseae* BEG12; (**E**,**F**) mix of juvenile and mature spores of *F*. *mosseae* BEG12.

For some species, spectra were compared between young and mature spores. Spores of *F*. *mosseae* BEG12 usually double in size from young to mature and are thus categorized based on spore diameter (Supplementary Fig. 1). They showed differences only in the intensity of four major peaks, rather than in their m/z ratio (ratio mass/charge number of ions) (m/z: 4054 ± 1, 5228 ± 1, 8109 ± 2, 10466 ± 9), and in the presence of one minor peak at m/z 2700 ± 1, which was not found in the juvenile stage. Despite this slight variation between mature, juvenile and mixed sample spectra, the software gave a sufficient score to match all of these to the same isolate (Fig. 3).

**Figure 4:**
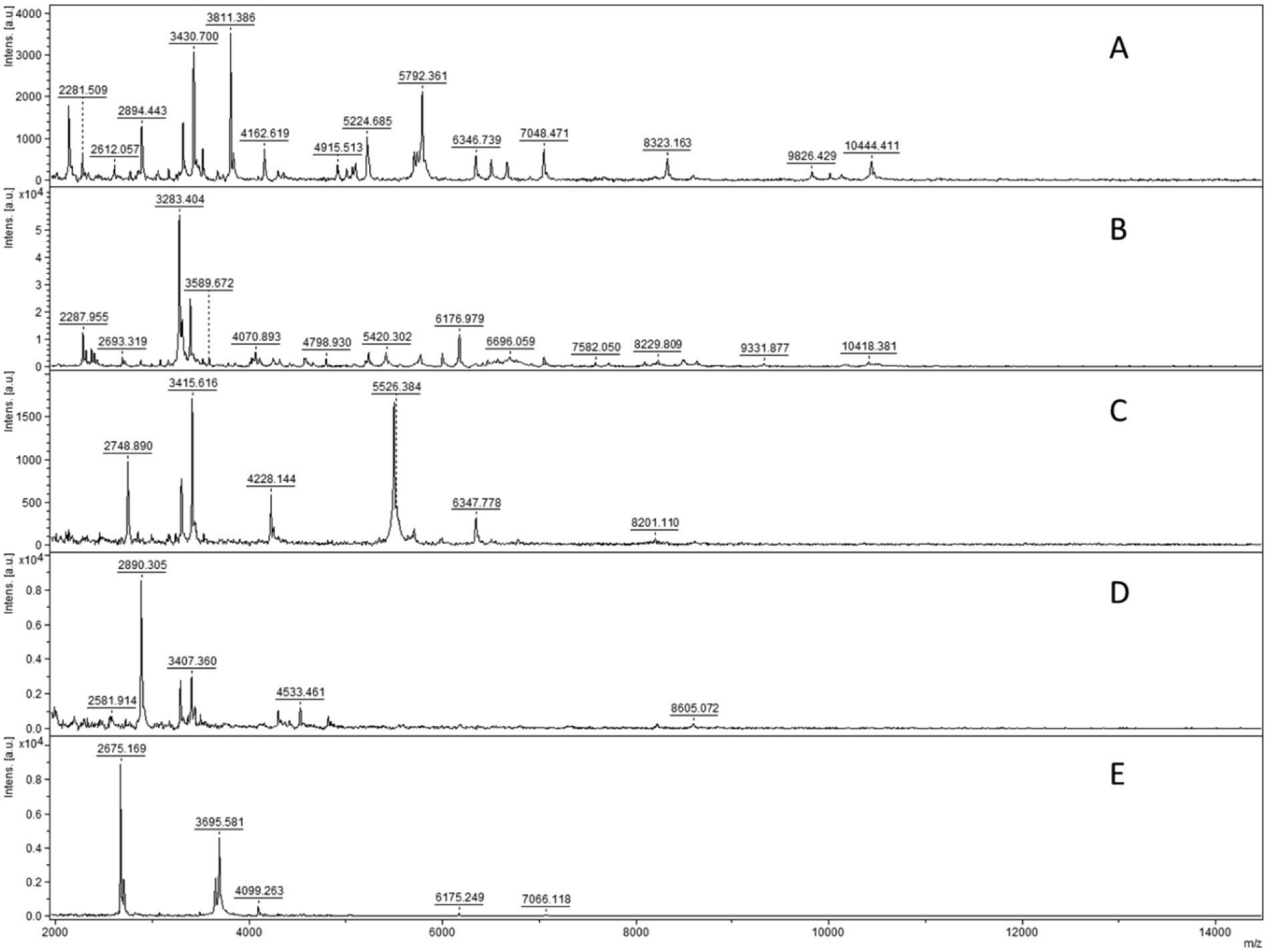
Comparison of MALDI-TOF mass spectra of: (**A**) *Gigaspora rosea* BEG9; (**B**) *Gigaspora margarita* BEG34; (**C**) *Gigaspora candida* BEG17; (**D**) *Scutellospora ovalis* *;* and (**E**) *Scutellospora calospora* BEG32.

Representative mass spectra of five species of Gigasporaceae are illustrated in Fig. 4. A single spore per analysis was used, except for *Scutellospora* species: for *S*. *ovalis*, two spores were used and for *S*. *calospora* BEG32, three spores were used per analysis (Supplementary Table 1). The Gigasporaceae species yielded clearly different spectra (Fig. 4) and can be easily differentiated, as all other studied species (Supplementary Fig. 2).

**Figure 5:**
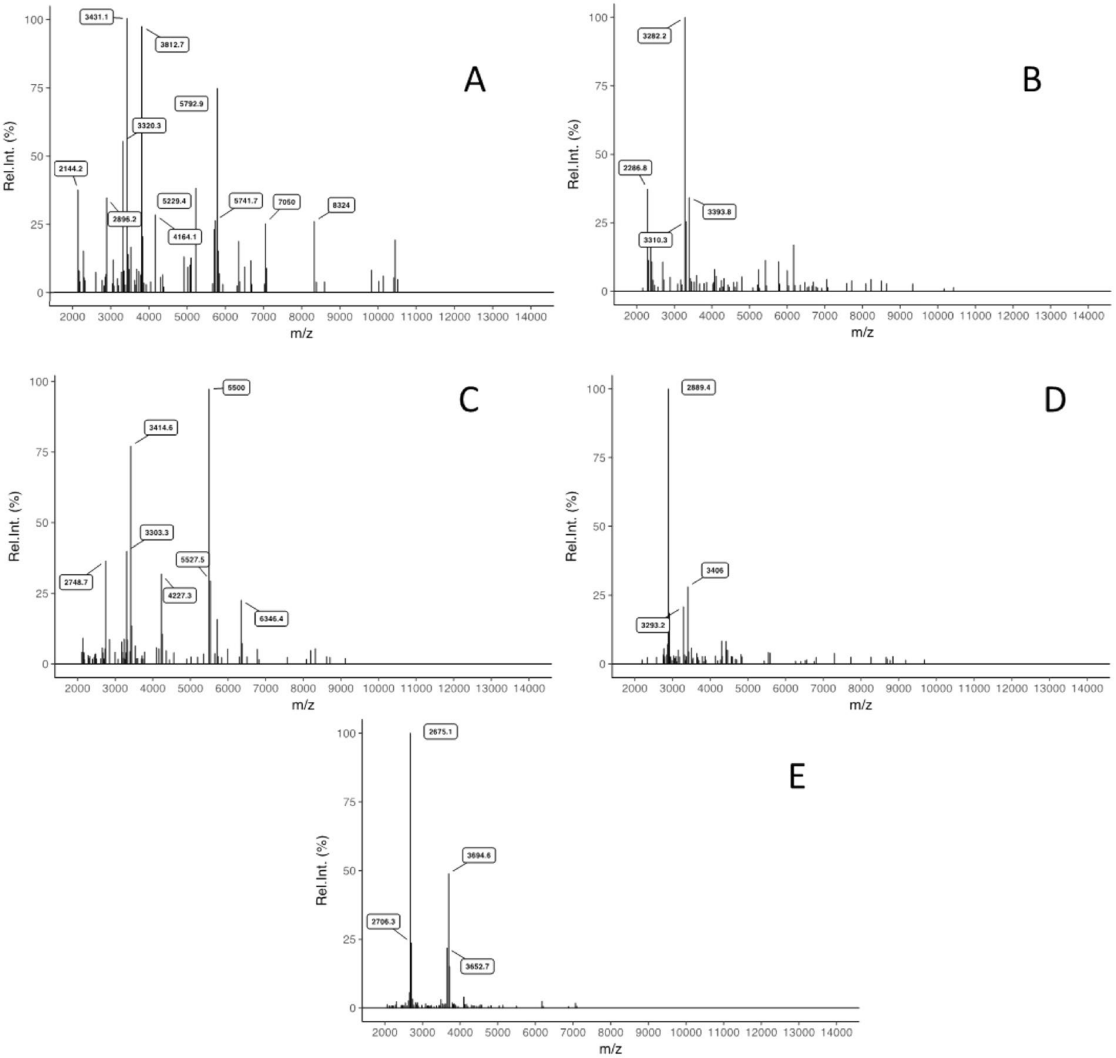
Main Spectrum Patterns of: (**A**) *Gigaspora rosea* BEG9; (**B**) *Gigaspora margarita* BEG34; (**C**) *Gigaspora candida* BEG17; (**D**) *Scutellospora ovalis*; and (**E**) *Scutellospora calospora* BEG32. Peaks were labelled if they had a minimum of 20% relative intensity.

The analyses yielded spectra of sufficient quality to generate MSPs (Main Spectrum Patterns; see Material and Methods, section data processing for MSP creation and MALDI-TOF-MS data export for graphical MSP creation in R software^30^). MSPs of microbial isolates are commonly used as reference spectra for identification by comparison to unknown samples. A graphical MSP representation of the Gigasporaceae species is shown in Fig. 5. All other spectra are provided as supplementary material (Supplementary Fig. 3). MSPs of different AMF species markedly differ, demonstrating that biotyping by MALDI-TOF-MS can easily distinguish the AMF species in question. For each fungal species, a single peak could be identified as a marker of the respective species. No single peak was common to all species tested.

**Figure 6:**
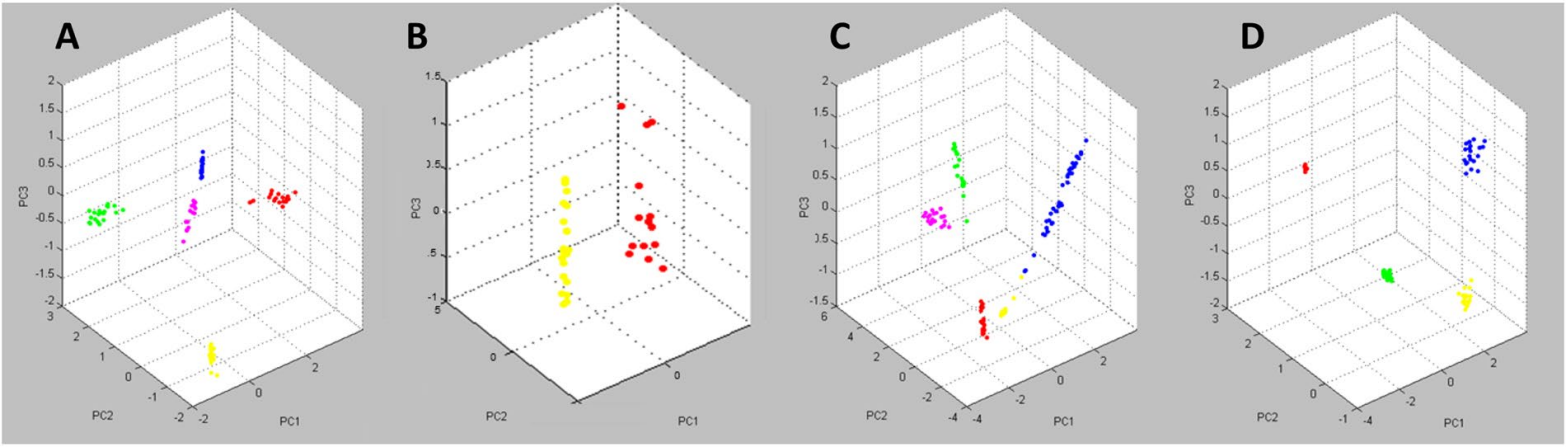
Three-dimensional principal component analysis (PCA) plot of 20 available spectra (replicates) of different species isolates of: (**A**) Gigasporaceae: *G*. *rosea* BEG9 in red; *G*. *margarita* BEG34 in yellow; *G*. *candida* BEG17 in green; *S*. *ovalis* in pink; and *S*. *calospora* BEG32 in blue. (**B**) Acaulosporaceae: *A*. *saccata* in yellow and *A*. *fragilissima* in red. (**C**) Glomeraceae: *R*. *neocaledonicus* in pink; *R*. *clarus* BEG248 and *R*. *clarus* BEG149 in blue; *R*. *clarus* BEG142 in yellow; *F*. *coronatum* BEG22 in red; *F*. *mosseae* in green. (**D**) Claroideoglomeraceae: *C*. *etunicatum* BEG247 in yellow; *C*. *etunicatum nc* in blue; *C*. *claroideum* BEG31 in red; *C*. *claroideum* BEG23 and *C*. *claroideum* BEG14 in green.

For each family but the Pervetustaceae (only one species), a Principal Component Analysis (PCA) was performed to investigate the intra- and inter-species variation of the spectra. With a few exceptions, technical and biological replicates corresponding to one studied isolate formed well-separated clusters (Fig. 6). The exceptions were *R*. *clarus* BEG248/149, which were not separated from each other, and *C*. *claroideum* BEG23/14. In all families, species were clearly differentiated, and in several cases, the isolates of the same species were be differentiated.

**Figure 7:**
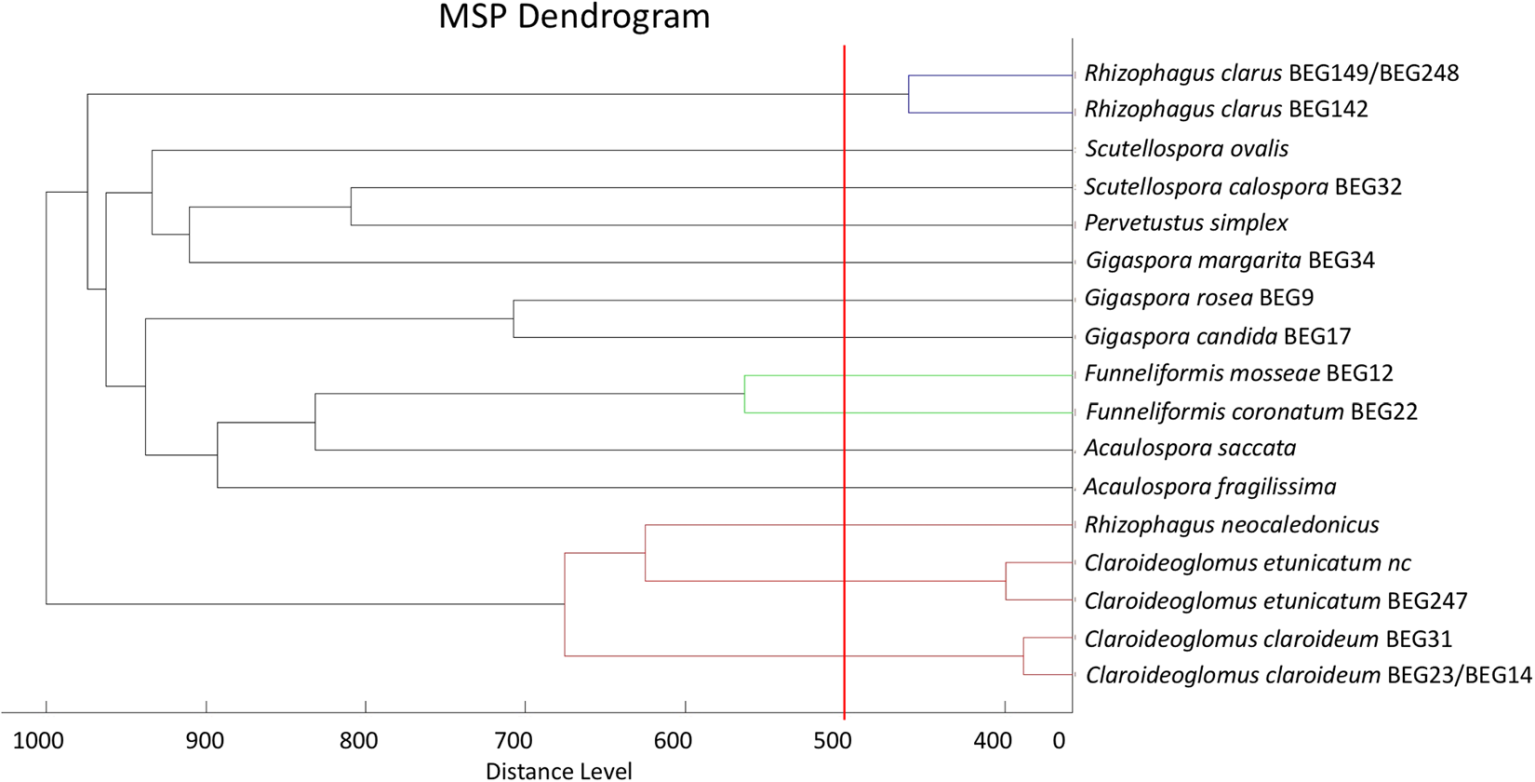
Cluster analysis of MALDI-TOF-MS of selected MSP references. Distance is displayed in relative units. Distance levels below 500 (represented by the red line) were considered reliably classified to the same species^31^.

A dendrogram created with the reference spectra allows easy comparison of the isolates (Fig. 7). All studied species were well identified at a distance level of more than 500 units^31^ (see Materials and Methods), but isolates of the same species were not always differentiated. *Claroideoglomus claroideum* BEG23 and *Claroideoglomus claroideum* BEG14 could not be discriminated by distance values calculated between MSP references of these two isolates, nor were *Rhizophagus clarus* BEG149 and *Rhizophagus clarus* BEG248 differentiated. Other isolates of the same species were differentiated in the dendrogram: *R*. *clarus* BEG248/149 versus *R*. *clarus* BEG142; *C*. *etunicatum* BEG247 versus *C*. *etunicatum* nc and *C*. *claroideum* BEG23/14 versus *C*. *claroideum* BEG31.

### MALDI-TOF-MS database matching real-time blind test

Using our homemade database, we also used the Bruker “*real time identification*” software, which is ordinarily used for clinical diagnosis, to evaluate the robustness of AMF identification from the perspective of strain certification. The identification rate of Gigasporaceae species was 100%, with Main Pattern scores (MP scores, see Material and methods) higher than 1.920 (Table 1). The identification rate of species in the Acaulosporaceae was also 100%, with MP scores ranging from 1.500 to 2.590. The species identification rate of Glomeraceae was 100%, with MP scores ranging from 1.590 to 2.550. *R*. *clarus* BEG248 and *R*. *clarus* BEG149 have very similar spectra, as explained above; therefore, the method identified them with virtually equivalent scores and provided them as identical candidates or species. *R*. *clarus* isolate BEG142 was clearly identified, with no matching second candidate. The identification rate of species in the Claroideoglomeraceae was 100%, with MP scores ranging from 1.720 to 2.260. *C*. *claroideum* BEG23 and *C*. *claroideum* BEG14 have very similar spectra, as explained above. For *C*. *claroideum* BEG23, one of the three samples was identified as *C*. *claroideum* BEG14, with an MP score of 2.10, but *C*. *claroideum* BEG14 was presented as the second candidate, with an MP score of 1.920. *C*. *claroideum* isolate BEG31 was distinguished from the others, with no second candidate given by the software. The two isolates of *C*. *etunicatum* BEG247 and *C*. *etunicatum nc* were also identified correctly. In the Pervetustaceae, a single isolate has been studied: *Pervetustus simplex nc*. Two of the three samples allowed identification of this isolate, with MP scores of 1.760 and 1.370, whereas the third sample did not produce a signal.

**Table 1:** Spore identification of 19 isolates of AMF by MALDI-TOF-MS biotyper: Bruker real-time analysis of log MP scores.

Family	Genus	Species/Isolates	Nb spores	MP scores value	Organism (Best match/second best match > 1.500)
Gigasporaceae	*Gigaspora*	*G*. *rosea* BEG9	1	2.370	*G*. *rosea* BEG9
			1	2.290	*G*. *rosea* BEG9
			1	2.470	*G*. *rosea* BEG9
		*G*. *margarita* BEG34	1	2.250	*G*. *margarita* BEG34
			1	2.140	*G*. *margarita* BEG34
			1	2.120	*G*. *margarita* BEG34
		*G*. *candida* BEG17	1	2.300	*G*. *candida* BEG17
			1	2.420	*G*. *candida* BEG17
			1	2.250	*G*. *candida* BEG17
	*Scutellospora*	*S*. *ovalis*	2	1.920	*S*. *ovalis*
			2	2.130	*S*. *ovalis*
			2	2.600	*S*. *ovalis*
		*S*. *calospora* BEG32	3	2.270	*S*. *calospora* BEG32
			3	2.310	*S*. *calospora* BEG32
			3	2.220	*S*. *calospora* BEG32
Acaulosporaceae	*Acaulospora*	*A*. *fragilissima*	14	2.590	*A*. *fragilissima*
			14	2.190	*A*. *fragilissima*
			14	2.170	*A*. *fragilissima*
		*A*. *saccata*	10	1.870	*A*. *saccata*
			10	1.830	*A*. *saccata*
			10	1.500	*A*. *saccata*
Glomeraceae	*Rhizophagus*	*R*. *neocaledonicus*	10	1.780	*R*. *neocaledonicus*
			10	1.660	*R*. *neocaledonicus*
			10	1.670	*R*. *neocaledonicus*
		*R*. *clarus* BEG248	1	2.530/**2.300**	*R*. *clarus* BEG248/**BEG149**
			1	2.490/**2.260**	*R*. *clarus* BEG248/**BEG149**
			1	2.550/**2.370**	*R*. *clarus* BEG248/**BEG149**
		*R*. *clarus* BEG149	1	2.440/**2.350**	*R*. *clarus* BEG149/**BEG248**
			1	2.330/**2.270**	*R*. *clarus* BEG248/**BEG149**
			1	2.410/**2.340**	*R*. *clarus* BEG149/**BEG248**
		*R*. *clarus* BEG142	1	1.590	*R*. *clarus* BEG142
			1	1.820	*R*. *clarus* BEG142
			1	1.900	*R*. *clarus* BEG142
	*Funneliformis*	*F*. *coronatum* BEG 22	1	1.940	*F*. *coronatum* BEG22
			1	2.130	*F*. *coronatum* BEG22
			1	1.840	*F*. *coronatum* BEG22
		*F*. *mosseae* BEG12	4	2.050	*F*. *mosseae* BEG12
			4	1.900	*F*. *mosseae* BEG12
			4	2.080	*F*. *mosseae* BEG12
Claroideoglomeraceae	*Claroideoglomus*	*C*. *etunicatum* BEG247	7	2.130	*C*. *etunicatum* BEG247
			7	1.720	*C*. *etunicatum* BEG247
			7	2.000	C. *etunicatum* BEG247
		*C*. *etunicatum nc*	6	2.140	*C*. *etunicatum nc*
			6	2.220	*C*. *etunicatum nc*
			6	2.010	*C*. *etunicatum nc*
		*C*. *claroideum* BEG31	4	2.070	*C*. *claroideum* BEG31
			4	2.040	*C*. *claroideum* BEG31
			4	2.090	*C*. *claroideum* BEG31
		*C*. *claroideum* BEG14	4	2.140/**1.760**	*C*. *claroideum* BEG14/**BEG23**
			4	2.220/**1.350**	*C*. *claroideum* BEG14/**BEG23**
			4	2.260/**1.820**	*C*. *claroideum* BEG14/**BEG23**
		*C*. *claroideum* BEG23	4	2.10/**1.920**	*C*. *claroideum* BEG14/**BEG23**
			4	2.170/**2.040**	*C*. *claroideum* BEG23/**BEG14**
			4	1.870/**1.690**	*C*. *claroideum* BEG23/**BEG14**
Pervetustaceae	*Pervetustus*	*P*. *simplex nc*	29	1.760	*P*. *simplex nc*
			29	1.370	*P*. *simplex nc*
			29	0	—

Bold scores indicate that the method identifies a second candidate; the second candidate is presented (in bold) only for MP scores greater than 1.500.

In summary, AMF species were reliably identified using this method: no misidentification was observed at the species level. Various isolates of the same species were correctly assigned in 22 out of 24 cases, even for *R*. *clarus* BEG248/BEG149 and *C*. *claroideum* BEG14/BEG23 who have very similar spectra. Identification at isolate level could be correctly assigned for these last four isolates in 10 out of 12 cases. Even when scores <1.7 were obtained, species identification matched the reference methods. One sample of *Pervetustus simplex nc* gave no signal and another from the same isolate gave a score under 1.5 (1.370).

### MALDI-TOF-MS data export and identification by graphical analyses

**Figure 8:**
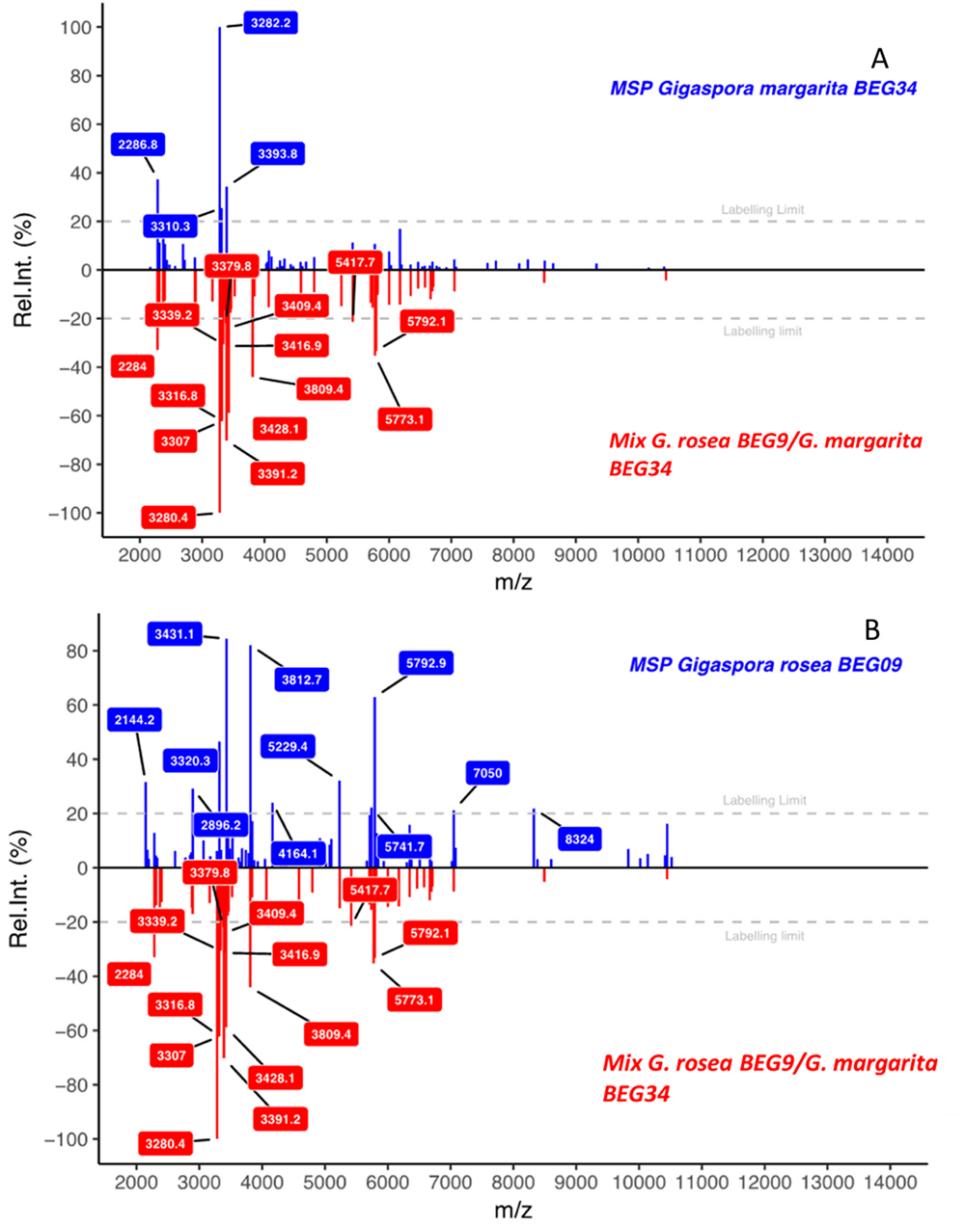
Example using R (version 3.3.2^30^) MSP importation data based on a mixed-species sample. The unknown sample (red) is matched to MSP reference spectra (blue). (**A**) *G*. *margarita* MSP in blue versus mixed-species sample (*G*. *rosea* BEG9/*G*. *margarita* BEG34). (**B**) *G*. *rosea* BEG9 MSP in blue versus mixed-species sample (*G*. *rosea* BEG9/*G*. *margarita* BEG34).

The spectra of a mixed sample *Gigaspora rosea* BEG9/*Gigaspora margarita* BEG34, using one spore of each species, were compared to all MSP peak lists. Comparison of the MSP spectrum of each of the two species with the spectrum of their mixture is presented in Fig. 8. A comparison of the spectrum of the mixed sample with all MSPs is available in supplementary Fig. 4. Among the m/z of the six most intense peaks of the mixed sample, three peaks belong to the species *G*. *margarita* (2284 ± 3, 3282 ± 2, 3393 ± 2) and three belong to the species *G*. *rosea* (3319 ± 2, 3430 ± 2, 3812 ± 3). This graphical comparison showed that cross-contamination with other Glomeromycota can be easily detected using our approach.

### Influences of culture substrate, storage, and subculture on spore MALDI-TOF-MS spectra

**Figure 9:**
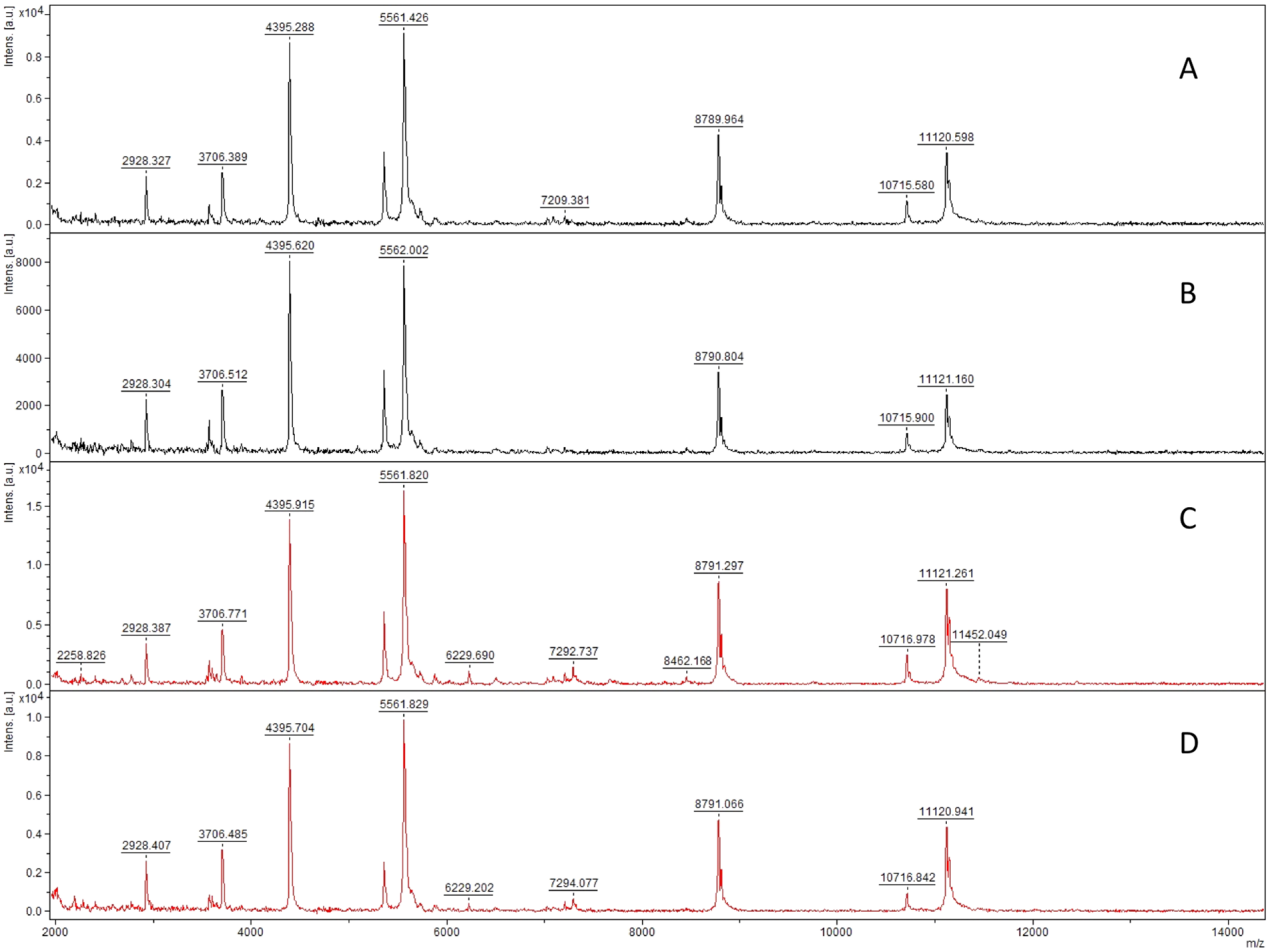
Replicate (n = 2) MALDI -TOF mass spectra of six spores of *C*. *etunicatum* BEG247 of (**A**,**B**) a culture from 2011 and (**C**,**D**) a culture from 2014. Subculture did not affect the score.

Using Bruker “*real time identification*” software, we studied the robustness of the method by evaluating different aspects of AMF culture and its potential constraints. Two subcultures of *C*. *etunicatum* BEG247 that differed in age by three years (from 2011 and 2014) demonstrated strictly the same spectral pattern (Fig. 9). The BEG247 MSP was obtained using the 2014 subculture, and the other samples scored up to 2 with the MSP reference. Therefore, the spectral patterns obtained using the MALDI-TOF-MS biotyping approach are independent of culture age.

**Figure 10:**
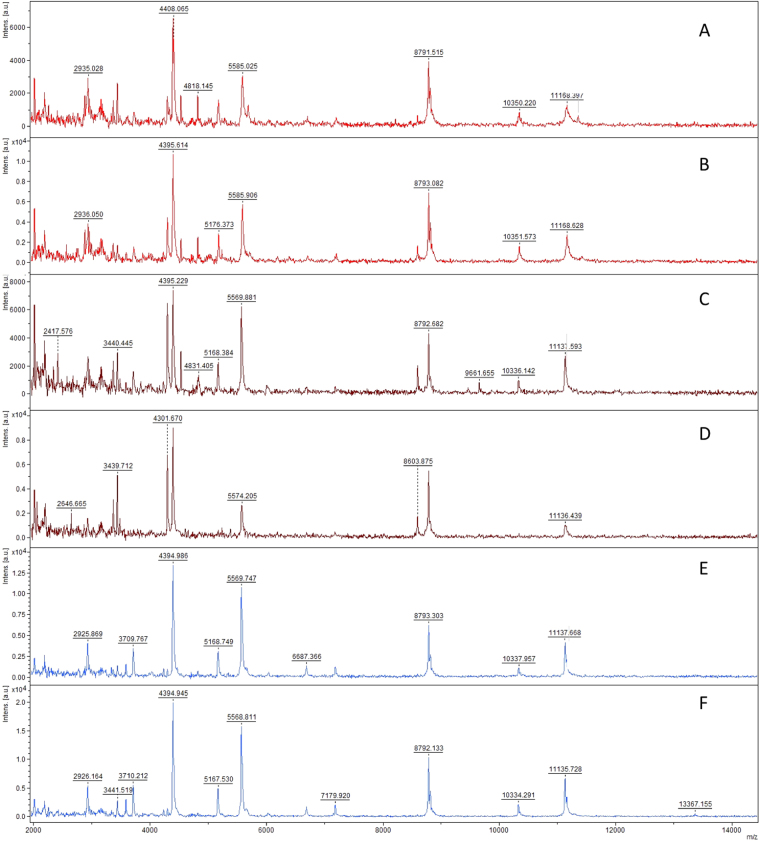
Replicate (n = 2) MALDI-TOF mass spectra of six spores of *Claroideoglomus etunicatum nc* (**A**,**B**) grown on ultramafic soil and (**C**,**D**) commercial compost, all collected on humid soil of pure and fresh “living culture” with (**E**,**F**) two spectra used for MSP reference obtained after three-month storage of dry ultramafic soil.

Storage and substrate were found to have a slight impact on the spectrum profile for *Claroideoglomus etunicatum nc*, but the observed variation of the spectra did not affect the accuracy of identification by MALDI-TOF-MS, with log scores between 1.86 for compost and 2.12 for wet ultramafic soil in comparison with MSP ultramafic dry reference soil (Fig. 10). All of these profiles (Fig. 10A,B) and (Fig. 10C,D) compared to real MSP references in our internal database allowed identification at the isolate level with *Claroideoglomus etunicatum nc*. These experiments revealed that neither culture substrate (ultramafic soil or commercial compost), spore storage in dry or wet soils, nor subculture affected the accuracy of MALDI-TOF-MS identifications.

## Discussion

In this study, we use MALDI-TOF-MS biotyping as a novel technique for AMF isolate characterization. This approach has been used for a variety of organisms^23,32–35^, among them human pathogenic fungi^36–39^, but is still an “underutilized technique, especially from a biotechnology perspective”^32^. We developed novel bioinformatics tools to facilitate database matching, which is essential for future usability.

The strength of our method clearly lies in resolving closely related taxa. As evident from the dendrogram (Fig. 7), large-scale phylogenetic relationships (i.e., among genera and beyond) were not reliably recovered, as shown in the rDNA phylogeny (Fig. 1) and as can be expected from a protein profile. We showed that the obtained proteome profiles are highly reproducible; we also clearly distinguished closely related species such as *C*. *claroideum/etunicatum* or *F*. *mosseae/coronatum*, or even *Gigaspora* species, some of which are genetically quite close and notoriously difficult to separate using nuclear ribosomal genes^40,41^, mitochondrially-encoded COI gene^42^, or the largest subunit of RNA polymerase II (RPB1) gene^43^.

Furthermore, MALDI-TOF biotyping proved far superior to currently used DNA based analysis in distinguishing isolates of the species *C*. *etunicatum* and two out of three isolates in *R*. *clarus* and *C*. *claroideum*. The possibility of identifying intra-species isolates by MALDI-TOF-MS has been demonstrated for several clinically relevant bacteria, such as methicillin-resistant *Staphylococcus aureus* strains^44^, *Haemophilus influenzae* Type b isolates^45^ or *Clostridium difficile* ^46^. While this appears to be well established for bacteria, sub-species typing of fungi by MALDI-TOF-MS must still be properly assessed^36^.

In Fig. 1, we present two sequences per isolate, which all grouped together with the exception of *G*. *margarita*. The failure to recover the two sequences, which are separated by weakly supported nodes, as a monophyletic group is consistent with previous studies which indicated the difficulty to assess *Gigaspora* diversity due to low inter-species variation in some regions of the rRNA operon and high variation even within spores in others^47,48^. The clear separation of these taxa by MALDI spectra reinforces that the new method will be highly useful to distinguish closely-related fungi.

However, not all isolates of the same species could be distinguished, suggesting that they were genetically too similar. Interestingly, ribosomal sequences and biotyping provided discordant results in terms of the similarity among the three isolates studied here: BEG142 and BEG149 were very close in the rRNA sequencing approach whereas BEG149 and BEG248 were closely related in proteomic analysis. Indeed, BEG248 (formerly known as LPA64) grouped at a certain distance from the other two isolates in the rDNA phylogenetic tree, which we confirmed by resequencing its LSU after an initial analysis in 2002 (accession number AJ510243). These findings will be the starting point for further analyses of the species *R*. *clarus* to clarify its morphological and molecular diversity.

Several studies have found no significant influence of culture media and culture age on bacterial and fungal protein mass spectral patterns. Apparently, dominant peaks result from ribosomal and house-keeping proteins, which generally remain mainly unchanged under different growth conditions and throughout different growth stages^35–39^. Using *Funneliformis mosseae* BEG12, we explored whether culture age altered AMF mass spectra. The spectral pattern did not change between juvenile and mature spores, except for one minor peak at m/z 2700 ± 1 not found at a juvenile stage and some peak intensities. The latter likely results from changes in the ratio of the quantity of proteins between the two stages. This result is in accordance with the study of Emami *et al*.^32^ on microalgae. In addition, we confirmed that the duration of storage and subculture did not affect the accuracy of MALDI-TOF-MS. As AMF are normally cultivated with their host plants under non-sterile conditions and their spores are known to contain numerous other microorganisms^49,50^, the stability of the patterns (regardless of the culture environment) is of particular concern for AMF strain typing.

These results indicate that this technique is clearly suitable for AMF identification and could be a reliable alternative to DNA sequencing. The real value of the approach lies in its ability to differentiate between closely related isolates and its better cost efficiency and lower turnover time compared to current DNA sequencing approaches. Preparation of a biological sample for a single identification of one isolate requires less than one hour of work. The identification of this sample by MALDI-TOF-MS biotyping requires at most a few minutes. In addition, MALDI-TOF-MS is much more affordable than conventional DNA sequencing, despite the cost of instrument and regardless of the microorganism^25,51–53^ (Table 2).

**Table 2:** Time and estimated cost analysis for rDNA sequencing from one isolate using conventional Sanger sequencing versus MALDI-TOF-MS biotyping.

DNA sequencing (Sanger)		MALDI-TOF-MS	
Hands-on time	Hands-off time	Hands-on time	Hands-off time
Process/work time			
DNA extraction		Protein extraction	
20 min	0 min		
Nested PCR steps		1 min	5 min
40 min	5 hr		
Cloning steps		MALDI sampling	
3–4 hr	1–2 days		
Sequencing reaction		10 min	5 min
25 min	4 hr		
Sequencing clean up		Data acquisition	
30 min	0 hr		
Sanger Sequencing		5 min	1 min
5 min	1 hr		
Total time			
Total time/sample		Total time/sample	
5–6 hr	34–58 hr	16 min	11 min
Approximate cost			
(Chemistry and consumables)			
Total Price/sample		Total Price/sample	
17 US $ with cloning		3 US $	
9.5 US $ without			
Approximate cost			
(Instruments)			
95000 US $		155000 US $	

The MALDI-TOF-MS approach could fulfil the current need for simple but efficient inoculum quality control and the isolate certification required by inoculum producers and research laboratories. Most AMF inocula are produced on host plants in open pot cultures, which are very prone to contamination and require rigorous quality control of the inoculum. Markers from the mitochondrial DNA have been used for quality checks for mass production of the AMF model organism *R*. *irregularis* ^54^. However, the transposition of this approach to track another isolate can be time consuming, at least for the design of specific qPCR primers and experimental procedures. The proteomic approach, developed in this study may be a useful alternative which can be easily used for identifying and detecting cross-contamination of AMF cultures in a broad range of AMF.

Whatever the micro-organism group, a well-populated database is essential for MALDI-TOF-MS real time identification. Reference isolates from the IBG culture collection were crucial for building this first version of the database. It must now be supplemented using additional isolates by various institutions who work with AMF, particularly AMF culture collections, to make this identification tool usable and effective for research and industry in a broad range of working environments. One remaining limitation is that AMF spores are required and that non-sporulating AMF thus could not be detected. In all “human pathogenic fungi” biotyping studies^36^, the mycelia of fungi were targeted for identification by MALDI-TOF-MS and therefore it is also important to further test this method with *in vitro* AMF extraradical mycelium. This limitation may be less of a concern in culture collections and inoculum production, as the fungi targeted there are usually good sporulators and the culture conditions are optimized to assure this. However, our approach will also be useful for identifying spores in the environment. This may allow to determine the presence or absence of spores of known isolates in the field for instance after inoculation. In the long term, it will nevertheless be interesting to identify m/z peaks characteristic for target AMF that can be detected even against the background of plant protein in root extracts^55^.

In conclusion, identification of AMF by MALDI-TOF-MS biotyping could have considerable value, not only for research but also in agricultural and environmental applications. Fast and accurate molecular mass determination and the possibility of automation makes MALDI-TOF-MS a real alternative to conventional morphological and molecular methods for identification of AMF.

## Material and Methods

### Arbuscular mycorrhizal fungi isolates and culture conditions

Nineteen AMF isolates (Supplementary Fig. 1; Table 1) were studied: 13 were obtained from the culture collection of International Bank for the Glomeromycoa (IBG, Dijon, France) and six were obtained by the *Laboratoire Insulaire du Vivant et de l’Environnement* (LIVE, New-Caledonia). Four of them are currently described as new species and are therefore marked here as *nomina inedita*: *Acaulospora saccata* D. Redecker, Crossay & Cilia. *nom*. *ined*., *Acaulospora fragilissima* D. Redecker, Crossay & Cilia. *nom*. *ined*., *Scutellospora ovalis* D. Redecker, Crossay & Cilia. *nom*. *ined*. and *Rhizophagus neocaledonicus* D. Redecker, Crossay & Cilia. *nom*. *ined*. *P*. *simplex nc* was described as a new species^56^. All of the latter isolates and *C*. *etunicatum nc* were isolated in New Caledonia. For the six New Caledonian isolates, the culture was initiated as follows: The cone-tainer technique^57^ was applied for producing single species cultures, and 100 surface-sterilized spores of each isolate were used. Pots were filled with 40 g of an autoclaved substrate consisting of ultramafic soil from the area where each fungus was isolated. As plant growth in this pure ultramafic soil was very slow, a mixture of 80% ultramafic soil and 20% commercial compost (v/v) was used. For *Claroideoglomus etunicatum nc* an additional experiment was conducted: 100 spores were inoculated as described previously in 100% commercial compost to evaluate the possible influence of the culture substrate on MALDI spectra. The soil was autoclaved three times at 120 °C for 1 h, with an interval of 24 h. The substrate was placed in 50 mL cone-tainers. Two-week-old AMF-free plantlets of *Sorghum vulgare* were inoculated with AMF and placed in each cone-tainer. Spores in 200 μL water were deposited in the vicinity of the intertwined roots, to contact roots across a maximum range of root physiological states. After two months, cone-tainers were transplanted into 450 mL pots filled with the same substrate. Single-species cultures were maintained in a greenhouse (temperature, 21–24 °C; relative humidity, 70%) for 6 months and irrigated manually every two days. To prevent cross-contamination, pots of isolates were grown separately for each isolate on aluminium benches. Benches were separated by two metres of space. Isolates obtained from IBG had been cultivated in custom-built growth chambers at 20–26 °C under a 16:8 h day-night rhythm using various host plants and substrates as detailed in the following: BEG9, BEG34 (leek/onion; 75% Epoisses soil pH 7.5, 25% perlite), BEG12, BEG14, BEG17, BEG22, BEG23 (leek/onion; Epoisses soil pH 7.5), BEG31, BEG32 (clover; 75% Marlins soil pH 5, 25% gravel); and BEG142, BEG149, BEG247, BEG248 (*Tephrosia* sp.; 75% Marlins soil pH 5, 25% perlite). Further details about these cultures are available at http://i-beg.eu.

### Arbuscular mycorrhizal fungi spore extraction

After six months of culture, spores were stored in dry soil at room temperature for three months and extracted by wet sieving and sucrose density gradient centrifugation, using the method of Daniels and Skipper^58^. For each culture, approximately 10 mL of harvested were well suspended in 20 mL of water in a 50-mL Falcon tube. A 25 mL sucrose solution (70% v/w) was injected into the bottom of the tube, forming a stepped density gradient that was centrifuged at 900 × *g* for 3 minutes. Spores of AMF were collected from the interface of the sucrose solution, washed with tap water on a 36 µm sieve for 2 minutes, and transferred to Petri dishes. Spores were picked individually under a stereomicroscope and transferred to Petri dishes with ultrapure water. Spores were sonicated for 15 seconds at 40 kHz of output frequency to remove debris from the spore surface and washed two times with ultrapure water. This extraction step was common for microscopy, molecular and MALDI proteomic analysis.

### Microscopic analysis

Before being used for proteomics and molecular analyses, spores of each specimen were checked for quality and homogeneity control and photographed using a digital camera (Leica DFC 295) on a compound microscope (Olympus BX 50) equipped with Normarski differential interference contrast optics using Leica Application Suite Version V 4.1 software (Supplementary Fig. 1).

### Molecular analysis

For the six New Caledonian isolates and those from the IBG for which no sequence of the SSU-ITS-LSU was available (BEG247, BEG248), one to three spores of each species from the single species cultures on *S*. *vulgare* were ground with a pipette tip in a 1.5-mL Eppendorf tube containing 10 µL of ultrapure water; 2 µL was used for polymerase chain reaction (PCR). A DNA fragment of 1,545 base pairs covering partial SSU, the whole ITS and the variable D1 and D2 regions of the LSU were amplified by nested PCR using AMF-specific primers developed by Krüger *et al*.^18^. In the first round of PCR, the primers SSUmAf and LSUmAr were used. In the second, nested round of PCR, the primers SSUmCf and LSUmBr were used with 1 µL of the first PCR round product as a template. The PCR mix included 0.4 U of AmpliTaq® 360 DNA polymerase (Applied Biosystems), 1X AmpliTaq® 360 PCR buffer (Applied Biosystems), 0.2 mM of each dNTP, 0.4 μM of each primer and 1 µL of the template in a final volume of 25 µL. The cycling parameters for the first PCR were: 3 minutes at 98 °C followed by 30 cycles of 10 seconds at 98 °C, 30 seconds at 60 °C, and 1 minute at 72 °C. The program was concluded by a final extension phase of 10 minutes at 72 °C. The cycling parameters for the second PCR were the same as in the first PCR, except for the number of cycles (35) and the annealing temperature (63 °C). The PCRs were conducted in triplicate. PCR products were checked on 1% agarose gels and stained with ethidium bromide. The positive PCR products (70 ng) were cloned into pGEM-T using a pGEM-T easy vector system (Promega) following the manufacturer’s instructions. Ligated plasmids were transformed into CaCl_2_-competent *E*. *coli* DH5α cells using a heat-shock approach. The transformed bacteria were plated into LB (Luria Bertani) medium containing ampicillin (50 μg⁄mL) and grown overnight at 37 °C. For each AMF isolate, three independent recombinant clones were sequenced using an Applied Biosystems 3730xl capillary sequencer (IRD, Noumea) with the BigDye® Terminator v3.1 Cycle Sequencing Kit (Applied Biosystems). The forward and reverse strands were assembled in ChromasPro (Technelysium Pty Ltd, Australia). The glomeromycotan origin of the sequences was tested by BLAST^59^. The new sequences were deposited in the EMBL database under the accession numbers KY362428, KY362429, KY362430, KY362431, KY362432, KY362433, KY362434, KY362435, KY362436, KY362437, KY362438, KY927386, KY927387, KY934450, KY934451, KY934452, KY927391, KY927390, KY927388, and KY927389.

### Phylogenetic analysis

DNA sequences were aligned using MAFFT 7 (http://mafft.cbrc.jp/alignement/server; Katoch and Standley^60^) with the slow iterative refinement option FF-NS-I (gap opening penalty 1.0, offset value 0.1). Maximum likelihood (ML) analyses of the partial region (partial SSU-5,8S-LSU partial) or the partial 28 S part were performed using PhyML 3.0^61^ and MEGA 7 software^62^ with bootstrap support obtained using 1000 replicates. ITS1 and ITS2 were trimmed as explained in Krüger *et al*.^19^. Phylogenetic trees were viewed and edited using MEGA 7 software^63^.

### MALDI-TOF-MS sample preparation

First, we tested the detection limit of MALDI-TOF using *Scutellospora ovalis* with 1, 2, 4, 5 and 10 spores for the analyses to determine the optimal signal-to-noise ratio. The number of spores per sample for the other isolates was calculated using the estimated spore volume of each isolate (Supplementary Table S1). The final protocol was designed considering the protocols developed for pathogenic yeasts^64^ and cyanobacteria^32^. Spore proteins were extracted using formic acid (FA) as follows: fungal spore samples were transferred to a sterile 1.5-mL tube with 20 µL of ultrapure water. After centrifugation for 3 minutes at 13,000 × g, the supernatant was discarded and the spore pellet was incubated for 5 minutes in 10 µL of (70:30 [vol/vol]) formic acid/acetonitrile (Sigma-Aldrich, Lyon, France). Spores were crushed using a pestle for 30 seconds and incubated for 5 minutes at room temperature before use. Each sample was checked under a stereomicroscope to ensure that spores were completely crushed. Successive aliquots of 1.5 μL of the supernatant were transferred to a polished steel MSP 96 target (Bruker) until the sample was consumed and was allowed to dry at room temperature before being overlaid with 1 μL of a saturated *a*-cyano-4-hydroxycinnamic acid (HCCA) matrix solution in 50% acetonitrile-2.5% trifluoroacetic acid (Bruker).

### Data acquisition

Measurements were performed on a Biotyper CA System that includes a Bruker benchtop microflex™ MALDI-TOF (Matrix Assisted Laser Desorption Ionization-Time of Flight) software. A Bruker Bacterial Test Standard (BTS0000218558; Bruker) was used for instrument calibration and performance verification. Spectra were recorded in the positive linear mode (laser frequency, 20 Hz; ion source 1 voltage, 20 kV; ion source 2 V, 16.7 kV; lens voltage, 8.5 kV; mass range, 2000–20000 Da).

### Data processing

Spores of the investigated AMF isolates were recorded as MSP references using the automated functionality of the MALDI Biotyper package (Bruker MBT 3.1 software). For each database entry, eight biological replicates were individually measured three times. A selection of 20 mass spectra were imported into the software, which performs normalization, smoothing, baseline correction and peak picking, generating a list of the most significant peaks and calculating a main spectrum containing the average peak mass (m/z: m stands for mass and z stands for charge number of ions), the average peak intensity and frequency information. In a first step to assess the uniqueness of our spectra, we checked for overlaps of our database with the Bruker database included with the MALDI Biotyper 3.1 software which contains 6903 reference spectra of 2461 microbial species, including human pathogen fungi by merging the two.

Principal component analyses (PCA) for each AMF family and hierarchical cluster analyses were conducted using the integrated tools of the MALDI Biotyper 3.1 software package, using default settings. A graphical analysis of the spectra of the different isolates was carried out and a dendrogram was created using the reference spectra to assess the similarity of their spectra. Isolates with distance levels under 500 (arbitrary units) have been described as reliably conspecific, whereas isolates with a distance level under 100 are considered clones^31^.

### MALDI-TOF MS real-time blind test, data interpretation

For each isolate, three biological replicates of spores were prepared as explained above. The MALDI Biotyper CA System Sample Workflow (the internal software), compares each sample mass spectrum to the reference mass spectra in the MSP database and calculates a unit score value (Main Pattern score or MP score) between 0 and 3, reflecting the similarity between sample and reference spectrum. The top 10 matching database records are displayed. As specified by the manufacturer (Bruker) for bacteria identification, scores ≥2.0 are generally accepted for a reliable identification at the species level, and scores ≥1.7 and <2.0 were used for identification at the genus level. Similar to Nonnemann *et al*.^65^, we tried to investigate whether it was possible to lower the log score. This previous work suggested that the log score can be lowered to 1.5 when identifying pure cultures.

### MALDI-TOF-MS data export and identification by graphical analyses on R software

All MSPs data were exported in m/z XML format to the software R, version 3.3.2^30^ using the “tcltk, XML, reshape2, ggplot2, ggrepel” packages. MSP in Bruker software consists of a peak list of a selection of the 100 “best” peaks and are used for calculation and scoring in real time analysis. The export of these data makes it possible to obtain a graphical representation of the MSPs and perform a graphical comparison between reference spectra and unknown sample spectra. For further investigation, a mixed sample of two isolates (one spore of *G*. *rosea* BEG9; one spore of *G*. *margarita* BEG34) was prepared and analysed as explained above, followed by a graphical comparison of the obtained spectra to MSP of each study’s isolates.

### Possible influence of culture conditions, age or storage

We assessed the possible influence of the culturing substrate on spectra by analysing spores of *Claroideoglomus etunicatum nc* from commercial compost and ultramafic soils. Possible storage effects were addressed by comparing spectra from spores of this species after three months of storage in dry soil and spores from a living culture (humid soil). To address the effect of culture age we compared spores of *C*. *etunicatum* BEG247 from cultures set up in 2011 and 2014. Finally, *Funneliformis mosseae* BEG12 was chosen to investigate the influence of spore maturity because, in this species, it is very easy to differentiate juvenile and mature spores; juvenile spores are clearly smaller than mature spores (Supplementary Fig. 1). We used four mature spores to compare to eight juvenile spores. All spore samples were prepared as explained above in three replicates. Graphical analysis of spectra resulting from spores from different culture substrates, subculture age, storage conditions and different maturity stages were performed as described above.

## Electronic supplementary material

Revised Supplementary Information - Crossay et al. ref.SREP-17-18530
