# Supplementary material for: New method for the identification of arbuscular mycorrhizal fungi by proteomic-based biotyping of spores using MALDI-TOF-MS
Source: Sci Rep. 2017 Oct 30;7:14306. doi: 10.1038/s41598-017-14487-6 (PMC5662746; doi:10.1038/s41598-017-14487-6)
Supplement: Supplementary file 1 — Revised Supplementary Information - Crossay et al. ref.SREP-17-18530 [file 41598_2017_14487_MOESM1_ESM.pdf]

Supplementary Information

New method for the identification of arbuscular mycorrhizal fungi by proteomic-based biotyping of spores using MALDI-TOF-MS

Thomas Crossay[1], Cyril Antheaume[1,2,*], Dirk Redecker[3], Lucie Bon[1], Nicolas Chedri[4], Clément Richert[5], Linda Guentas[1], Yvon Cavaloc[1], Hamid Amir[1,*]

[1] *Institut des Sciences Exactes et Appliquées (EA 7484), Université de Nouvelle-Calédonie, BP R4, 98851 Nouméa, New-Caledonia*
[2] *Plate-forme d'Analyse Chimique Strasbourg-Illkirch. Université de Strasbourg, F-67400 Illkirch, France*
[3] *Agroécologie, AgroSup Dijon, CNRS, INRA, Univ. Bourgogne Franche-Comté, F-21000 Dijon, France*
[4] *Institut Pasteur, Bacteriology Research Unit, 98800 Nouméa, New-Caledonia*
[5] *Bruker Corporation, France – New-Caledonia*

*Corresponding authors: hamid.amir@univ-nc.nc, cyril.antheaume@gmail.com

Tel: +(687) 290241

# Current address: Université de Nouvelle-Calédonie, BP R4, 98851 Nouméa Cédex, Nouvelle-Calédonie

Isolates	Origin	Diameter of spore µm	Approximate volume of 1 spore µm3	Nb spores per sample
Gigaspora rosea BEG 9	**United States**	**250**	**8175040**	**1**
Gigaspora margarita BEG 34	**New Zealand**	**300**	**14126470**	**1**
Gigaspora candida BEG 17	**Taiwan**	**230**	**6365800**	**1**
Scutellospora calospora BEG 32	**England**	**115**	**795730**	**3**
Rhizophagus clarus BEG 248	**Brazil**	**150**	**1765810**	**1**
Rhizophagus clarus BEG 149	**China**	**150**	**1765810**	**1**
Rhizophagus clarus BEG 142	**Brazil**	**150**	**1765810**	**1**
Funneliformis mosseae BEG 12	**England**	**110**	**696380**	**4**
Funneliformis coronatum BEG 22	**Australia**	**150**	**1765810**	**1**
Claroideoglomus etunicatum BEG 247	**Brazil**	**85**	**321310**	**7**
Claroideoglomus claroideum BEG 31	**Finland**	**100**	**523200**	**4**
Claroideoglomus claroideum BEG 14	**Denmark**	**105**	**605672**	**4**
Claroideoglomus claroideum BEG 23	**Czech Republic**	**100**	**523200**	**4**
Scutellospora ovalis	**New Caledonia**	**100/219**	**1135350**	**2**
Acaulospora saccata	**New Caledonia**	**75**	**220730**	**10**
Acaulospora fragilissima	**New Caledonia**	**67**	**157360**	**14**
Rhizophagus neocaledonicus	**New Caledonia**	**75**	**220730**	**10**
Claroideoglomus etunicatum nc	**New Caledonia**	**92,5**	**414090**	**6**
Pervetustus simplex nc	**New Caledonia**	**53**	**77890**	**29**

Supplementary Table 1. Species used, their origin, measured spore diameter (n=100), calculated volume of spores required for proteomic analysis, number of spores used per sample for proteomic analysis. New-Caledonian isolates were provided by the Laboratoire Insulaire du Vivant et de l'Environnement (LIVE), other isolates were provided by International Bank for the Glomeromycota (IBG).

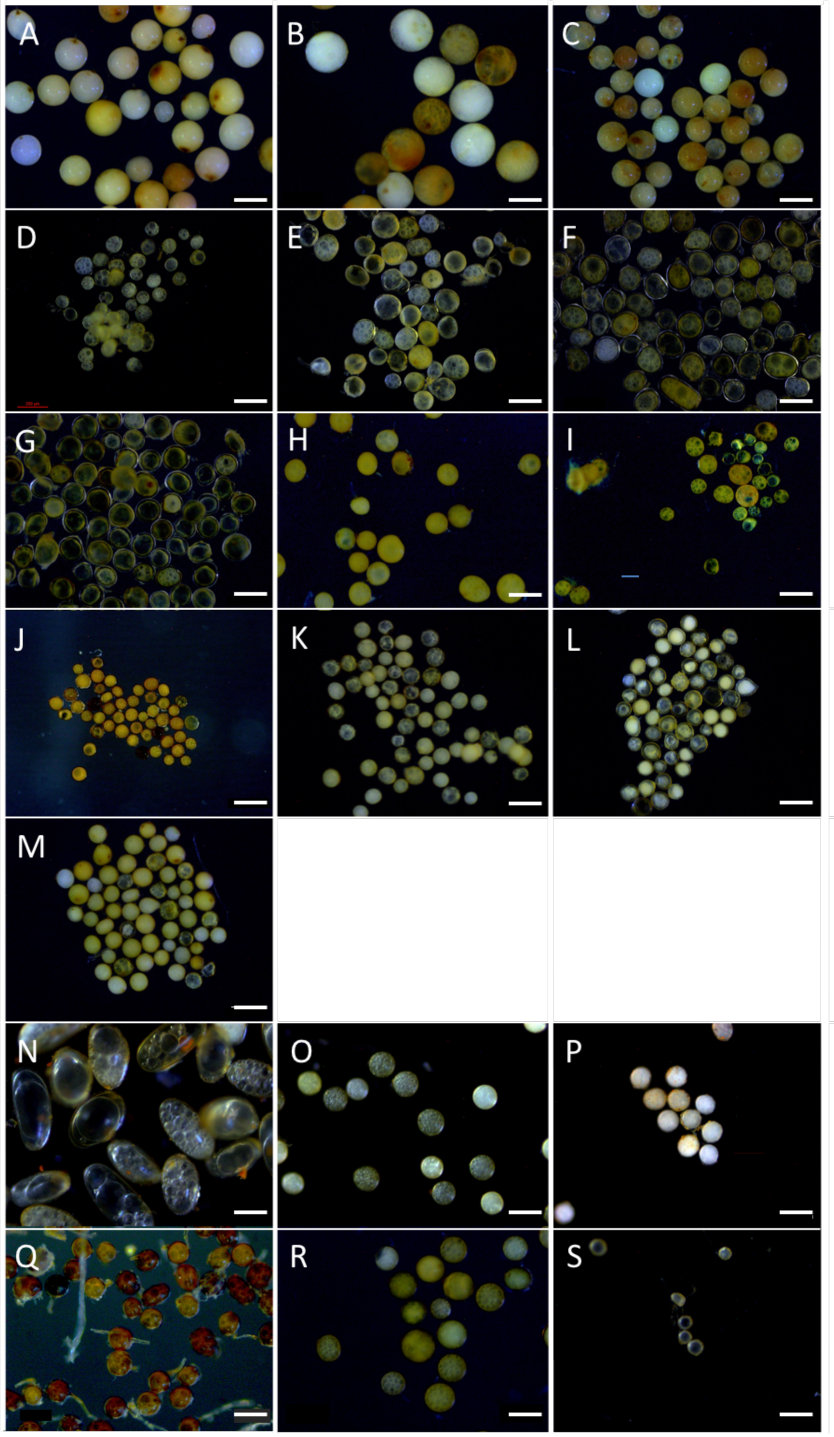

Supplementary Figure 1. Intact spores of studied isolates. A. *Gigaspora rosea* BEG9*;* B. *Gigaspora margarita* BEG34; C. *Gigaspora candida* BEG17; D. *Scutellospora calospora* BEG32; E. *Rhizophagus clarus* BEG248; F. *Rhizophagus clarus* BEG149; G. *Rhizophagus clarus* BEG142; H. *Funneliformis coronatum* BEG22; I. *Funneliformis mosseae* BEG12; J. *Claroideoglomus etunicatum* BEG247; K. *Claroideoglomus claroideum* BEG31; L. *Claroideoglomus claroideum* BEG14; M. *Claroideoglomus claroideum* BEG23. N. *Scutellospora ovalis;* O. *Acaulospora saccata;* P. *Acaulospora fragilissima;* Q. *Rhizophagus neocaledonicus;* R. *Claroideoglomus etunicatum nc;* S. *Pervetustus simplex nc.* A-M scale bars = 250 μm; N-S scale bars = 100 μm.

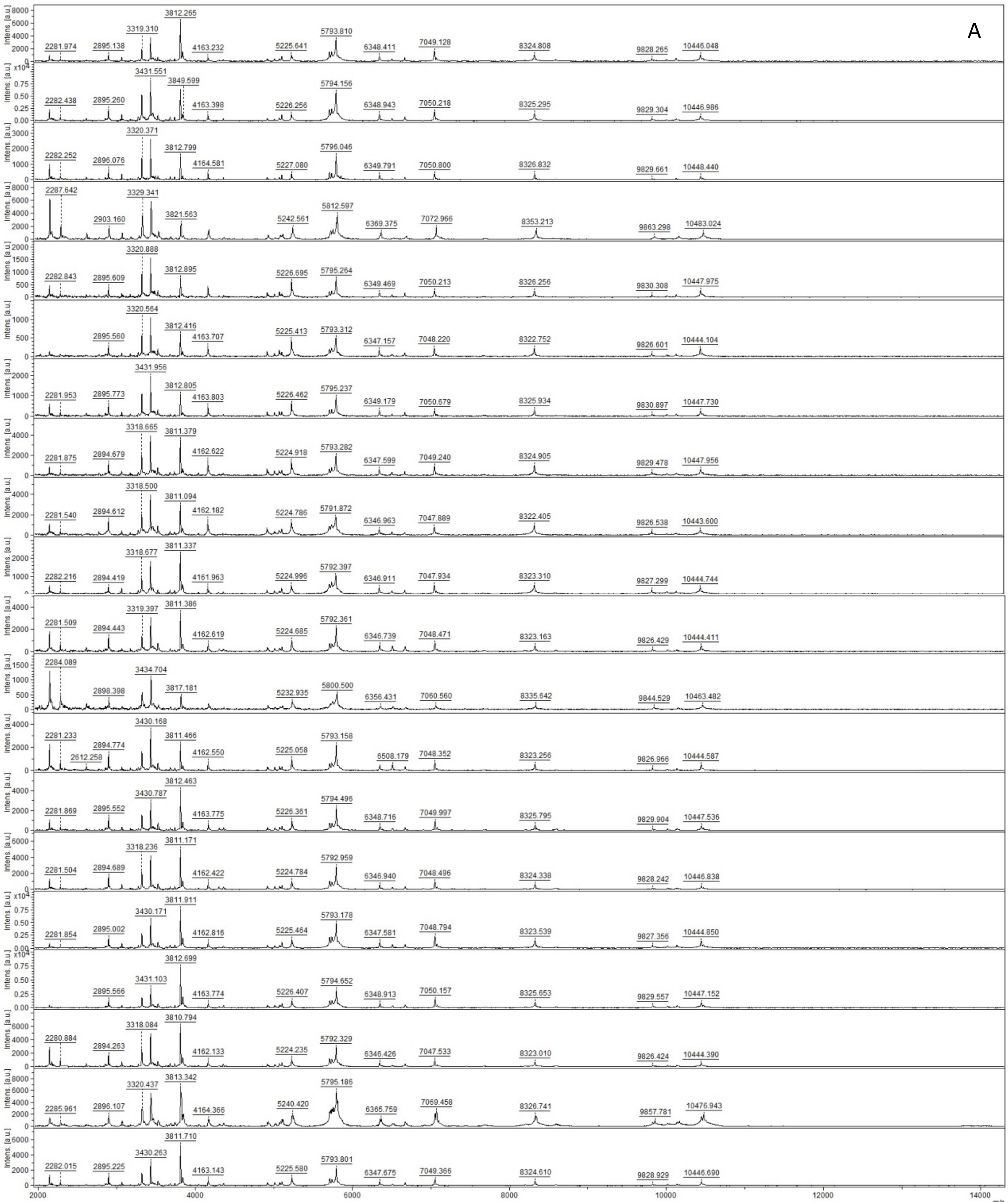

Supplementary Figure 2A. *Gigaspora rosea* BEG9

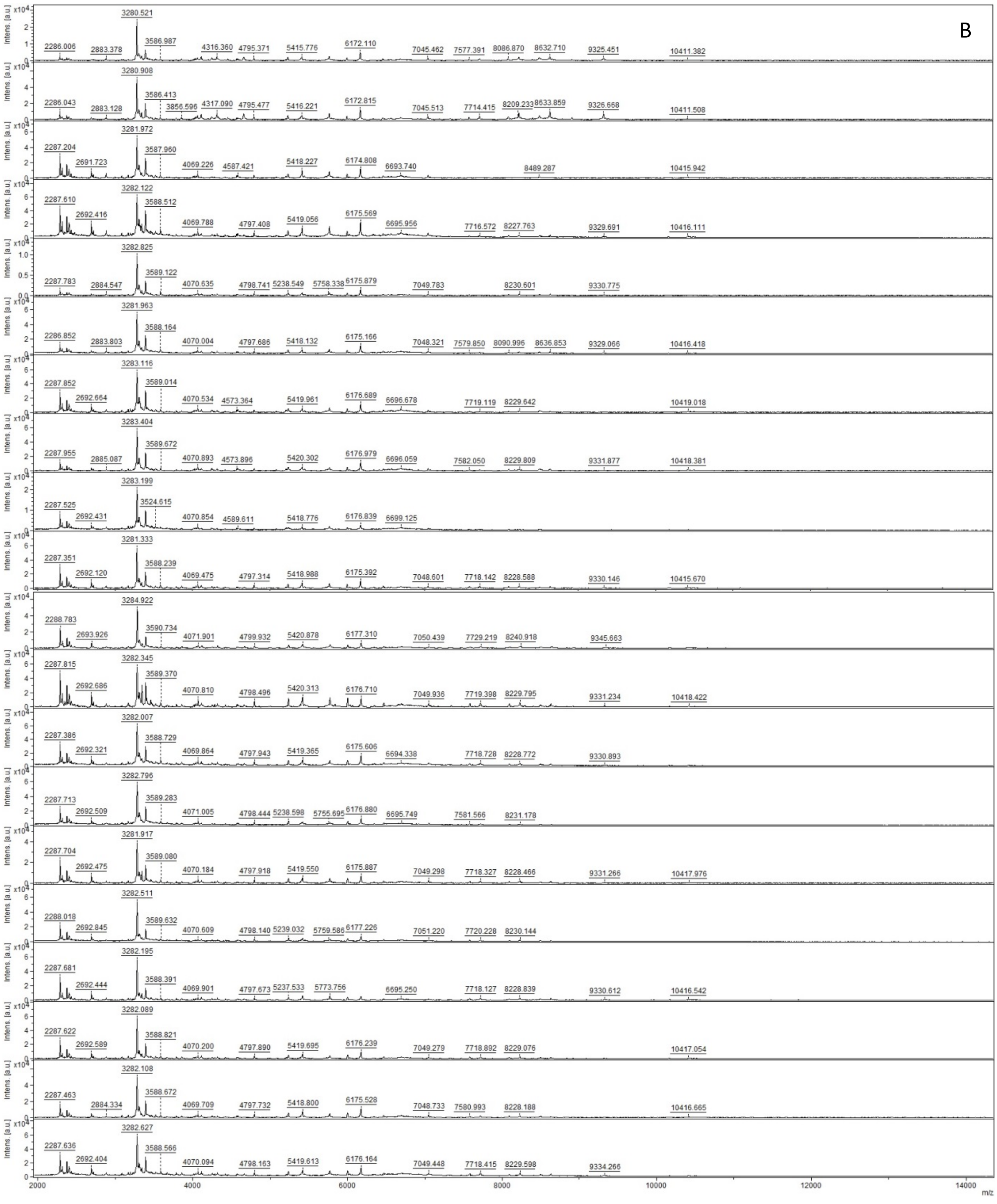

Supplementary Figure 2B. *Gigaspora margarita* BEG34

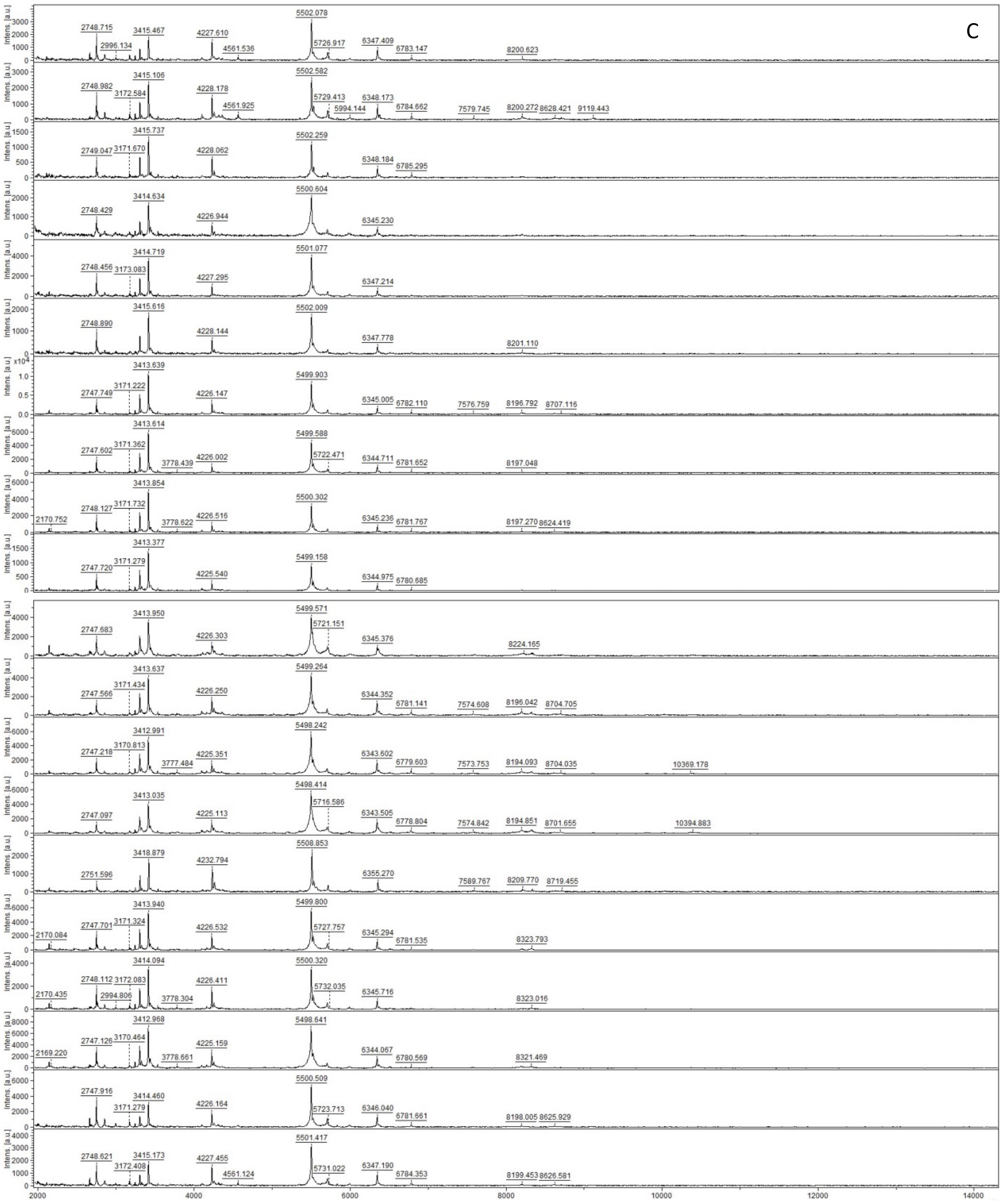

Supplementary Figure 2C. *Gigaspora candida* BEG17

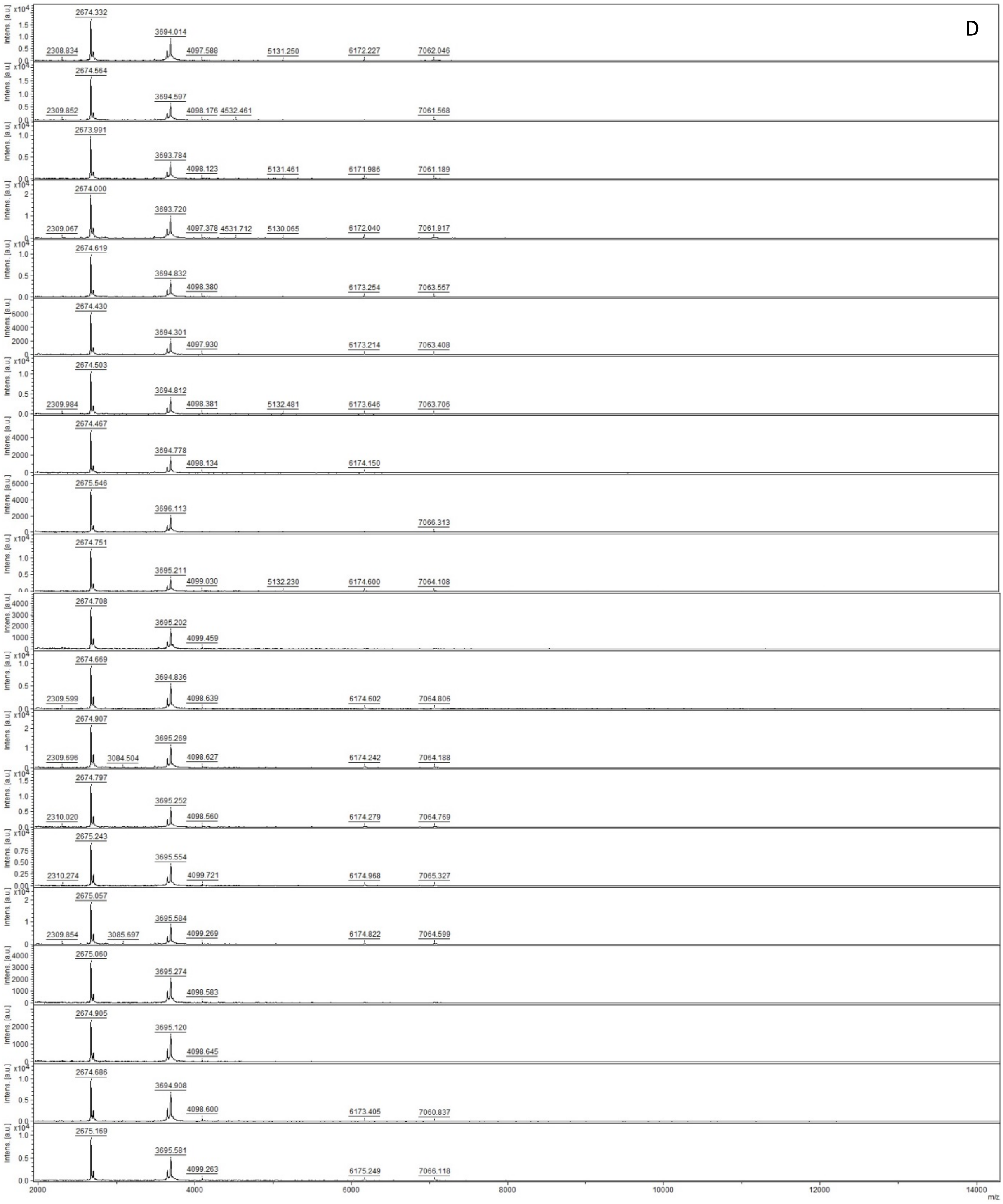

Supplementary Figure 2D. *Scutellospora calospora* BEG32

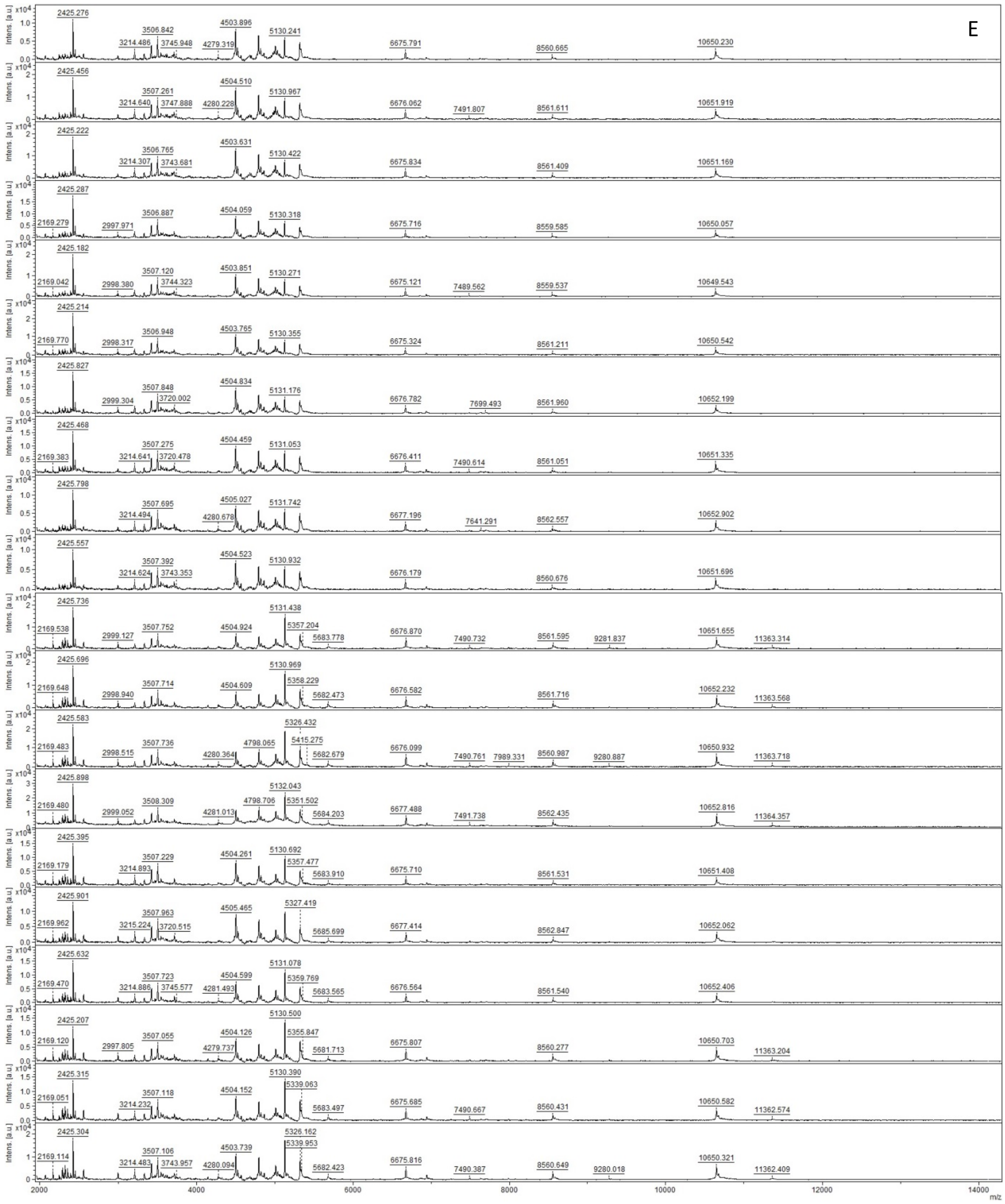

Supplementary Figure 2E. *Rhizophagus clarus* BEG248

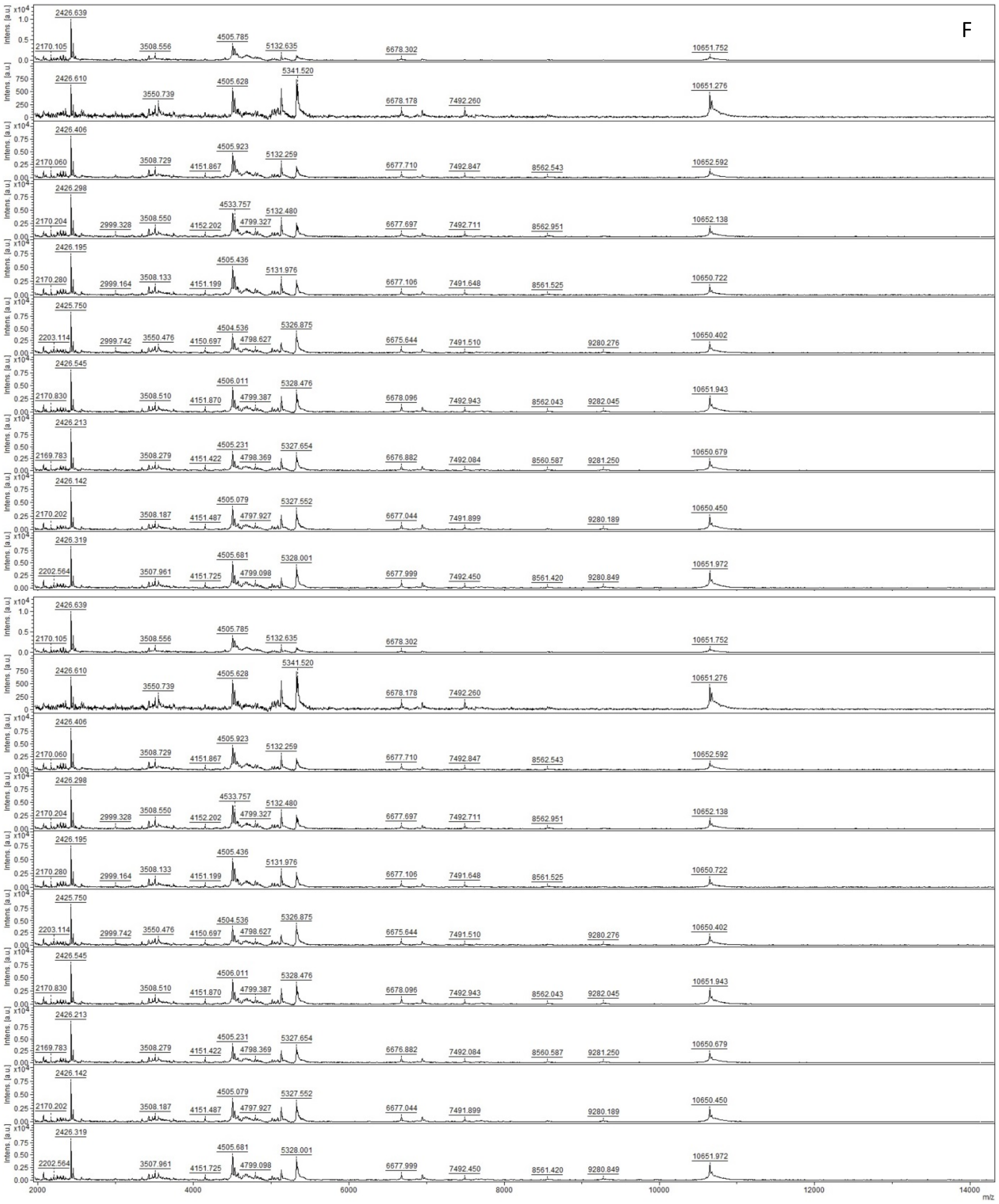

Supplementary Figure 2F. *Rhizophagus clarus* BEG149

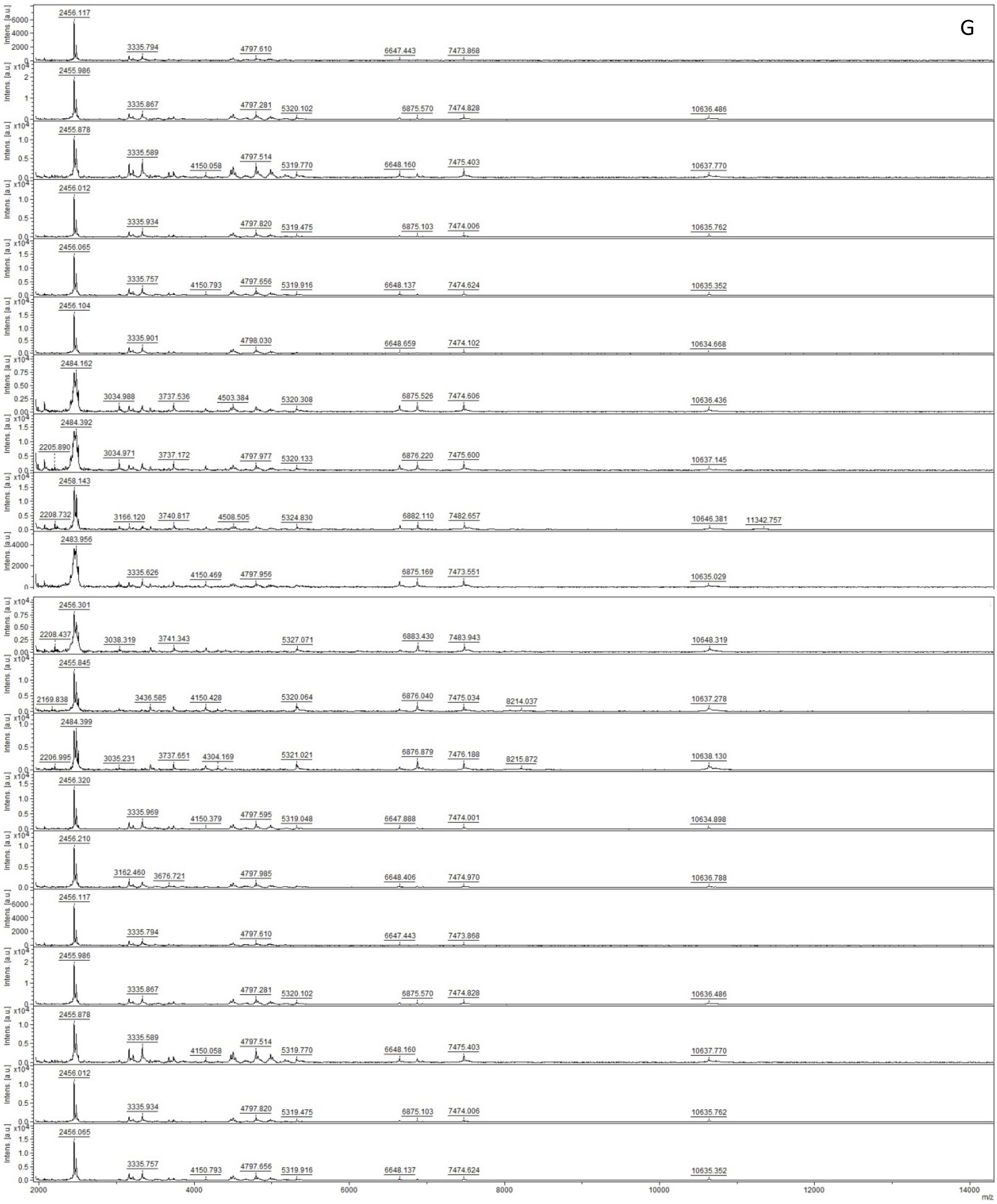

Supplementary Figure 2G. *Rhizophagus clarus* BEG142

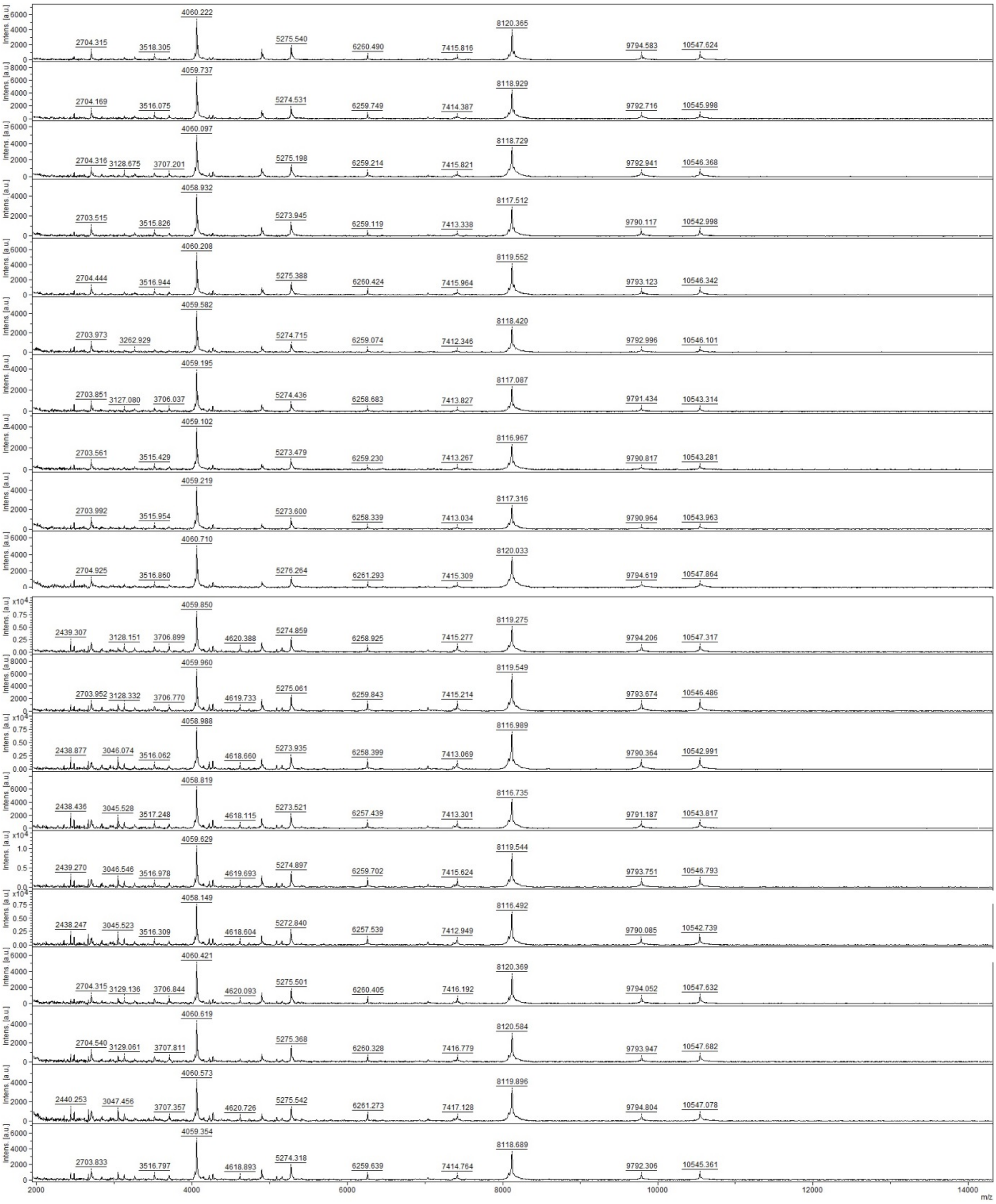

Supplementary Figure 2H. *Funneliformis coronatum* BEG22

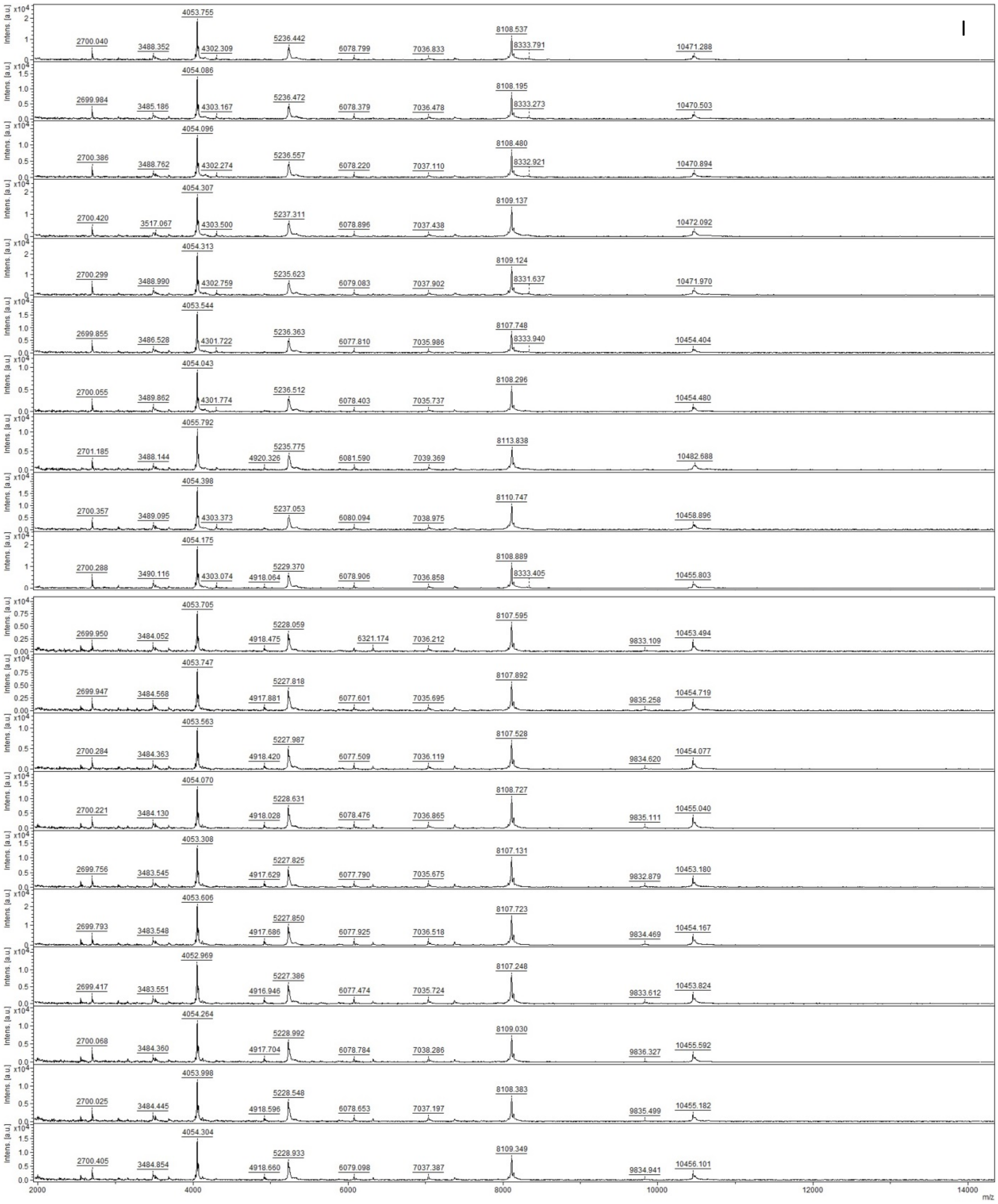

Supplementary Figure 2I. *Funneliformis mosseae* BEG12

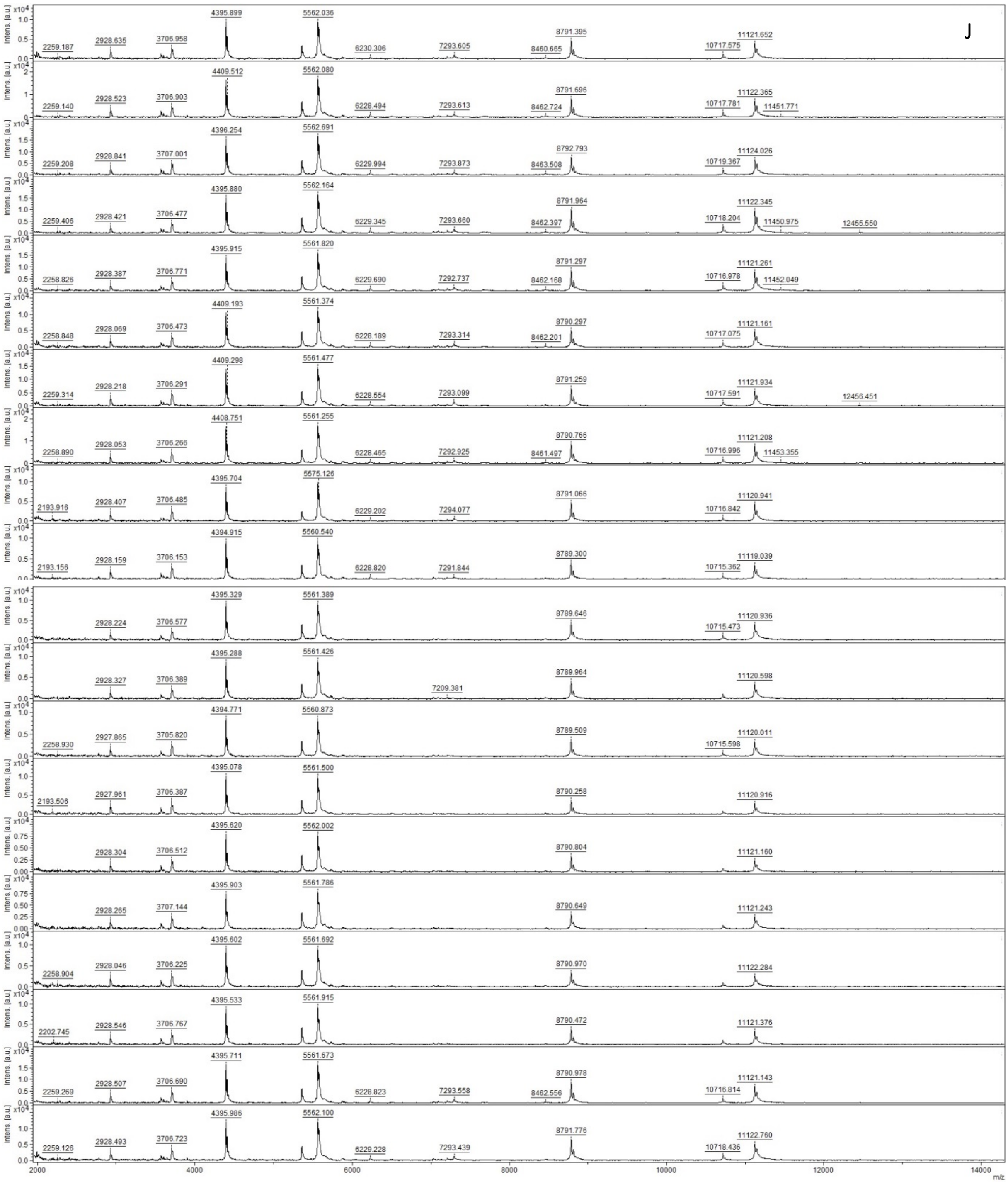

Supplementary Figure 2J. *Claroideoglomus etunicatum* BEG247

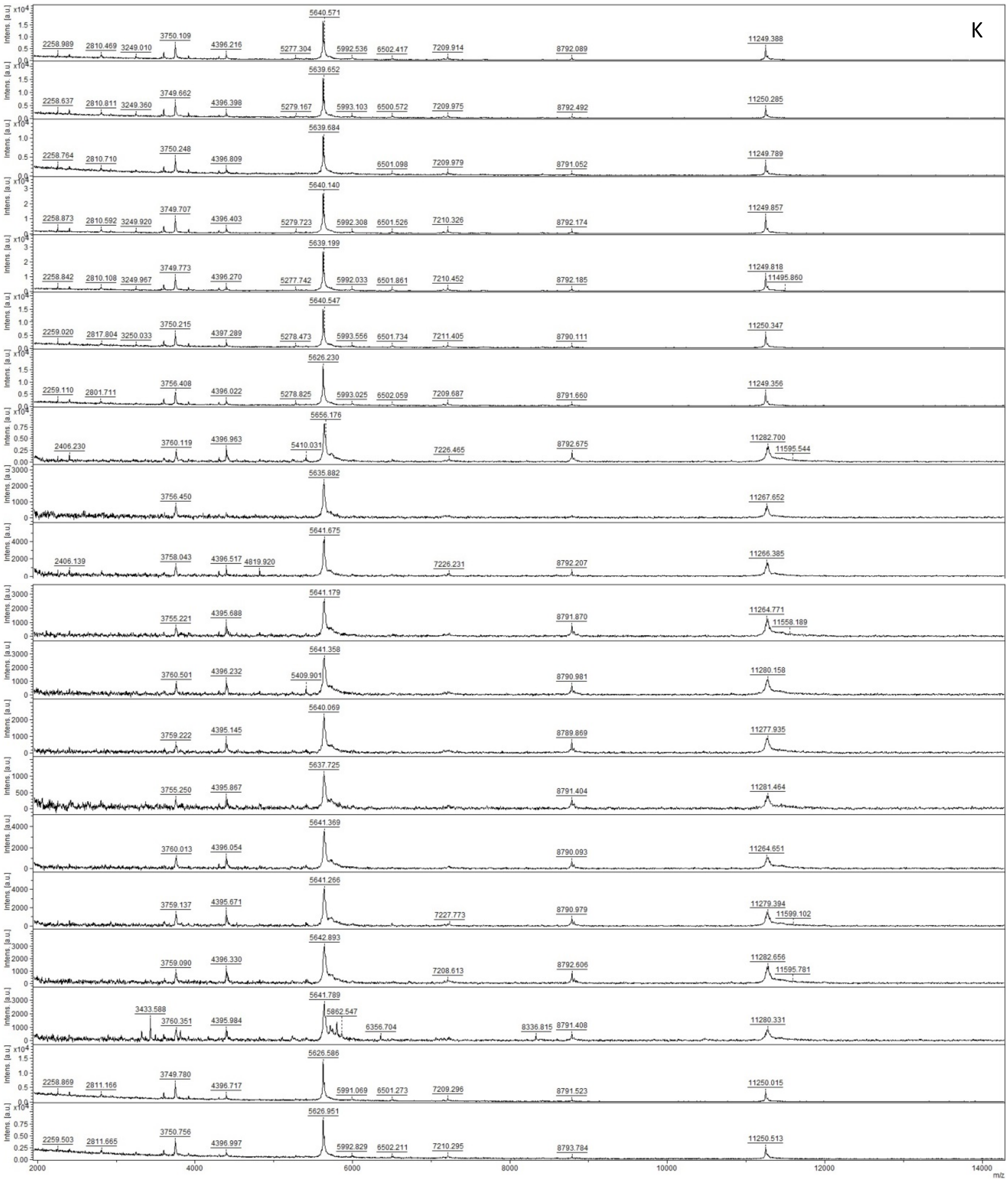

Supplementary Figure 2K. *Claroideoglomus claroideum* BEG31

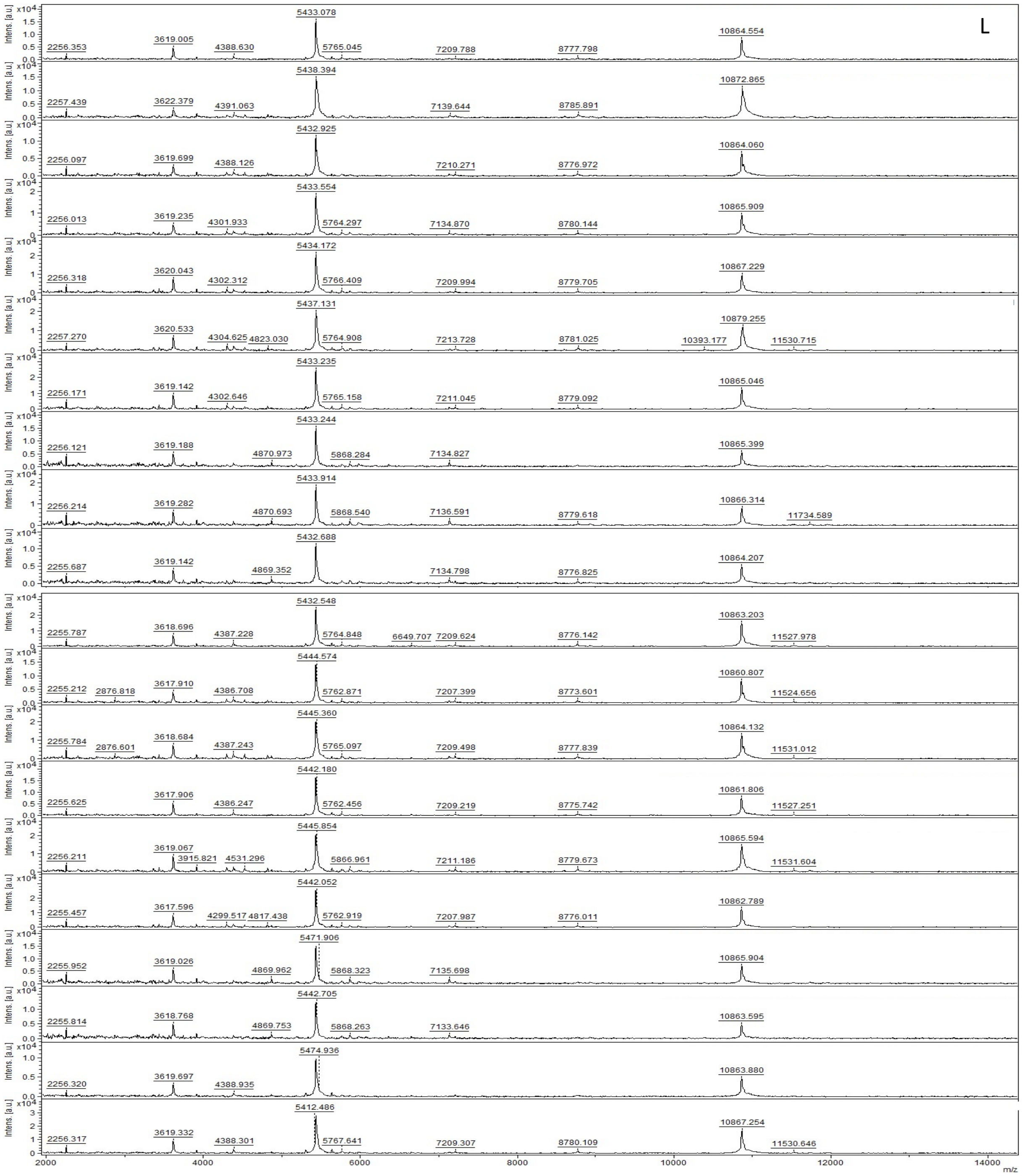

Supplementary Figure 2L. *Claroideoglomus claroideum* BEG14

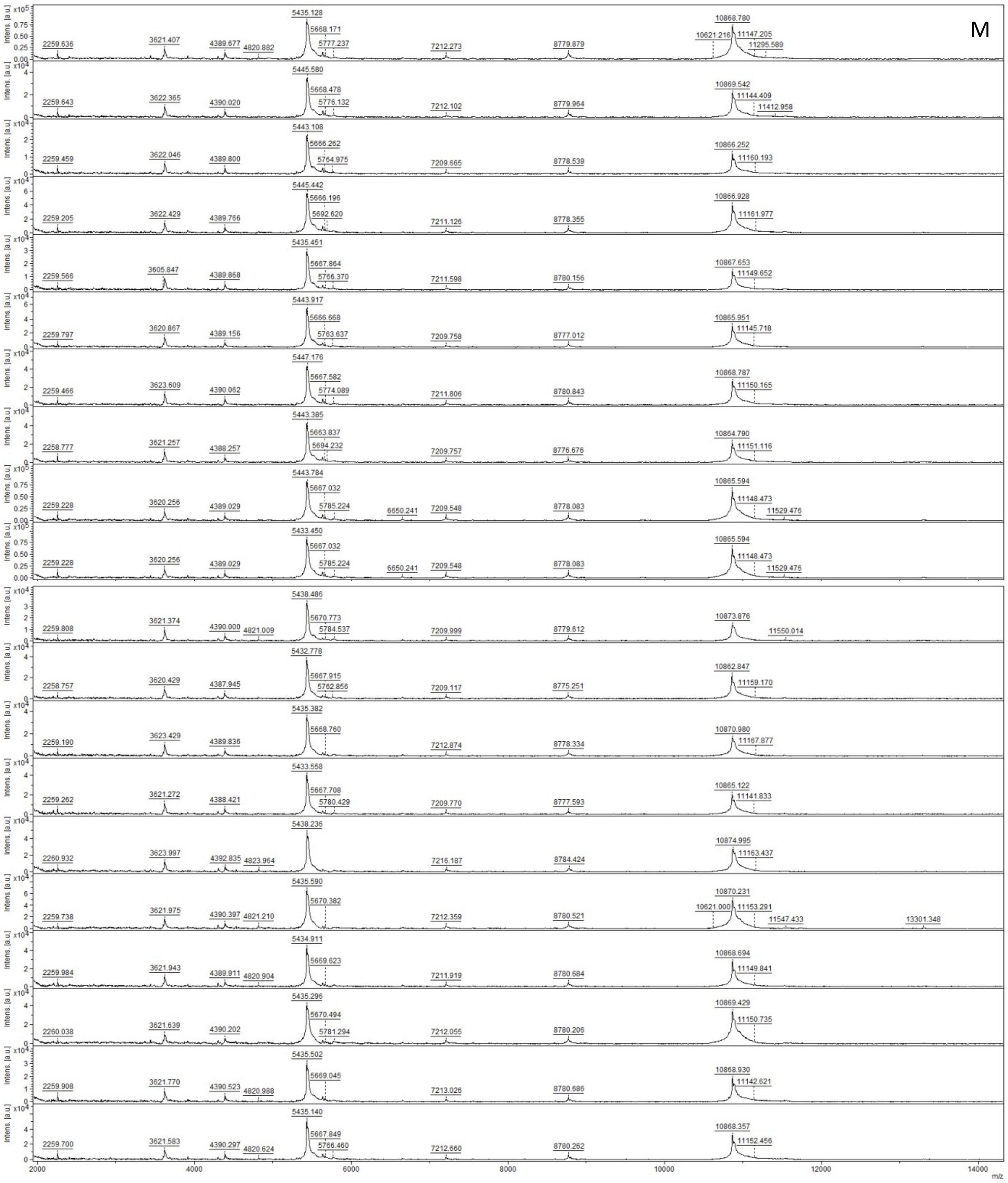

Supplementary Figure 2M. *Claroideoglomus claroideum* BEG23

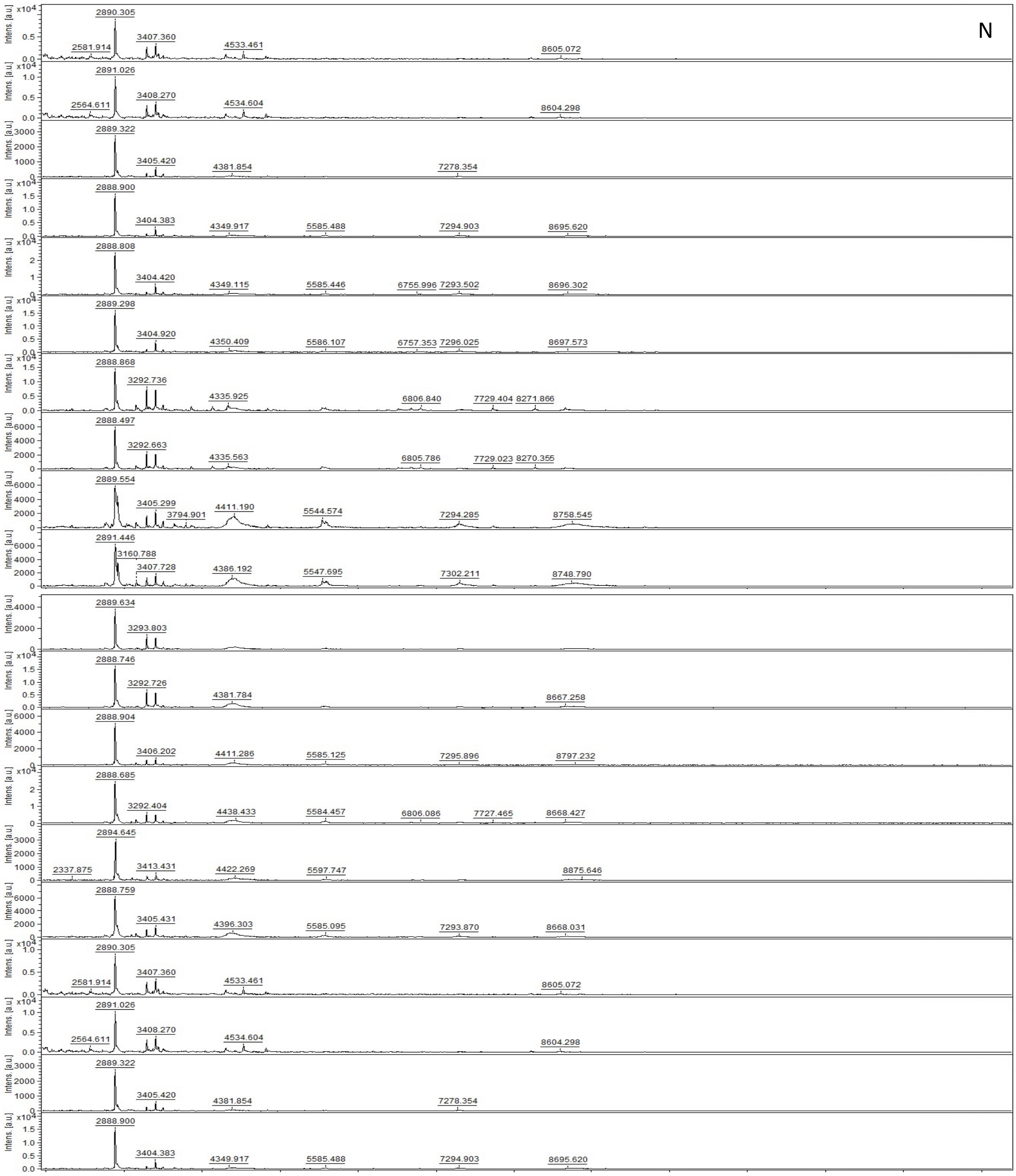

Supplementary Figure 2N. *Scutellospora ovalis*

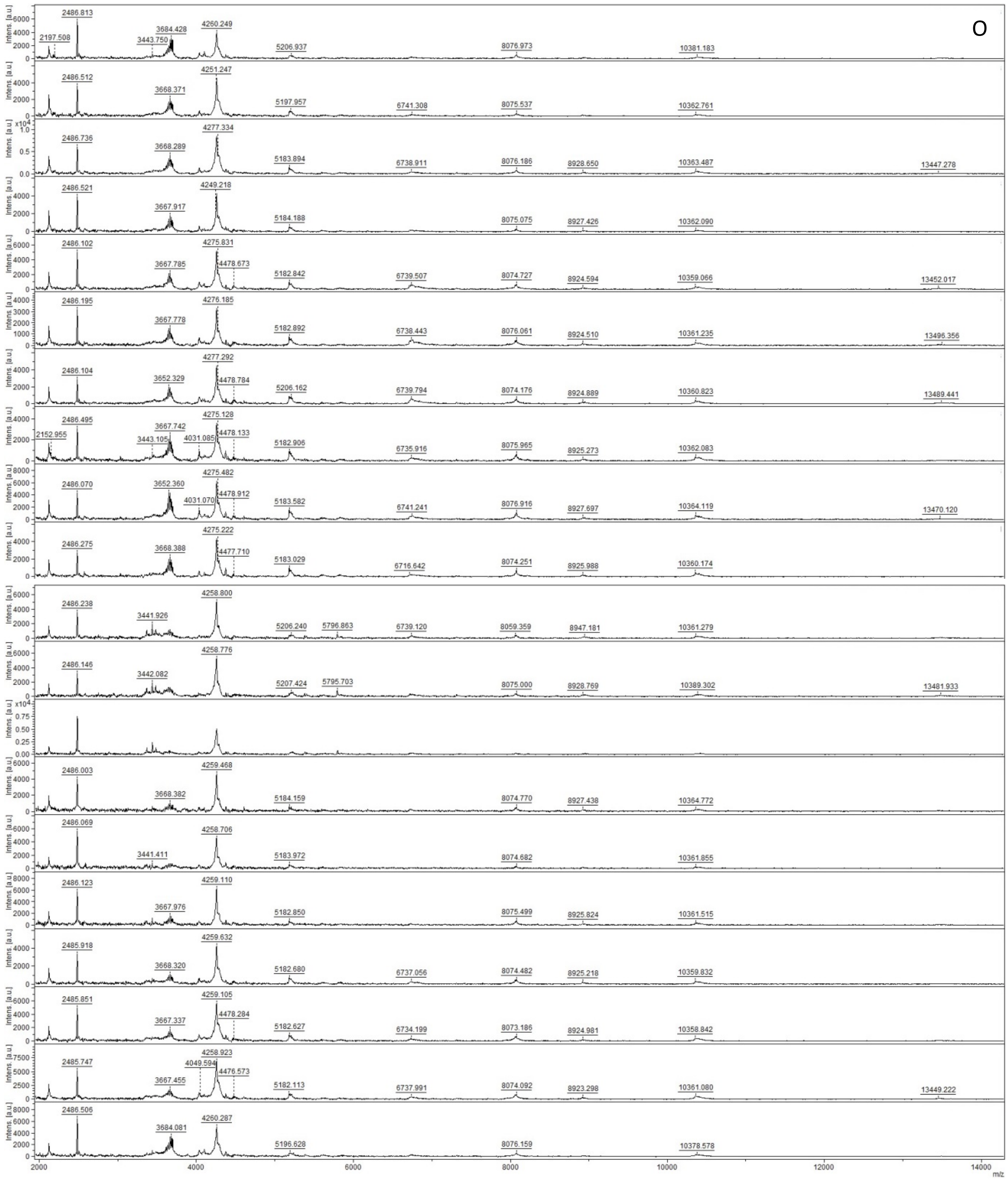

Supplementary Figure 2O. *Acaulospora saccata*

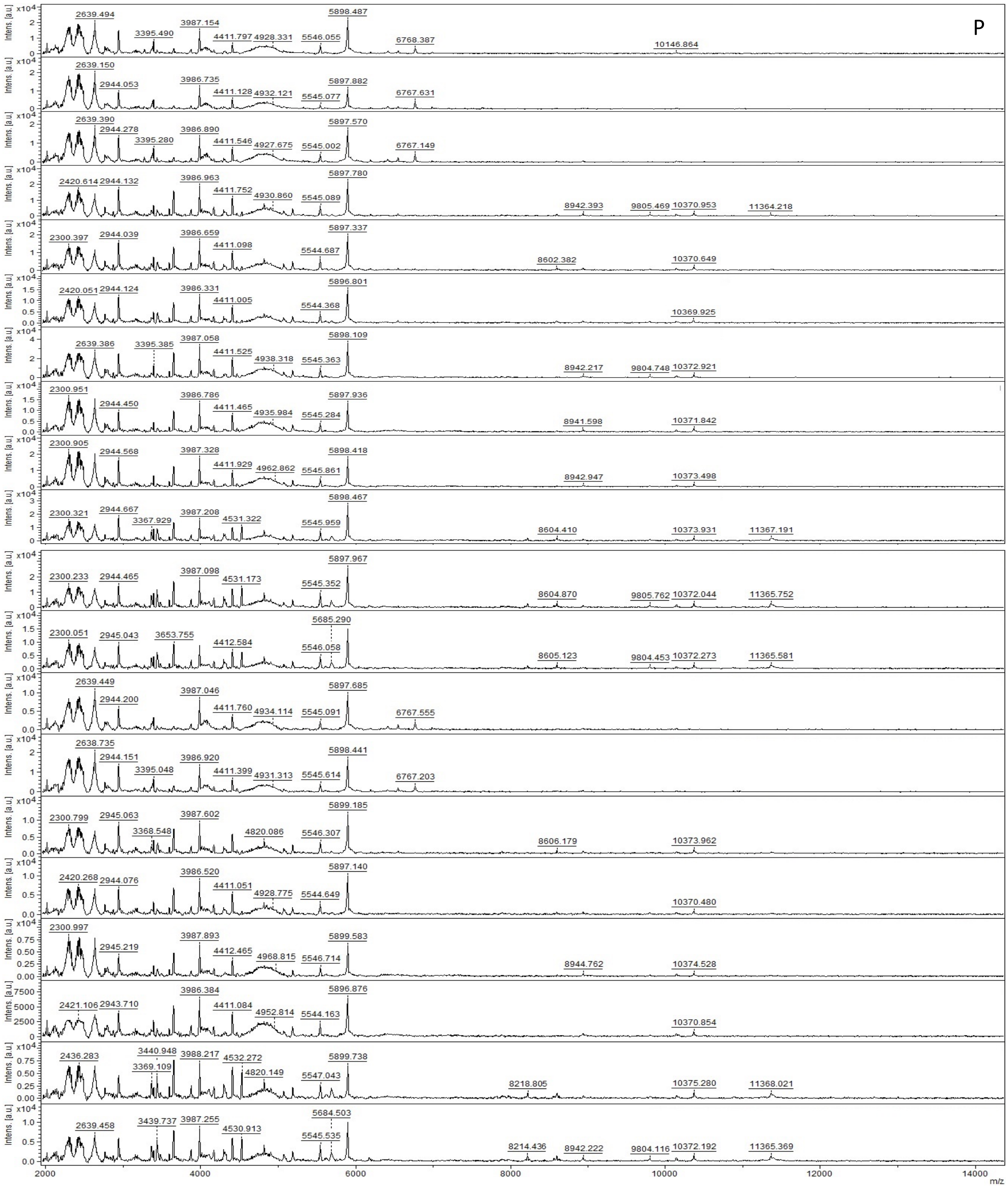

Supplementary Figure 2P. *Acaulospora fragilissima*

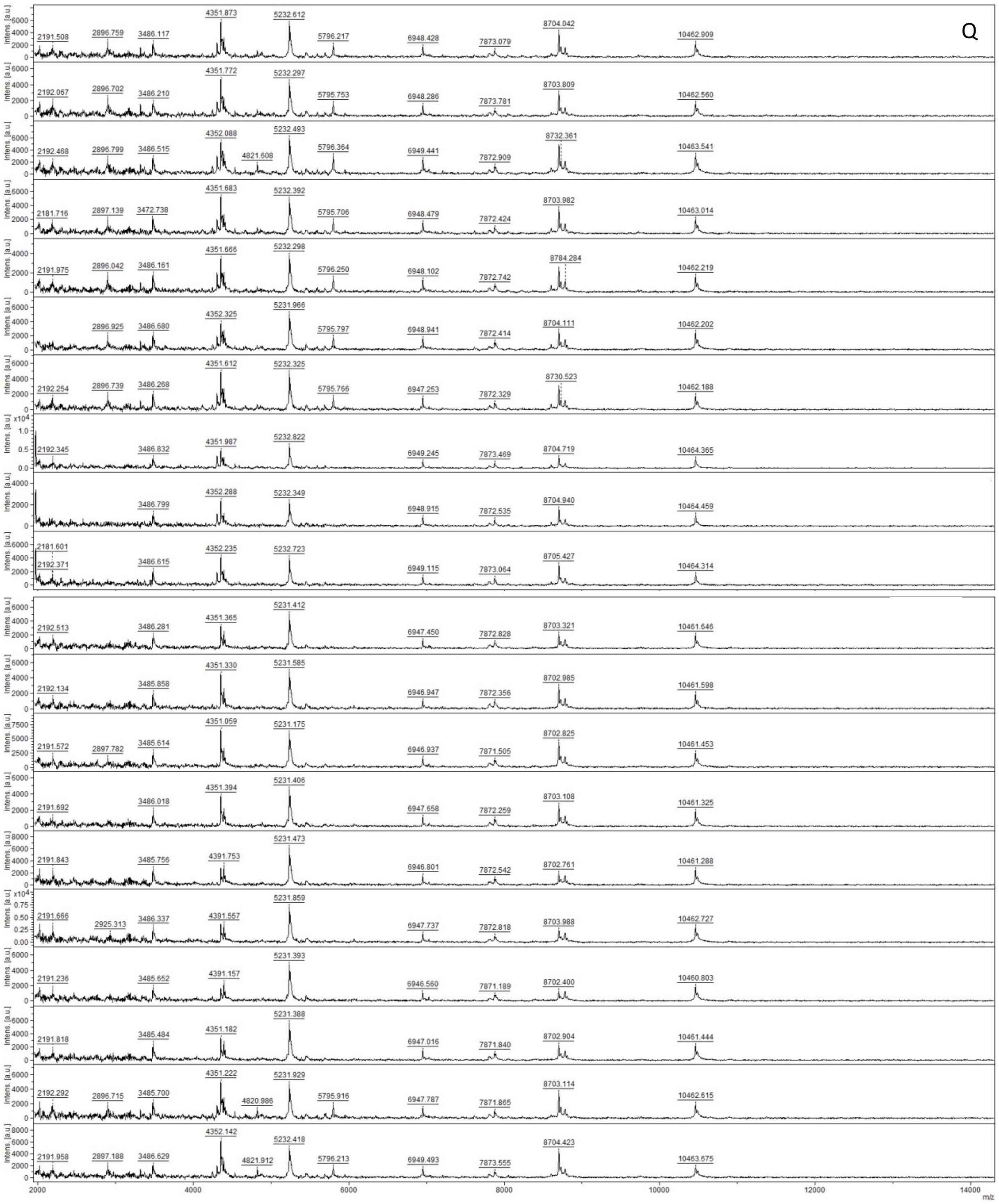

Supplementary Figure 2Q. *Rhizophagus neocaledonicus*

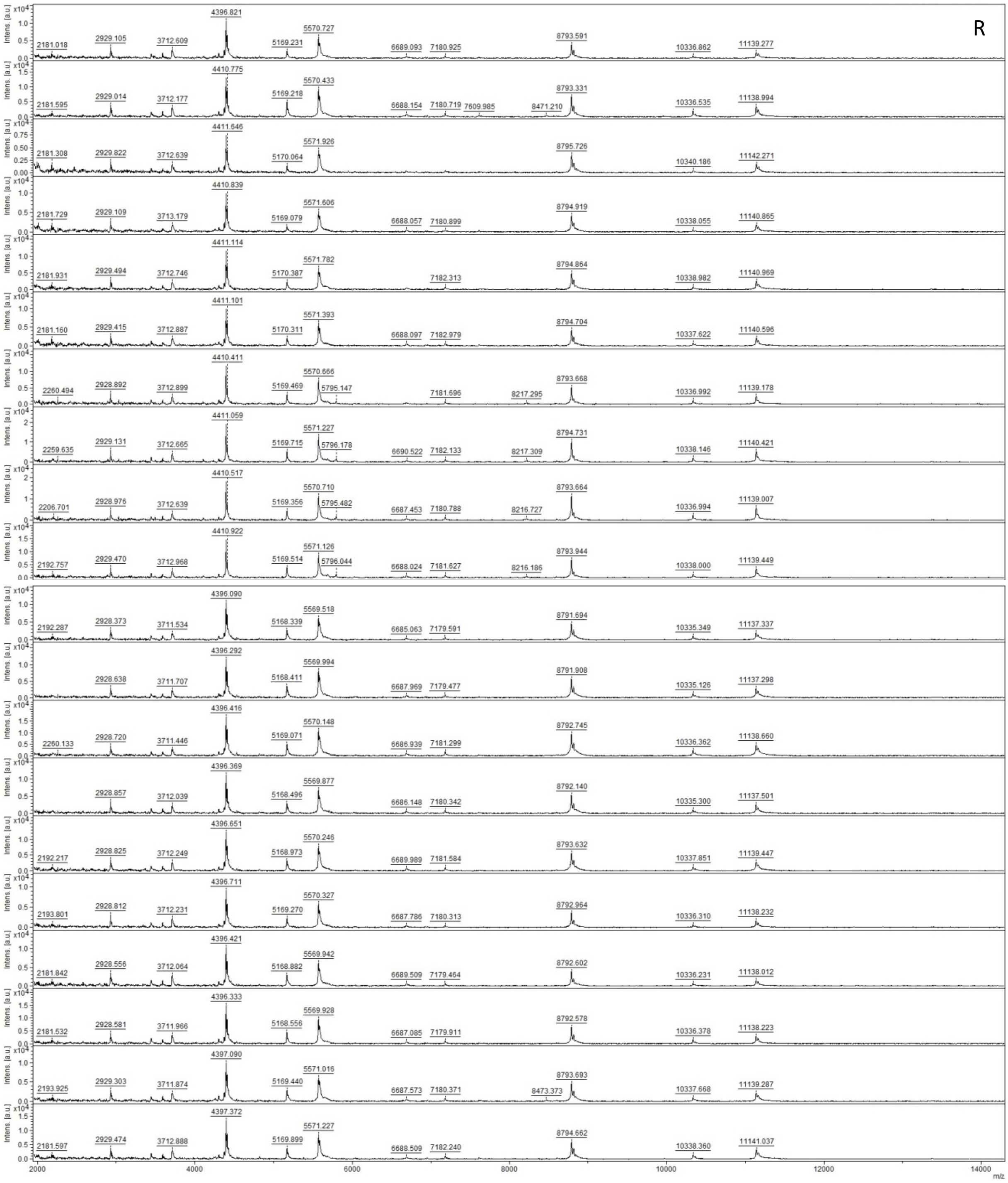

Supplementary Figure 2R. *Claroideoglomus etunicatum nc*

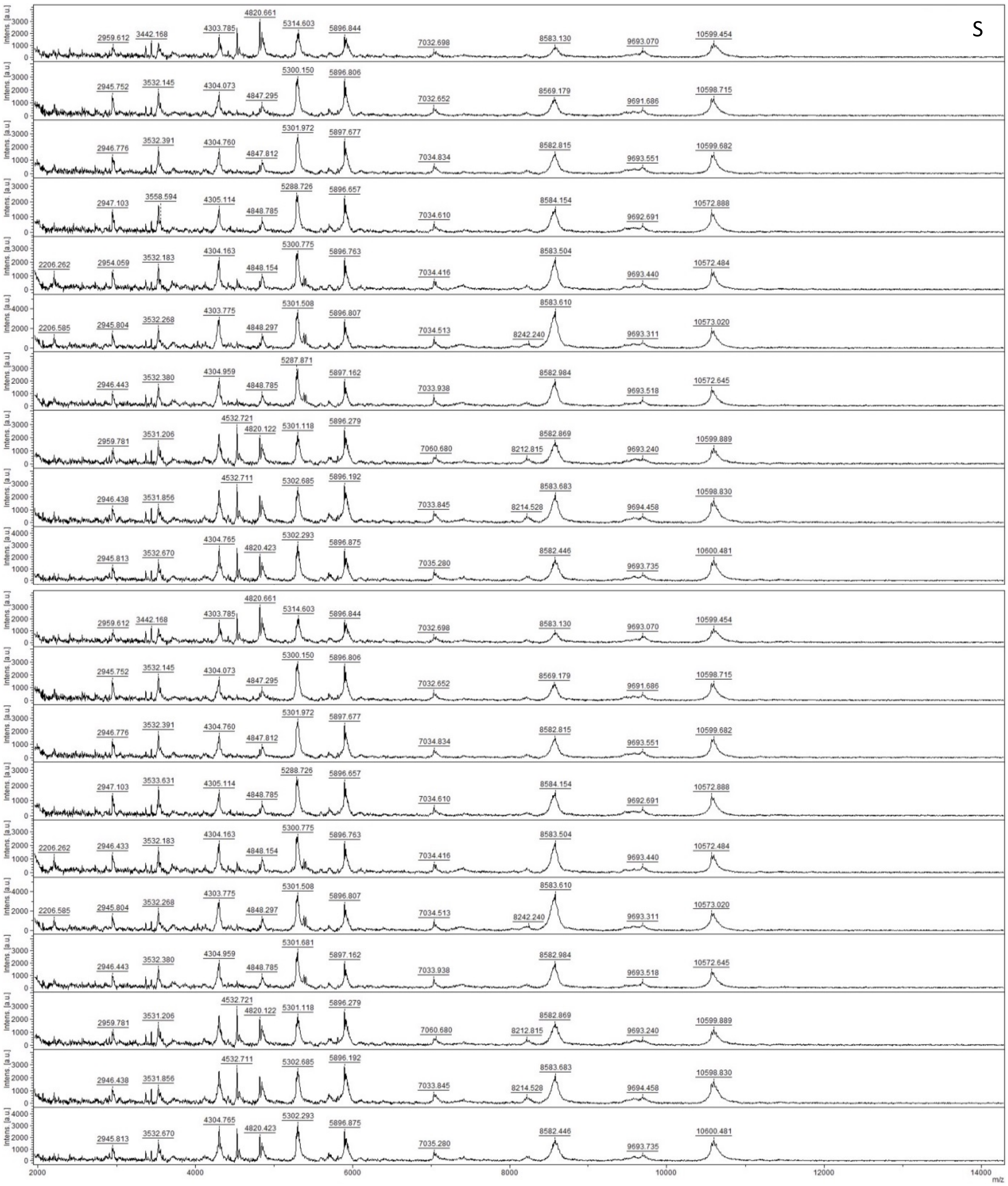

Supplementary Figure 2S. *Pervetustus simplex nc*

Figure S2. Selection of spectra from biological replicate (n=8) MALDI-TOF mass spectra of all studied species for MSP creation. Demonstrating the high degree of reproducibility within sample replicates. A. *Gigaspora rosea* BEG9*;* B. *Gigaspora margarita* BEG34; C. *Gigaspora candida* BEG17; D. *Scutellospora calospora* BEG32; E. *Rhizophagus clarus* BEG248 ; F. *Rhizophagus clarus* BEG149; G. *Rhizophagus clarus* BEG142 ; H. *Funneliformis coronatum* BEG22; I. *Funneliformis mosseae* BEG12; J. *Claroideoglomus etunicatum* BEG247; K. *Claroideoglomus claroideum* BEG31; L. *Claroideoglomus claroideum* BEG14; M. *Claroideoglomus claroideum* BEG23; N. *Scutellospora ovalis;* O. *Acaulospora saccata;* P. *Acaulospora fragilissima;* Q. *Rhizophagus neocaledonicus;* R. *Claroideoglomus etunicatum nc;* S. *Pervetustus simplex nc.*

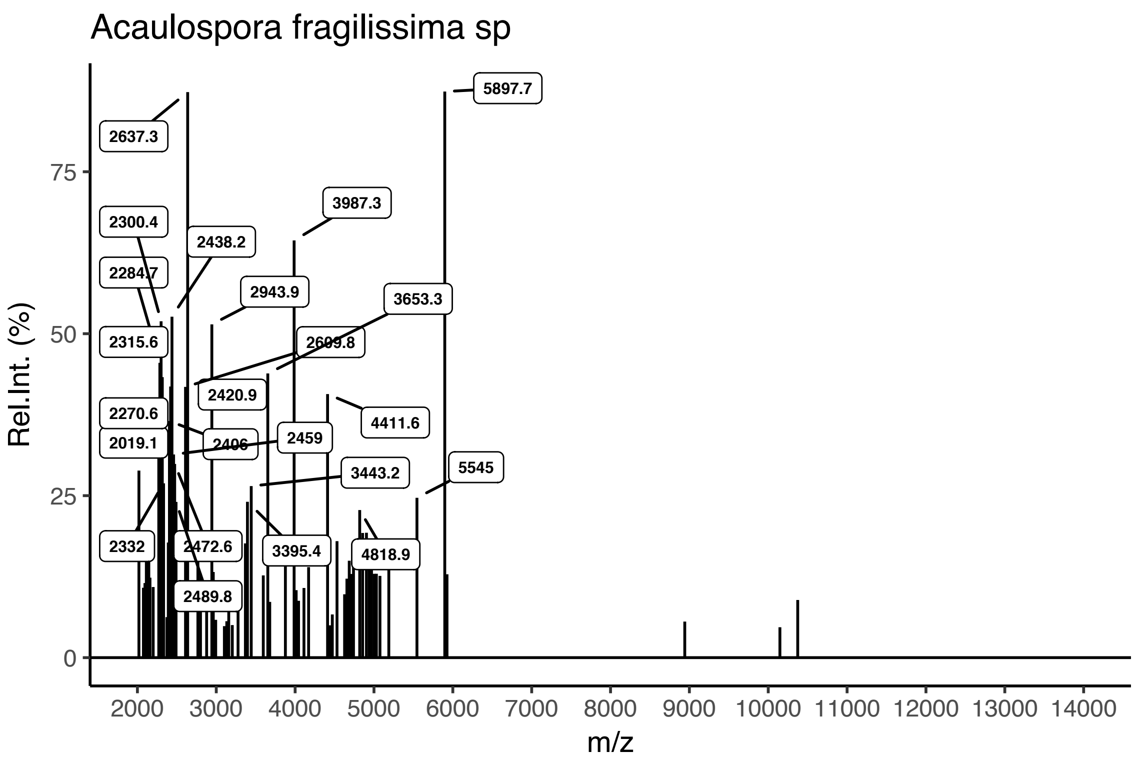
Acaulospora fragilissima sp
Rel.Int. (%)
m/z
2637.3
5897.7
3987.3
2300.4
2438.2
2284.7
2943.9
3653.3
2315.6
2609.8
2420.9
4411.6
2270.6
2406
2459
2019.1
3443.2
5545
2332
2472.6
3395.4
4818.9
2489.8

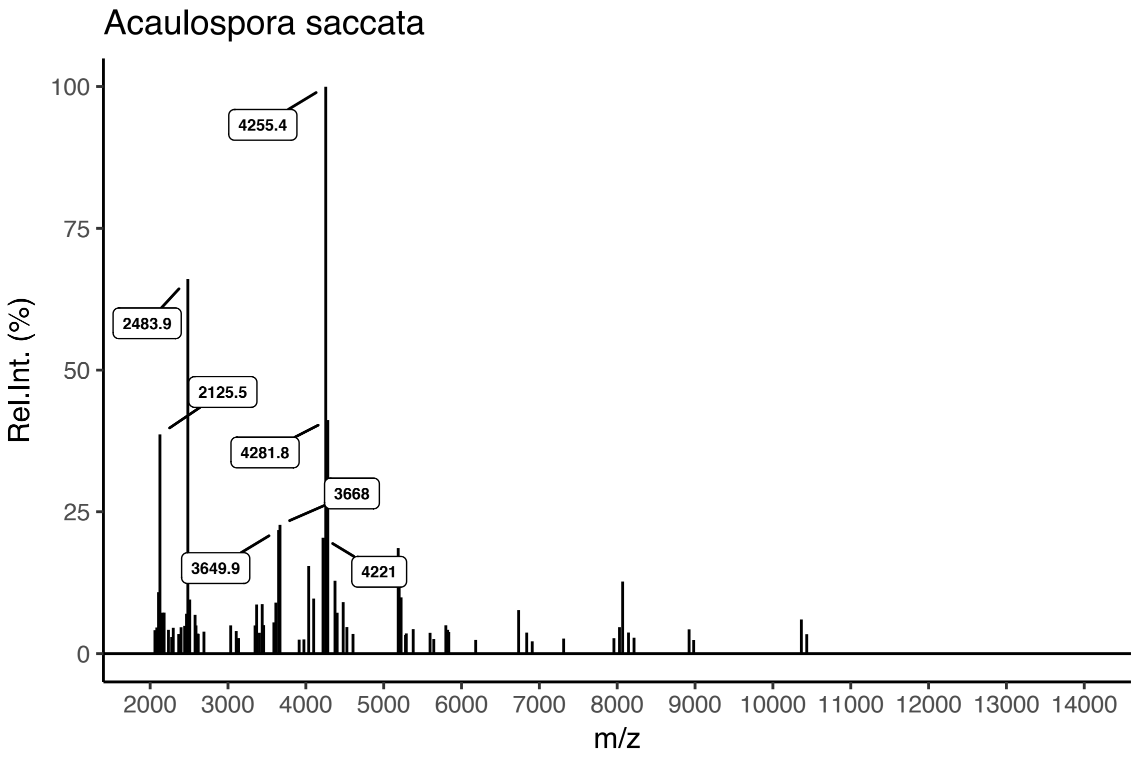
Acaulospora saccata
Rel.Int. (%)
m/z
4255.4
2483.9
2125.5
4281.8
3668
3649.9
4221

Clarodeiglomus claroideum BEG14 sp

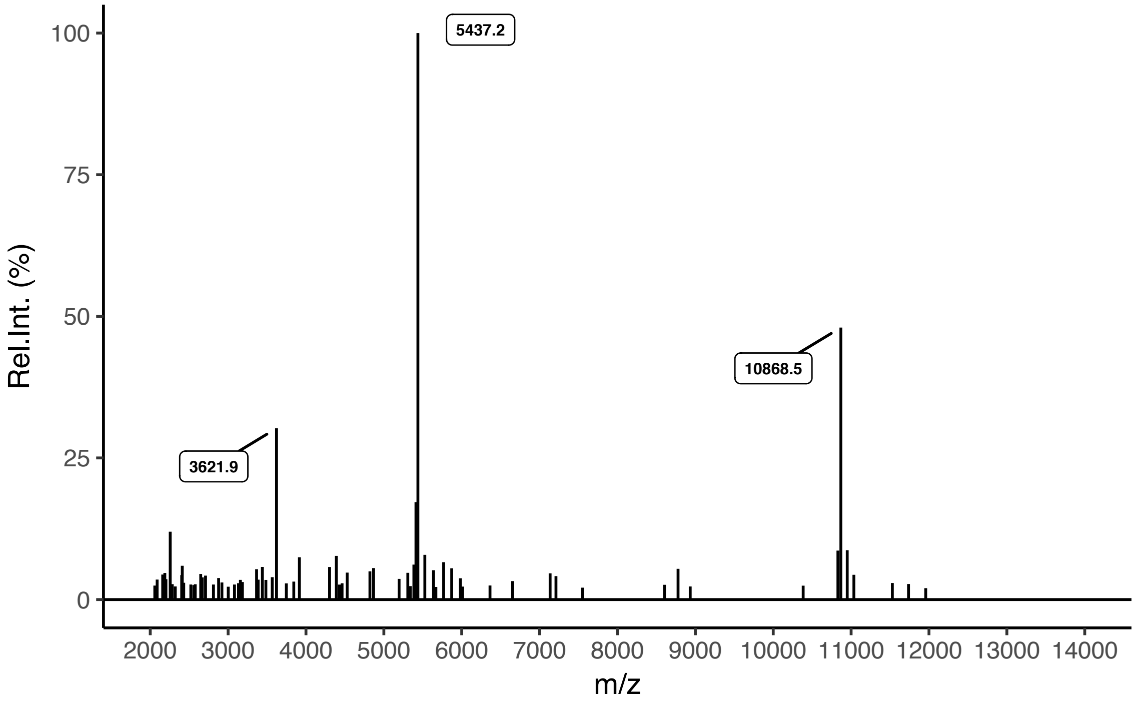

Claroideoglomus claroideum BEG23

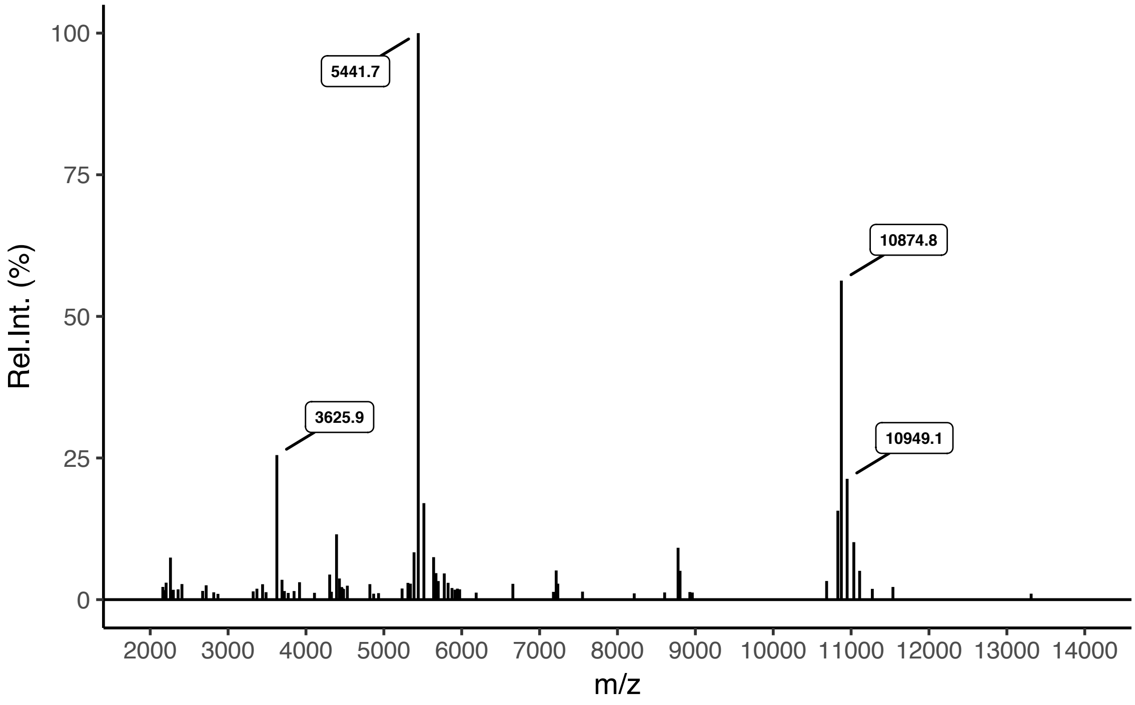

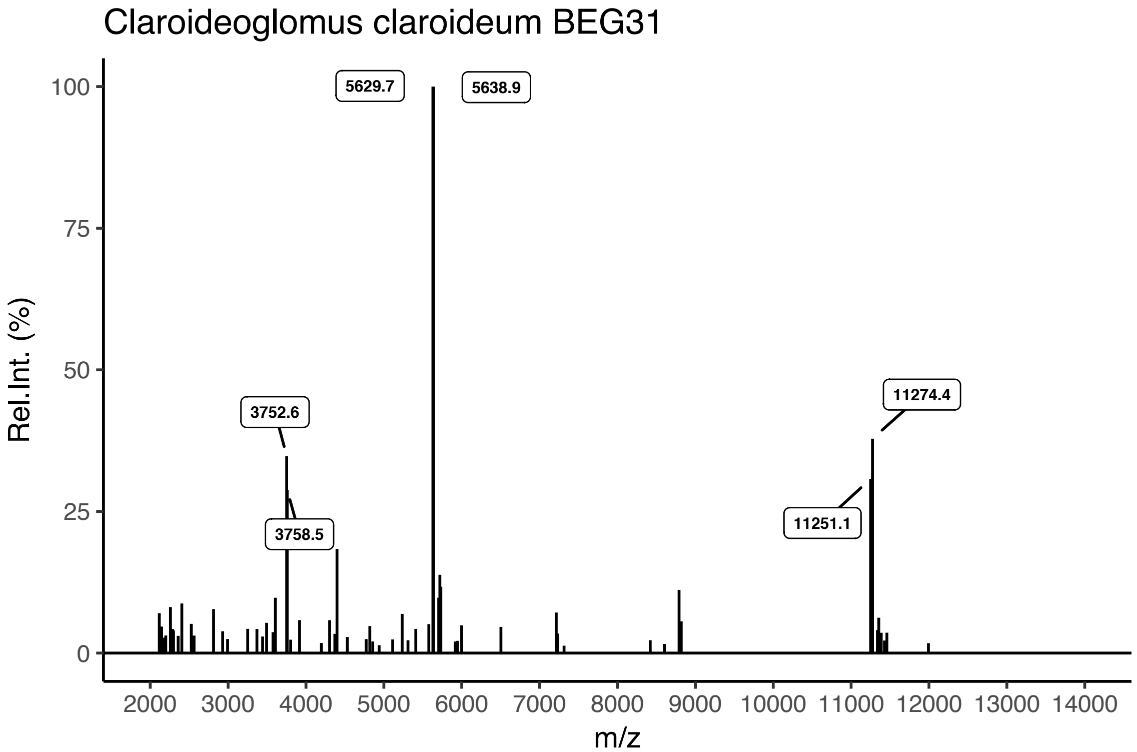
Claroideoglomus claroideum BEG31
5629.7
5638.9
3752.6
3758.5
11274.4
11251.1
Rel.Int. (%)
0
25
50
75
100
2000
3000
4000
5000
6000
7000
8000
9000
10000
11000
12000
13000
14000
m/z

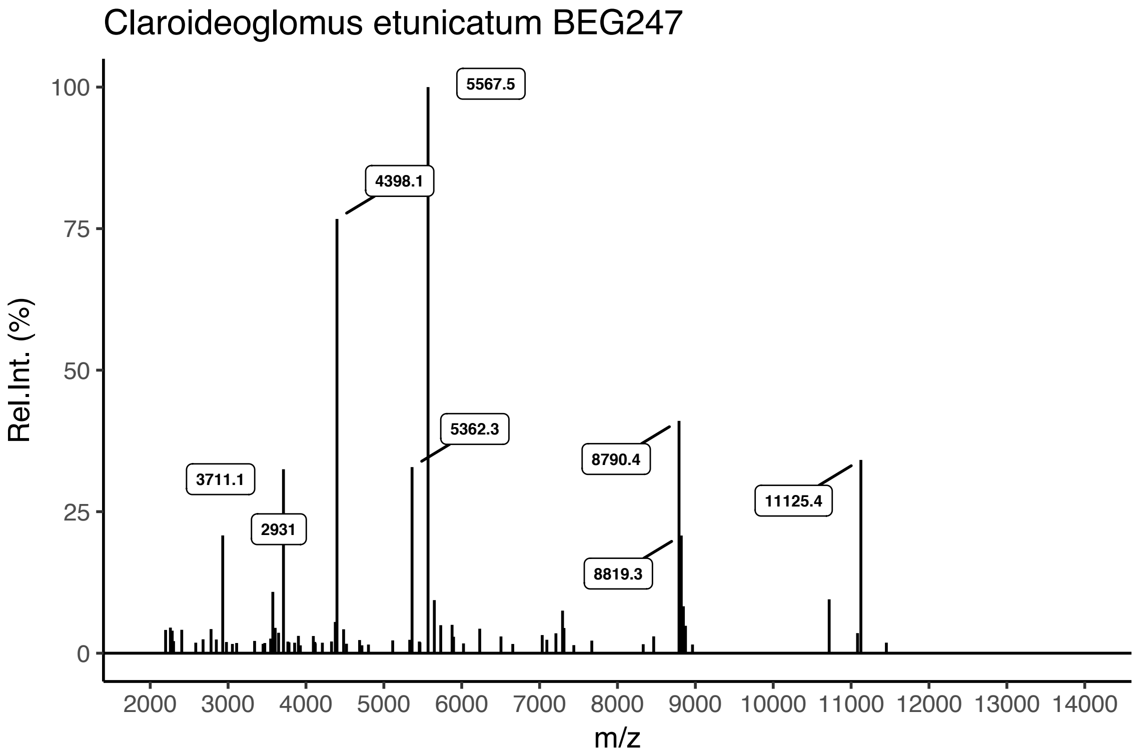
Claroideoglomus etunicatum BEG247
5567.5
4398.1
5362.3
8790.4
11125.4
3711.1
2931
8819.3
Rel.Int. (%)
0
25
50
75
100
2000
3000
4000
5000
6000
7000
8000
9000
10000
11000
12000
13000
14000
m/z

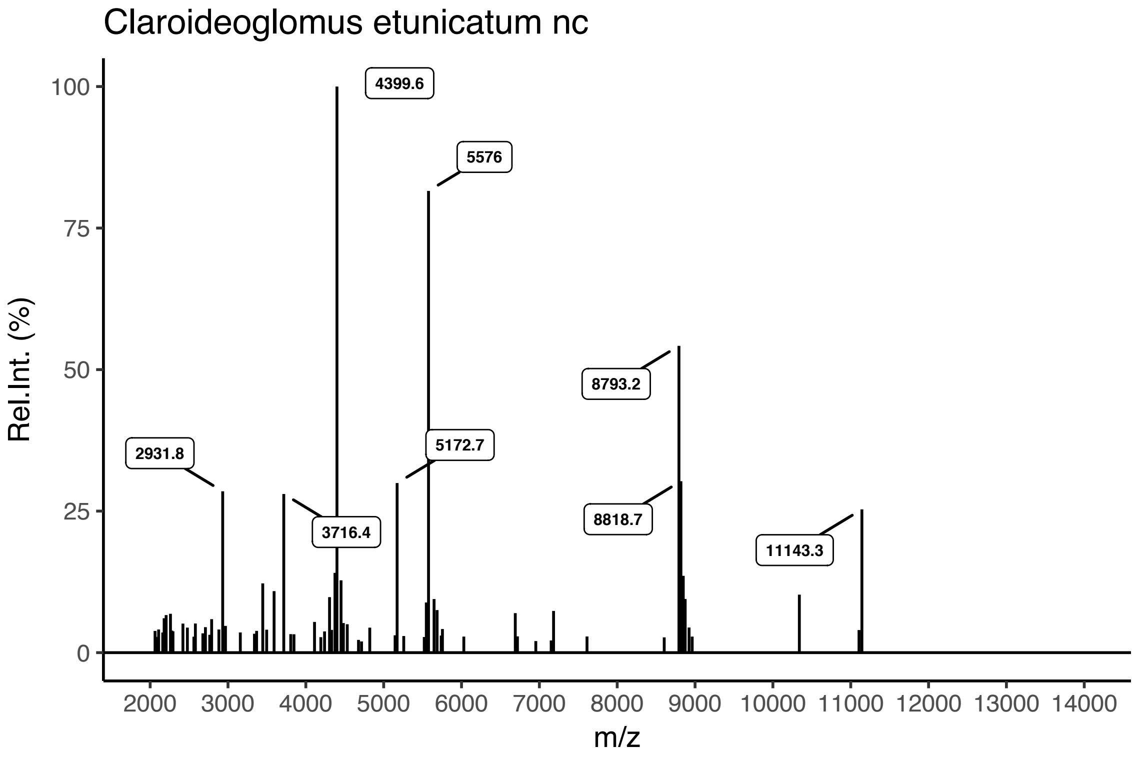
Claroideoglomus etunicatum nc
Rel.Int. (%)
m/z
4399.6
5576
8793.2
2931.8
5172.7
3716.4
8818.7
11143.3

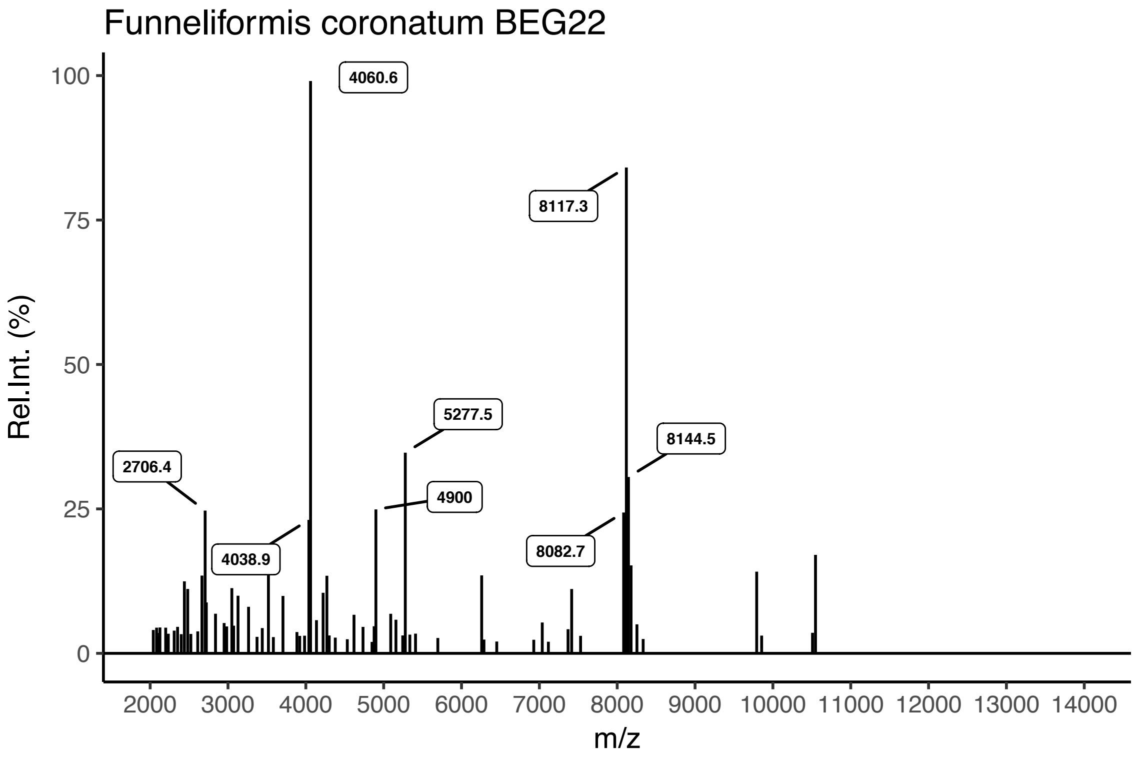
Funneliformis coronatum BEG22
Rel.Int. (%)
m/z
4060.6
8117.3
5277.5
8144.5
2706.4
4900
4038.9
8082.7

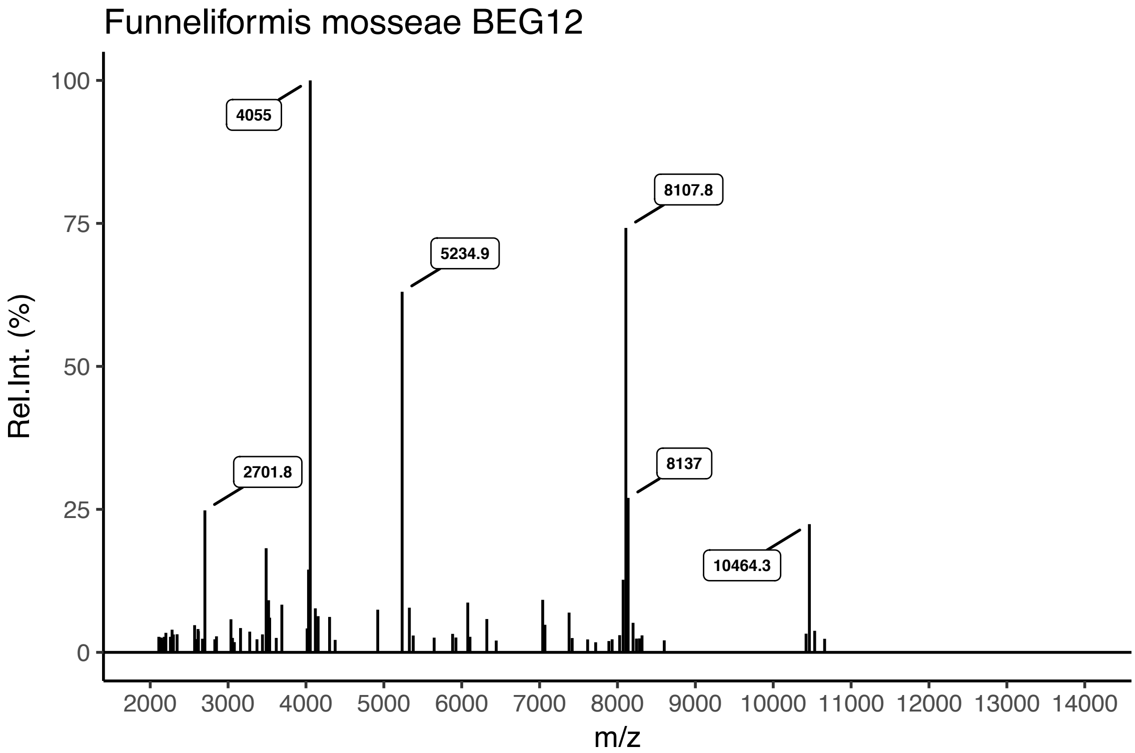
Funneliformis mosseae BEG12
Rel.Int. (%)
m/z
4055
5234.9
8107.8
2701.8
8137
10464.3
0
25
50
75
100
2000 3000 4000 5000 6000 7000 8000 9000 10000 11000 12000 13000 14000

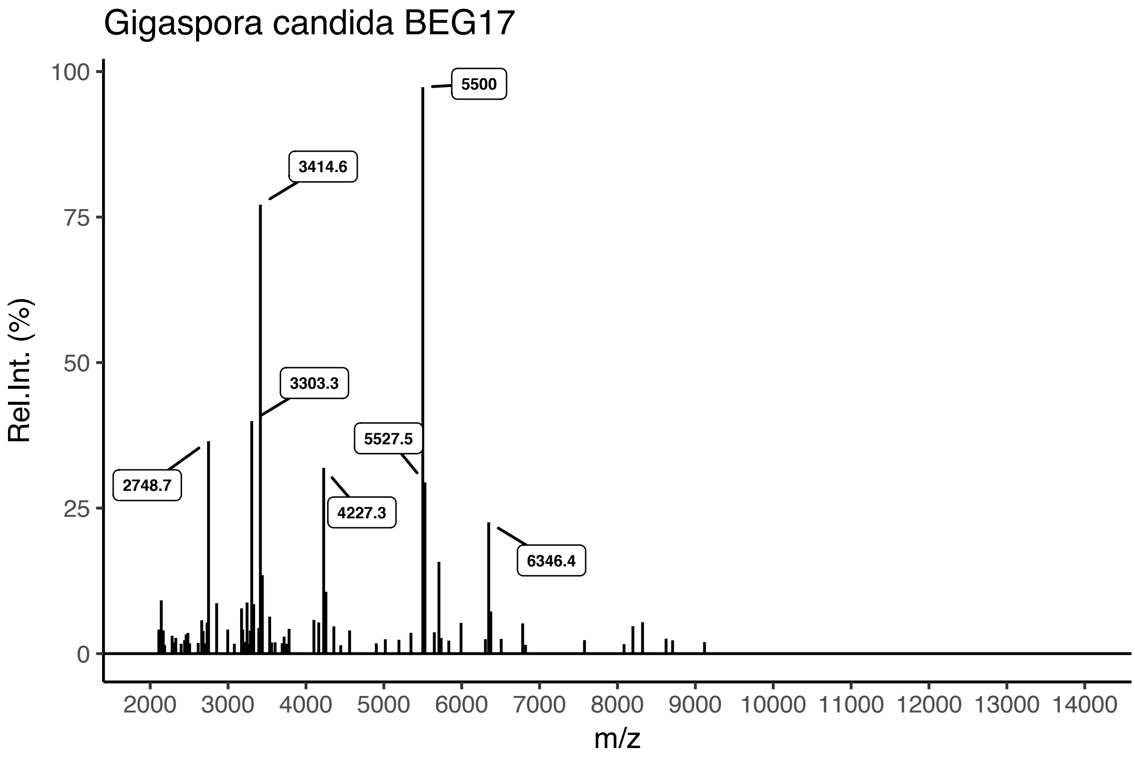
Gigaspora candida BEG17
Rel.Int. (%)
m/z
5500
3414.6
3303.3
2748.7
5527.5
4227.3
6346.4
0
25
50
75
100
2000 3000 4000 5000 6000 7000 8000 9000 10000 11000 12000 13000 14000

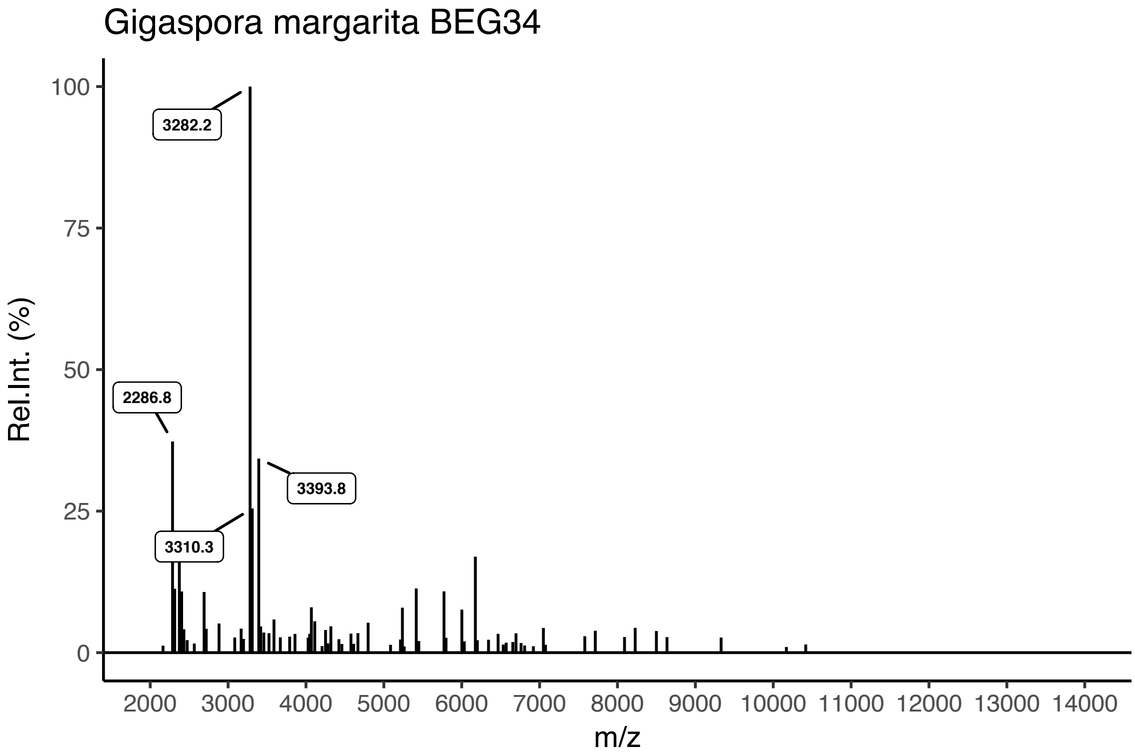
Gigaspora margarita BEG34
Rel.Int. (%)
100
75
50
25
0
3282.2
2286.8
3393.8
3310.3
2000
3000
4000
5000
6000
7000
8000
9000
10000
11000
12000
13000
14000
m/z

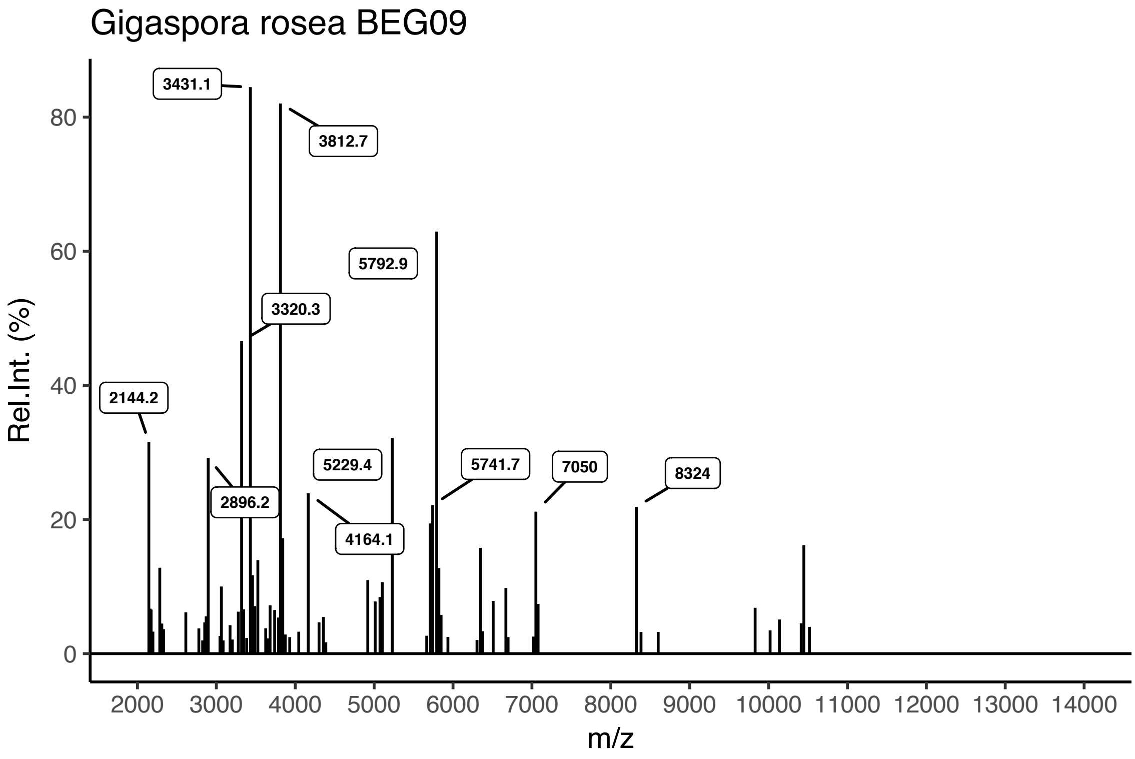
Gigaspora rosea BEG09
Rel.Int. (%)
80
60
40
20
0
3431.1
3812.7
5792.9
3320.3
2144.2
5229.4
5741.7
7050
8324
2896.2
4164.1
2000
3000
4000
5000
6000
7000
8000
9000
10000
11000
12000
13000
14000
m/z

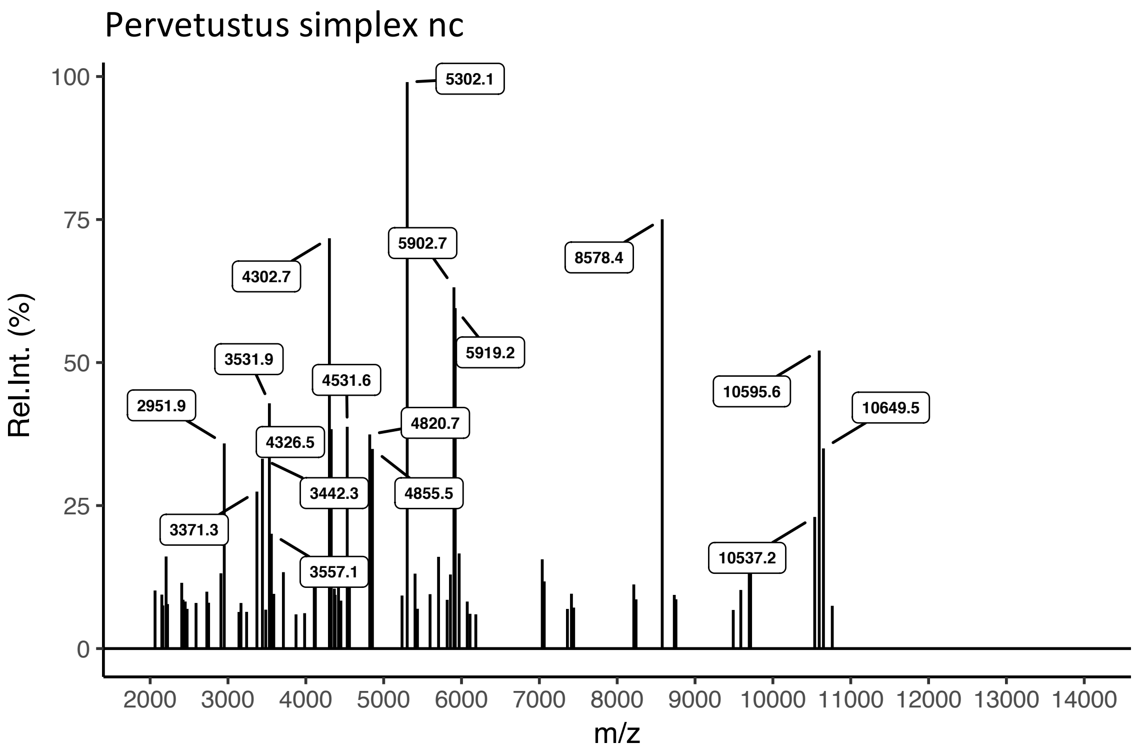
Pervetustus simplex nc
Rel.Int. (%)
m/z
5302.1
4302.7
5902.7
8578.4
5919.2
3531.9
4531.6
2951.9
10595.6
10649.5
4820.7
4326.5
3442.3
4855.5
3371.3
10537.2
3557.1

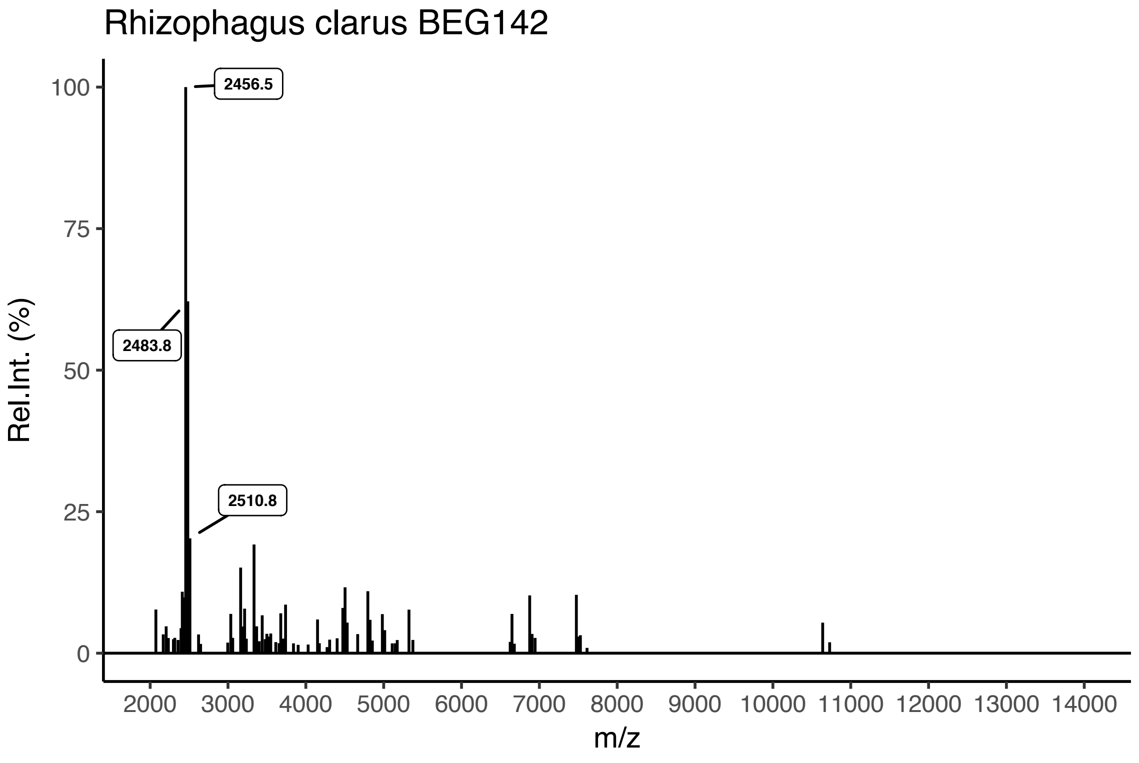
Rhizophagus clarus BEG142
Rel.Int. (%)
m/z
2456.5
2483.8
2510.8

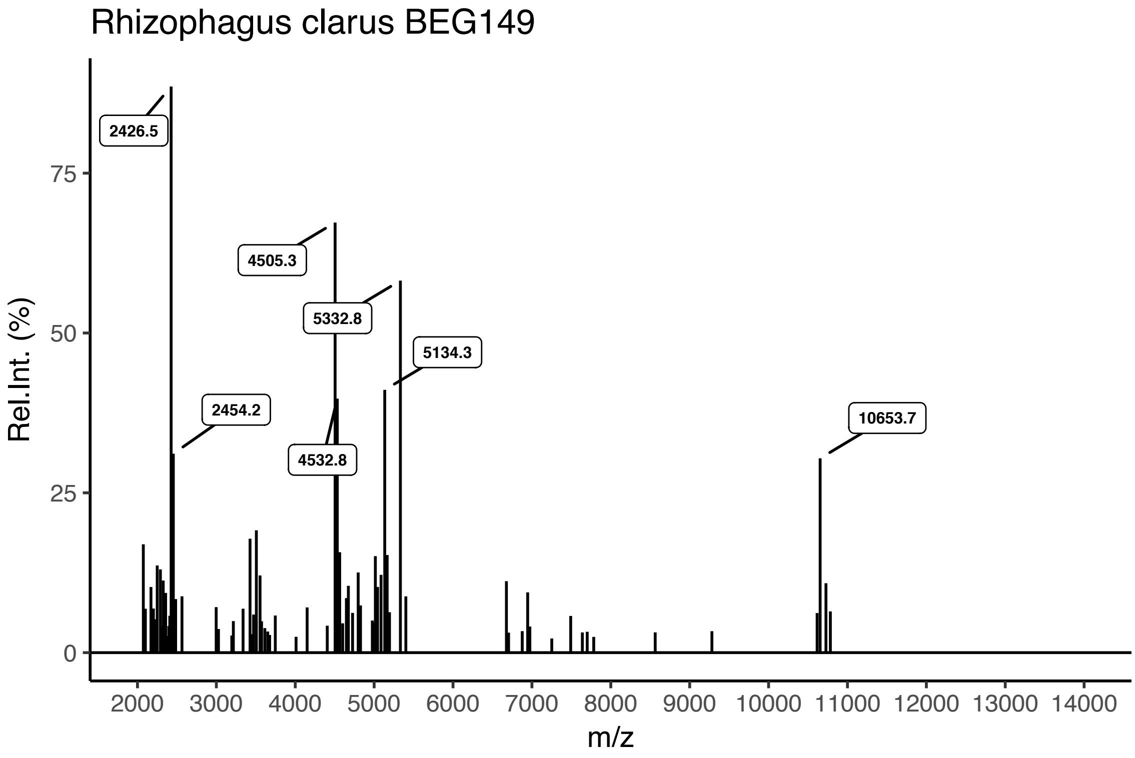
Rhizophagus clarus BEG149
Rel.Int. (%)
m/z
2426.5
4505.3
5332.8
5134.3
2454.2
4532.8
10653.7

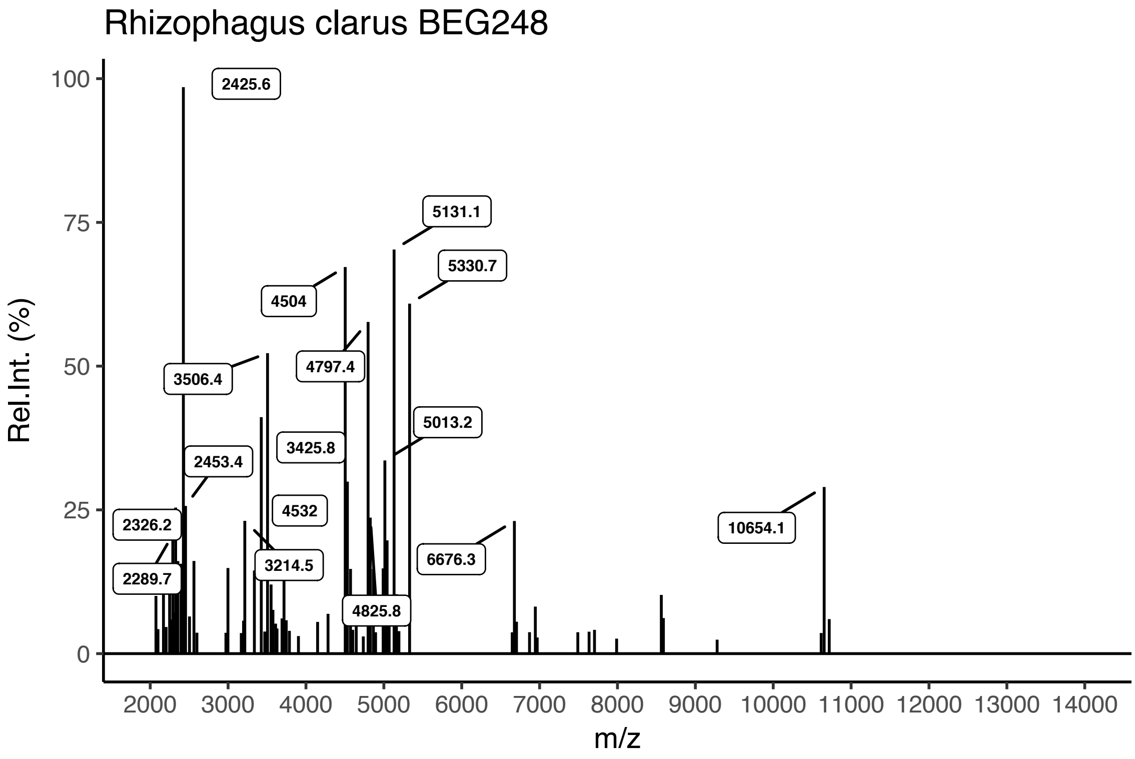
Rhizophagus clarus BEG248
Rel.Int. (%)
m/z
2425.6
5131.1
5330.7
4504
3506.4
4797.4
5013.2
3425.8
2453.4
4532
2326.2
3214.5
2289.7
6676.3
10654.1
4825.8

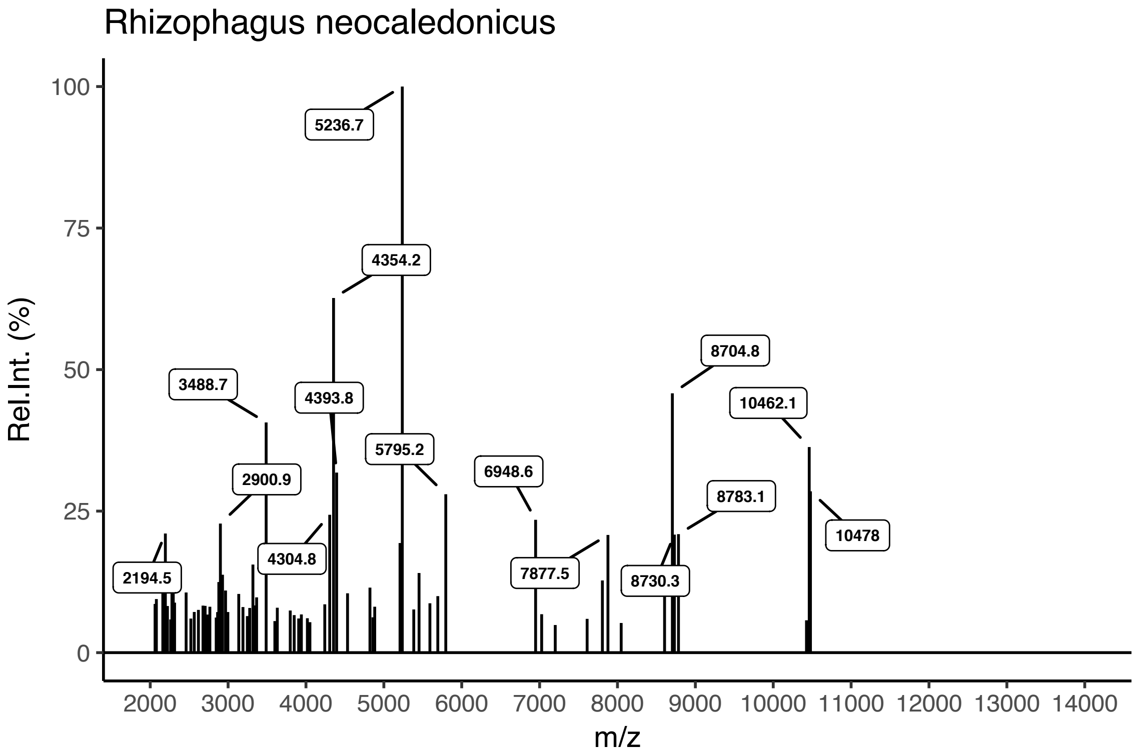

Rhizophagus neocaledonicus
Rel.Int. (%)
0
25
50
75
100
5236.7
4354.2
8704.8
3488.7
4393.8
10462.1
5795.2
2900.9
6948.6
8783.1
10478
4304.8
7877.5
2194.5
8730.3
2000
3000
4000
5000
6000
7000
8000
9000
10000
11000
12000
13000
14000
m/z

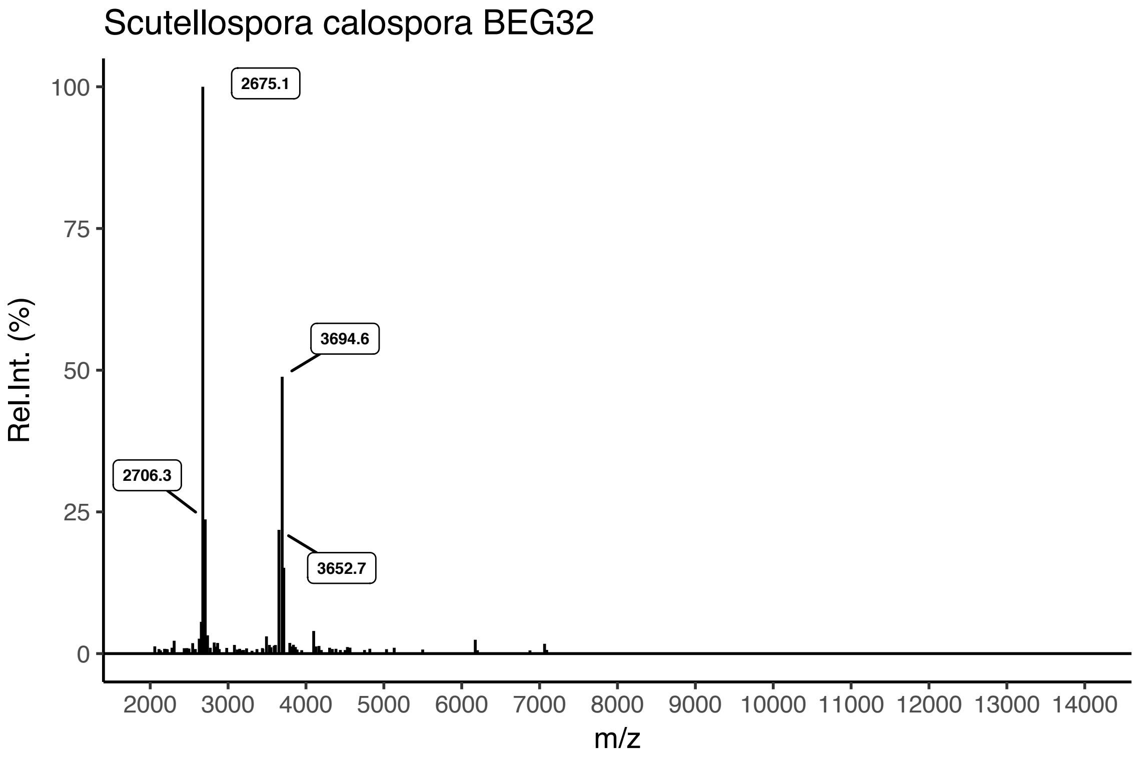

Scutellospora calospora BEG32
Rel.Int. (%)
0
25
50
75
100
2675.1
3694.6
2706.3
3652.7
2000
3000
4000
5000
6000
7000
8000
9000
10000
11000
12000
13000
14000
m/z

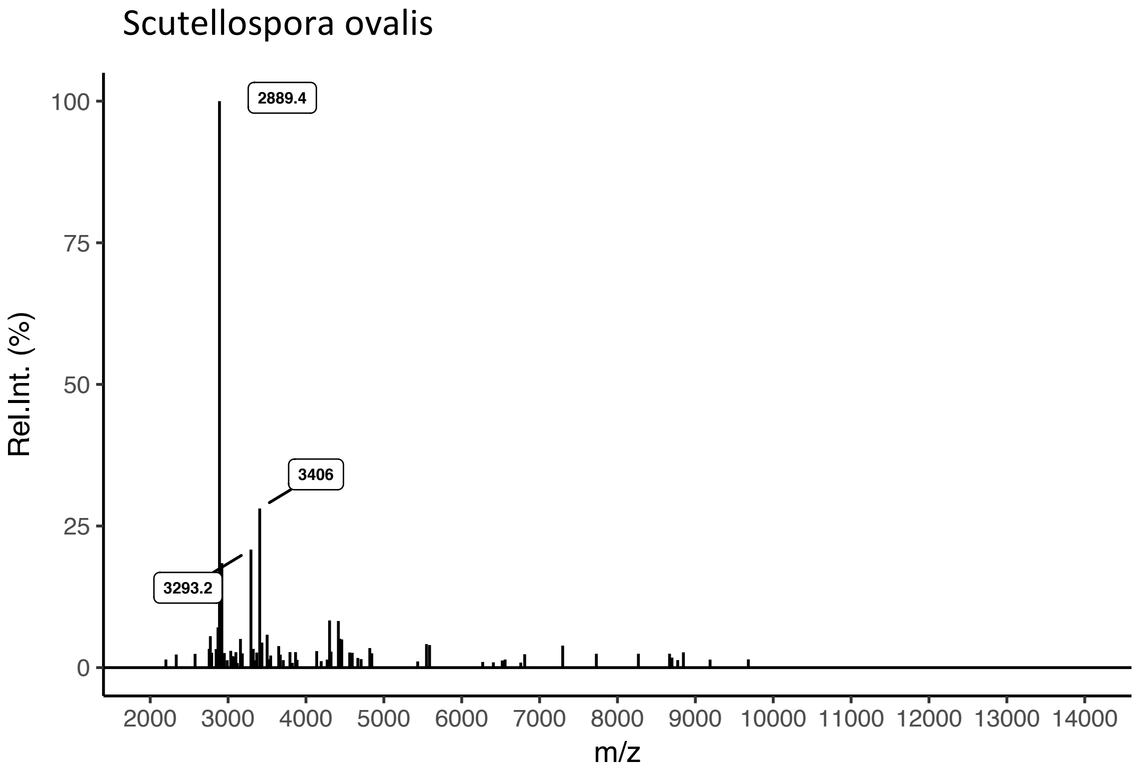

Supplementary Figure 3. MALDI-TOF-MS main spectrum data export of all studied species for MSP centroid representation under R software (version 3.3.2[30]).

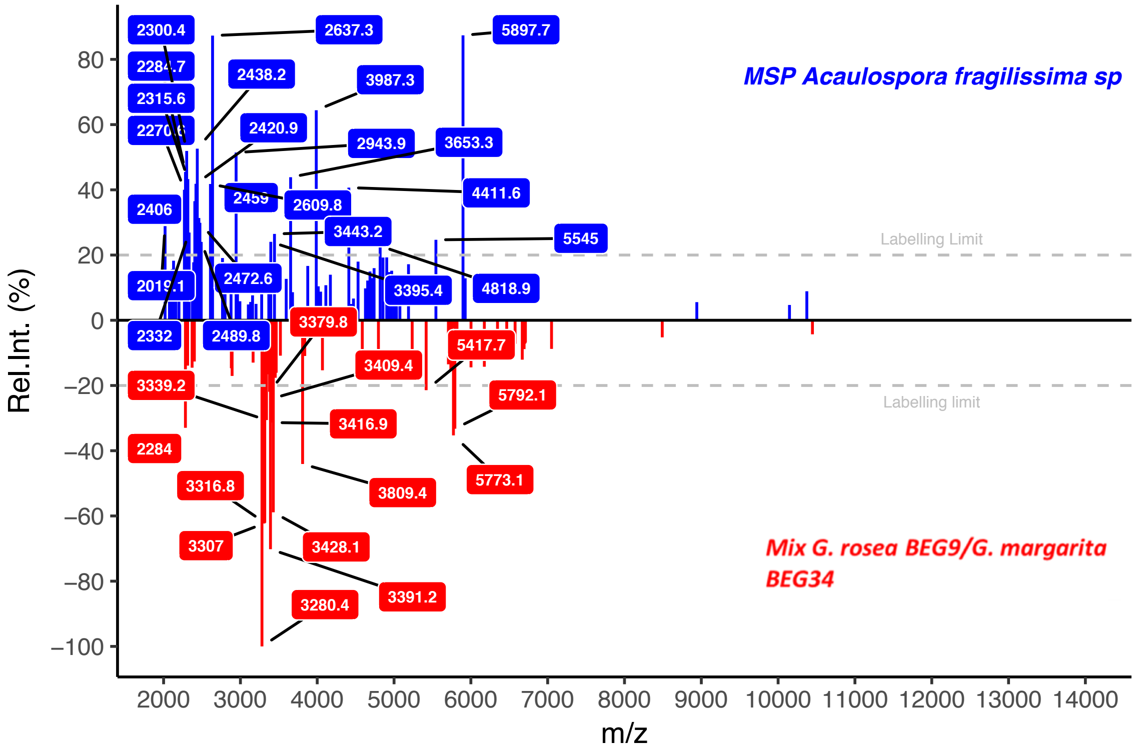
MSP Acaulospora fragilissima sp
Mix G. rosea BEG9/G. margarita BEG34
Labelling Limit
Labelling limit
Rel.Int. (%)
m/z
2300.4
2284.7
2315.6
2406
2019.1
2332
2637.3
2438.2
2420.9
2459
2472.6
2489.8
2609.8
2943.9
3987.3
3653.3
3443.2
3395.4
4411.6
5545
4818.9
5897.7
3379.8
3409.4
3339.2
3416.9
2284
3316.8
3809.4
3307
3428.1
3391.2
3280.4
5417.7
5792.1
5773.1

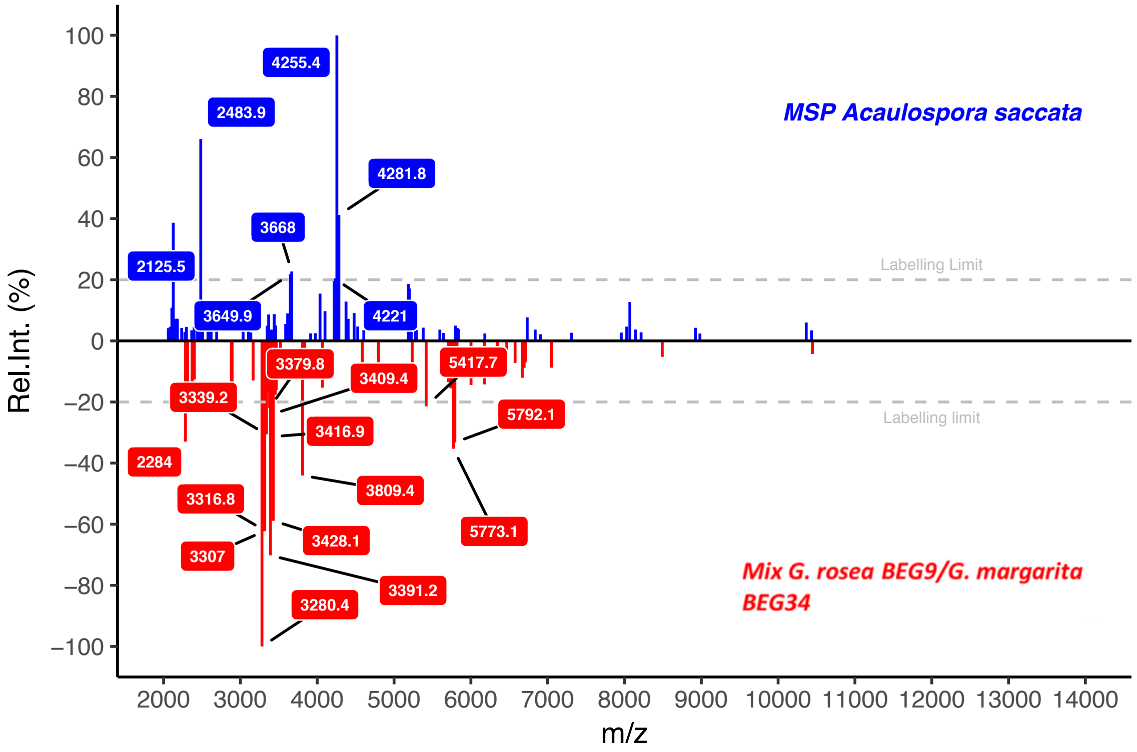
MSP Acaulospora saccata
Mix G. rosea BEG9/G. margarita BEG34
Labelling Limit
Labelling limit
Rel.Int. (%)
m/z
4255.4
2483.9
4281.8
3668
2125.5
3649.9
4221
3379.8
3409.4
5417.7
3339.2
3416.9
5792.1
2284
3809.4
3316.8
5773.1
3428.1
3307
3391.2
3280.4

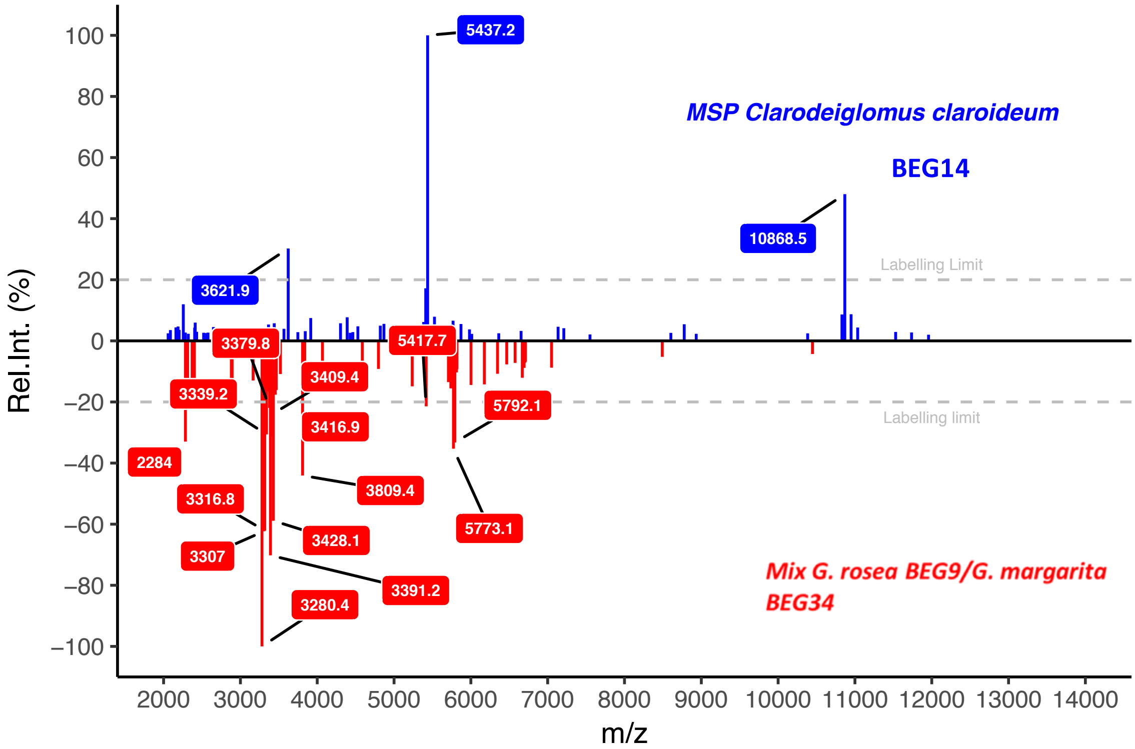
MSP Clarodeiglomus claroideum
BEG14
Mix G. rosea BEG9/G. margarita BEG34
Rel.Int. (%)
m/z
Labelling Limit
Labelling limit
5437.2
10868.5
3621.9
3379.8
5417.7
3409.4
3339.2
3416.9
5792.1
2284
3809.4
3316.8
5773.1
3428.1
3307
3391.2
3280.4
100
80
60
40
20
0
−20
−40
−60
−80
−100
2000 3000 4000 5000 6000 7000 8000 9000 10000 11000 12000 13000 14000

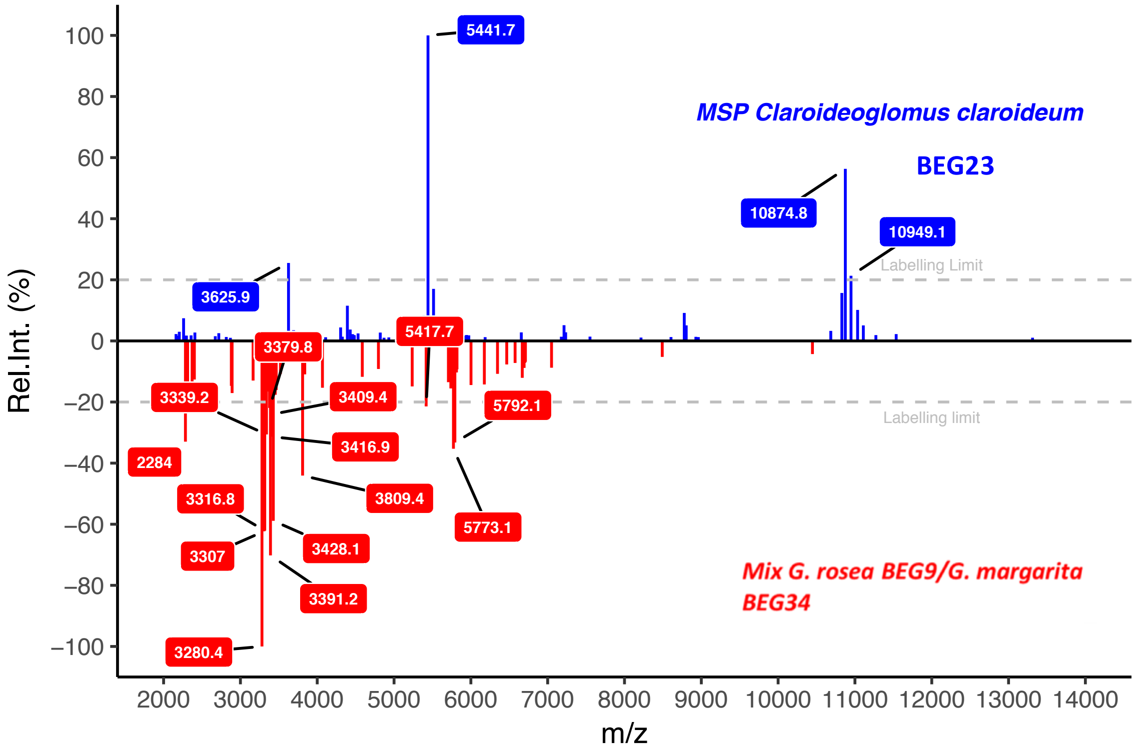
MSP Claroideoglomus claroideum
BEG23
Mix G. rosea BEG9/G. margarita BEG34
Rel.Int. (%)
m/z
Labelling Limit
Labelling limit
5441.7
10874.8
10949.1
3625.9
3379.8
5417.7
3339.2
3409.4
5792.1
3416.9
2284
3809.4
3316.8
5773.1
3428.1
3307
3391.2
3280.4
100
80
60
40
20
0
−20
−40
−60
−80
−100
2000 3000 4000 5000 6000 7000 8000 9000 10000 11000 12000 13000 14000

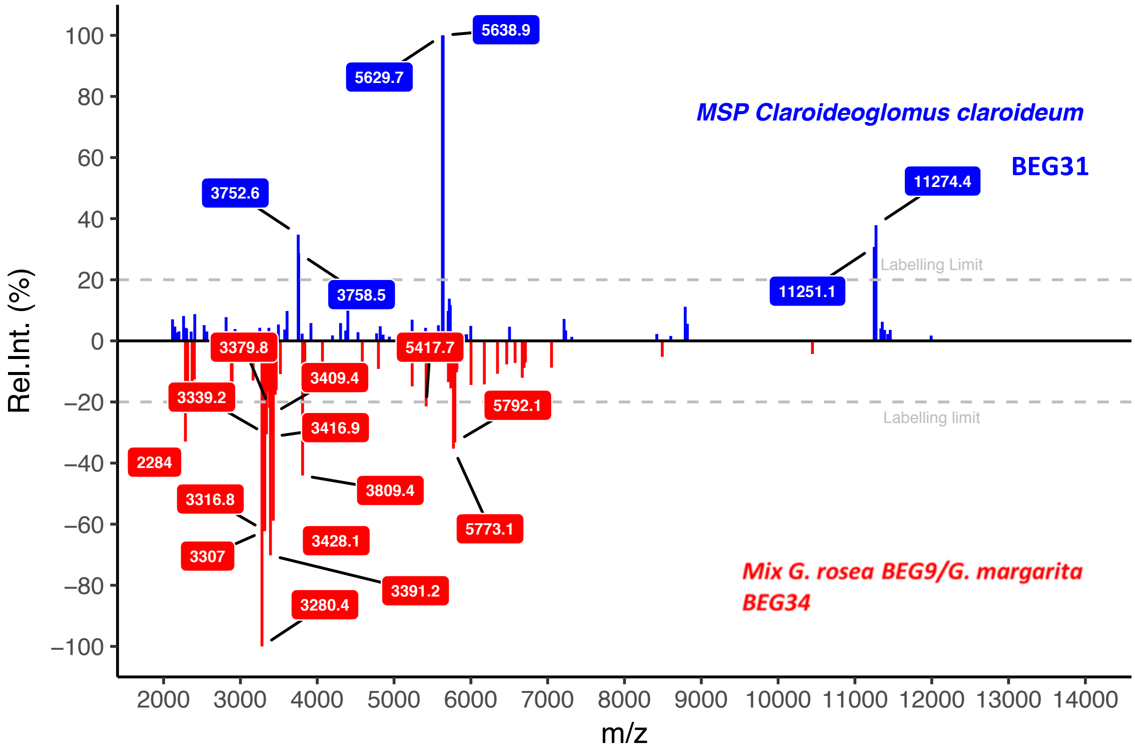
MSP Claroideoglomus claroideum
BEG31
Mix G. rosea BEG9/G. margarita
BEG34
Rel.Int. (%)
m/z
Labelling Limit
Labelling limit
5638.9
5629.7
3752.6
3758.5
11274.4
11251.1
3379.8
5417.7
3409.4
3339.2
5792.1
3416.9
2284
3809.4
3316.8
5773.1
3428.1
3307
3391.2
3280.4
100
80
60
40
20
0
−20
−40
−60
−80
−100
2000 3000 4000 5000 6000 7000 8000 9000 10000 11000 12000 13000 14000

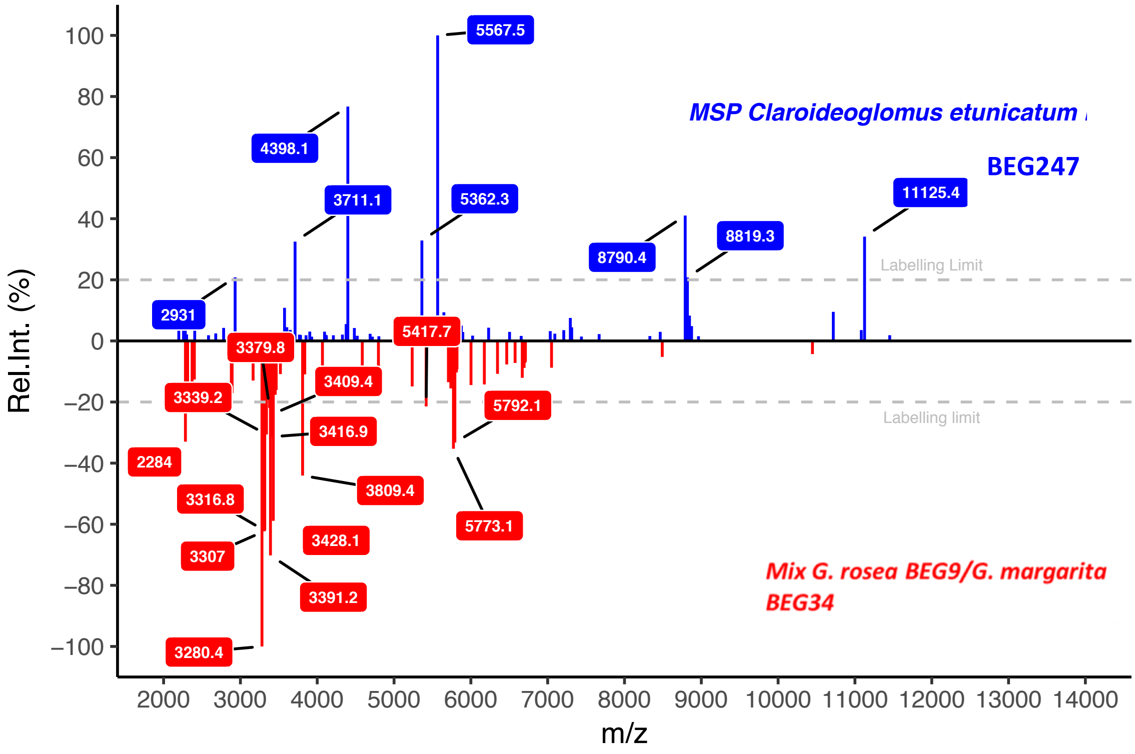
MSP Claroideoglomus etunicatum
BEG247
Mix G. rosea BEG9/G. margarita
BEG34
Rel.Int. (%)
m/z
Labelling Limit
Labelling limit
5567.5
4398.1
3711.1
5362.3
11125.4
8819.3
8790.4
2931
3379.8
5417.7
3409.4
3339.2
5792.1
3416.9
2284
3809.4
3316.8
5773.1
3428.1
3307
3391.2
3280.4
100
80
60
40
20
0
−20
−40
−60
−80
−100
2000 3000 4000 5000 6000 7000 8000 9000 10000 11000 12000 13000 14000

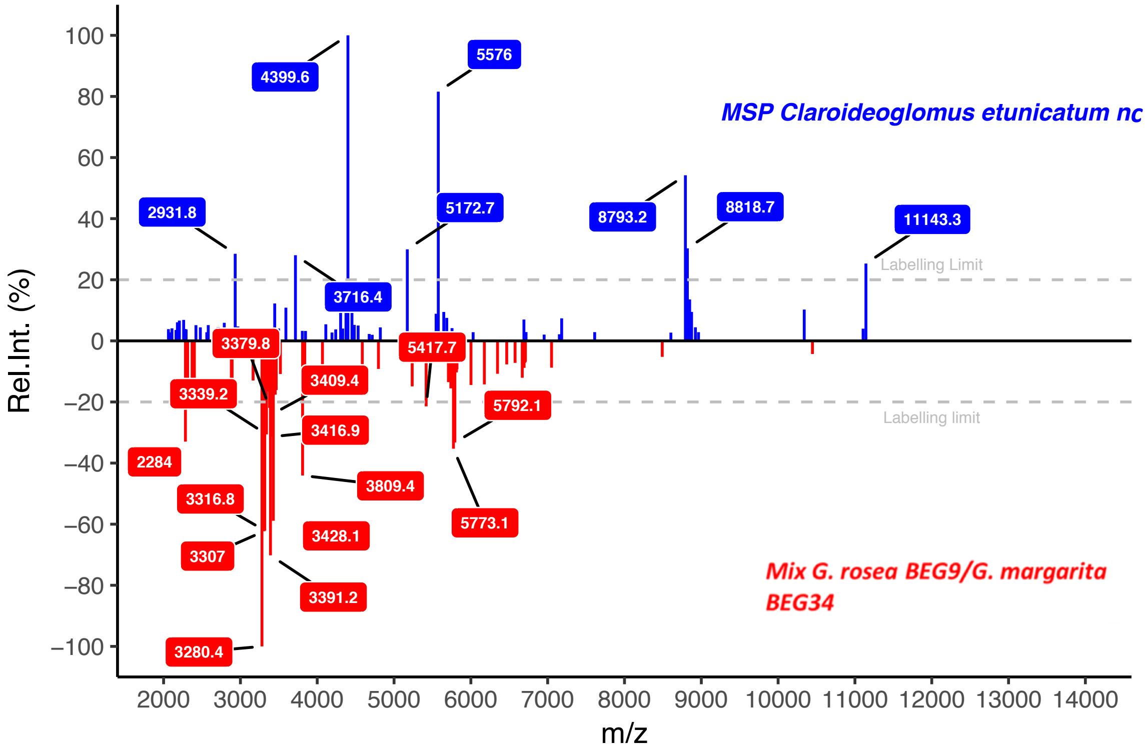
MSP Claroideoglomus etunicatum nc
Mix G. rosea BEG9/G. margarita BEG34
Rel.Int. (%)
m/z
Labelling Limit
Labelling limit
4399.6
5576
2931.8
5172.7
8793.2
8818.7
11143.3
3716.4
3379.8
5417.7
3339.2
3409.4
5792.1
3416.9
2284
3809.4
3316.8
5773.1
3428.1
3307
3391.2
3280.4

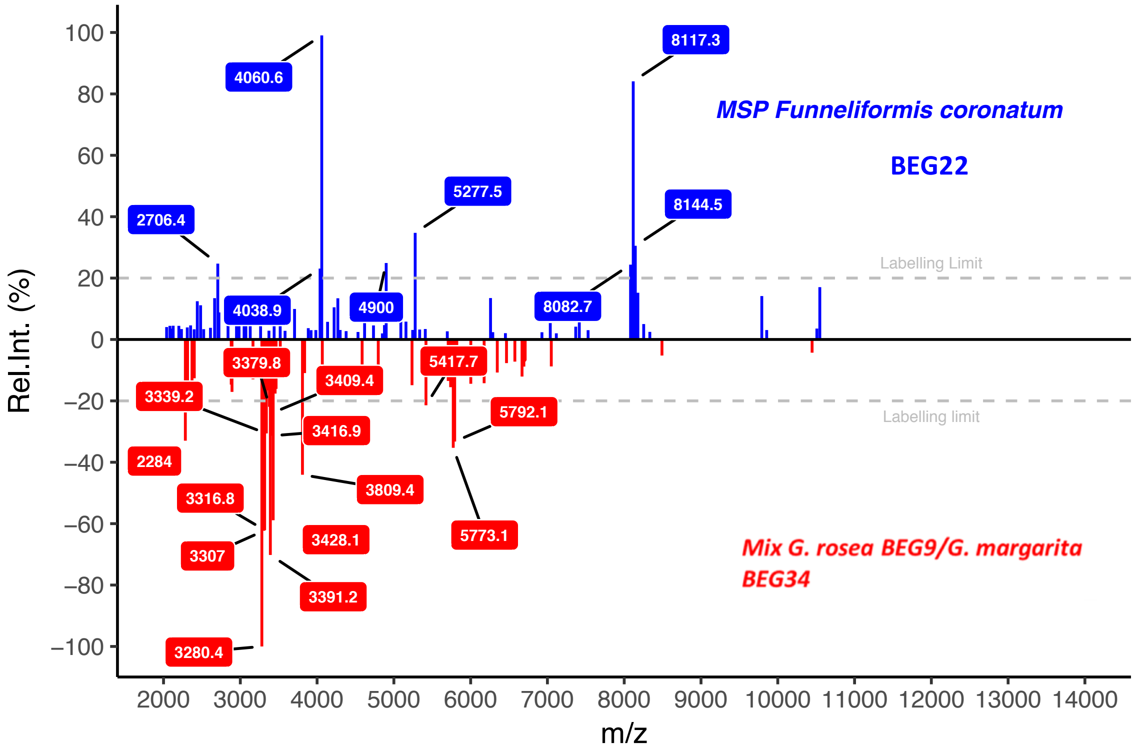
MSP Funneliformis coronatum
BEG22
Mix G. rosea BEG9/G. margarita BEG34
Rel.Int. (%)
m/z
Labelling Limit
Labelling limit
4060.6
8117.3
5277.5
2706.4
8144.5
4038.9
4900
8082.7
3379.8
5417.7
3339.2
3409.4
5792.1
3416.9
2284
3809.4
3316.8
5773.1
3428.1
3307
3391.2
3280.4

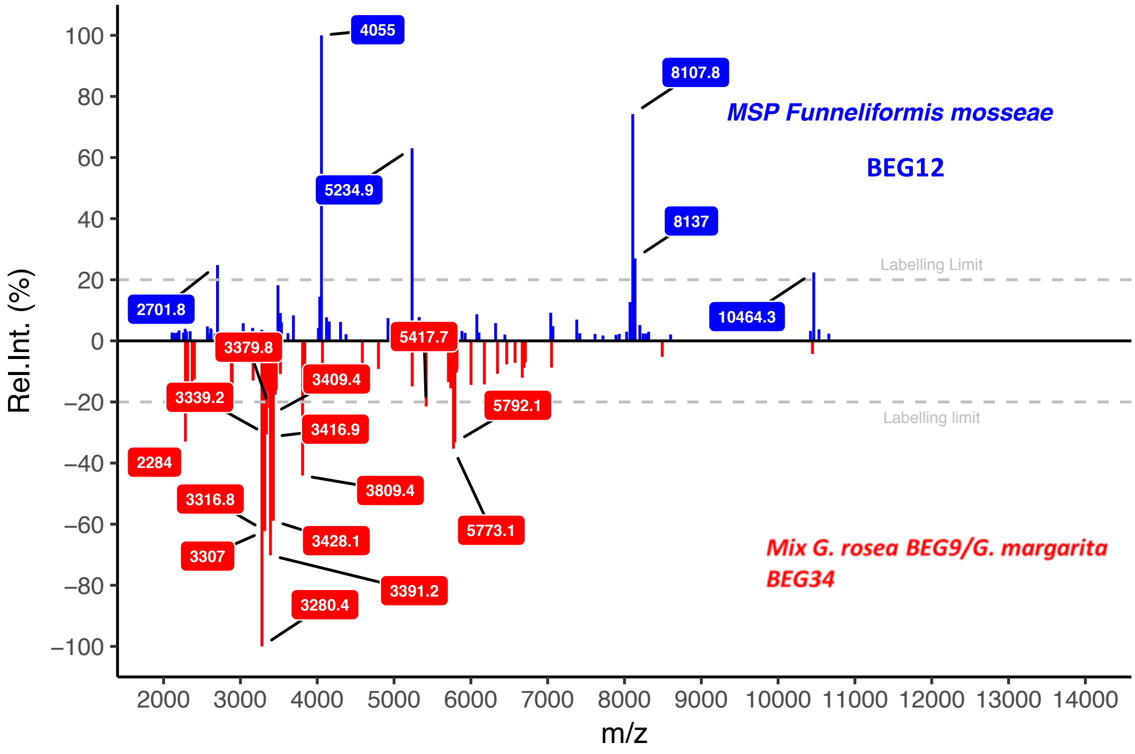
MSP Funneliformis mosseae
BEG12
Mix G. rosea BEG9/G. margarita BEG34
Labelling Limit
Labelling limit
4055
8107.8
5234.9
8137
2701.8
10464.3
3379.8
5417.7
3409.4
3339.2
5792.1
3416.9
2284
3809.4
3316.8
5773.1
3428.1
3307
3391.2
3280.4
Rel.Int. (%)
m/z

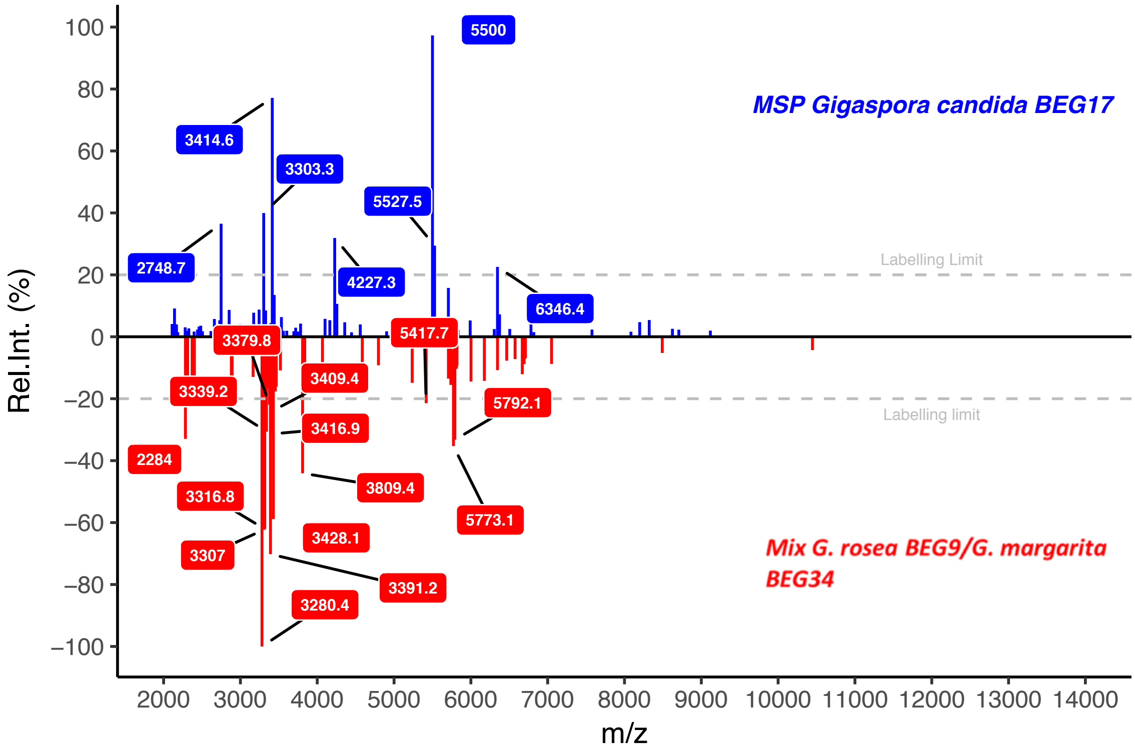
MSP Gigaspora candida BEG17
Mix G. rosea BEG9/G. margarita BEG34
Labelling Limit
Labelling limit
5500
3414.6
3303.3
5527.5
2748.7
4227.3
6346.4
3379.8
5417.7
3409.4
3339.2
5792.1
3416.9
2284
3809.4
3316.8
5773.1
3428.1
3307
3391.2
3280.4
Rel.Int. (%)
m/z

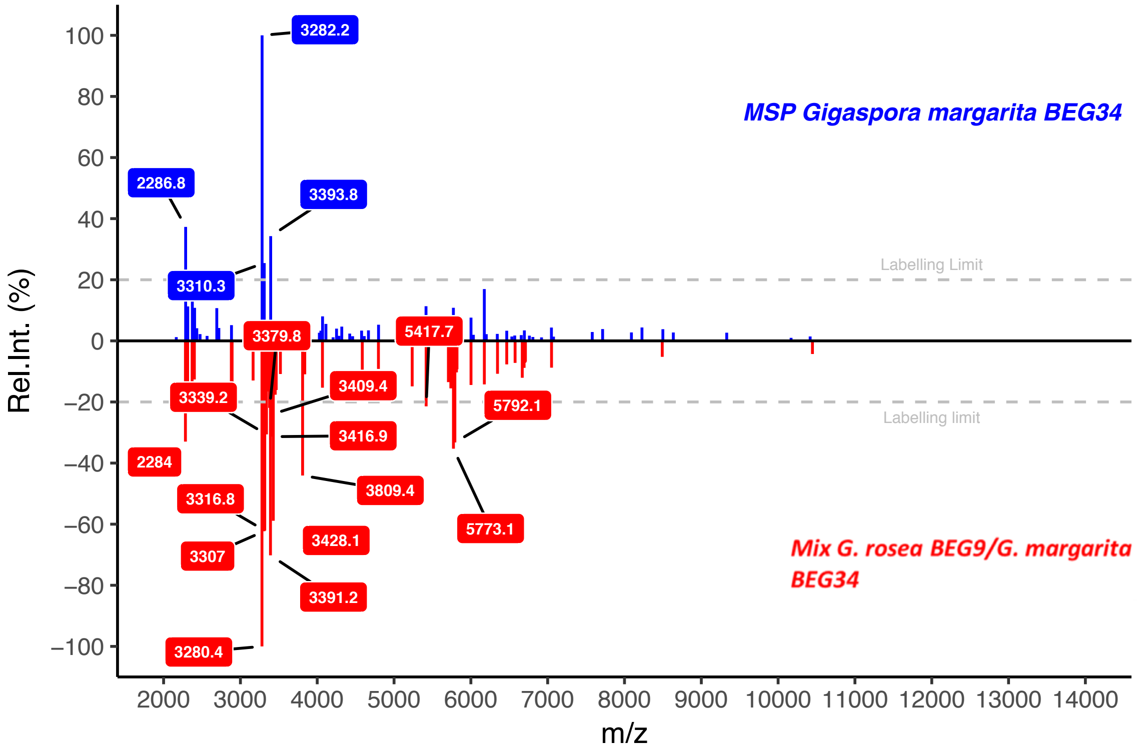

MSP Gigaspora margarita BEG34
Mix G. rosea BEG9/G. margarita BEG34
Labelling Limit
Labelling limit
Rel.Int. (%)
m/z
3282.2
2286.8
3393.8
3310.3
3379.8
5417.7
3339.2
3409.4
5792.1
3416.9
2284
3809.4
3316.8
5773.1
3307
3428.1
3391.2
3280.4

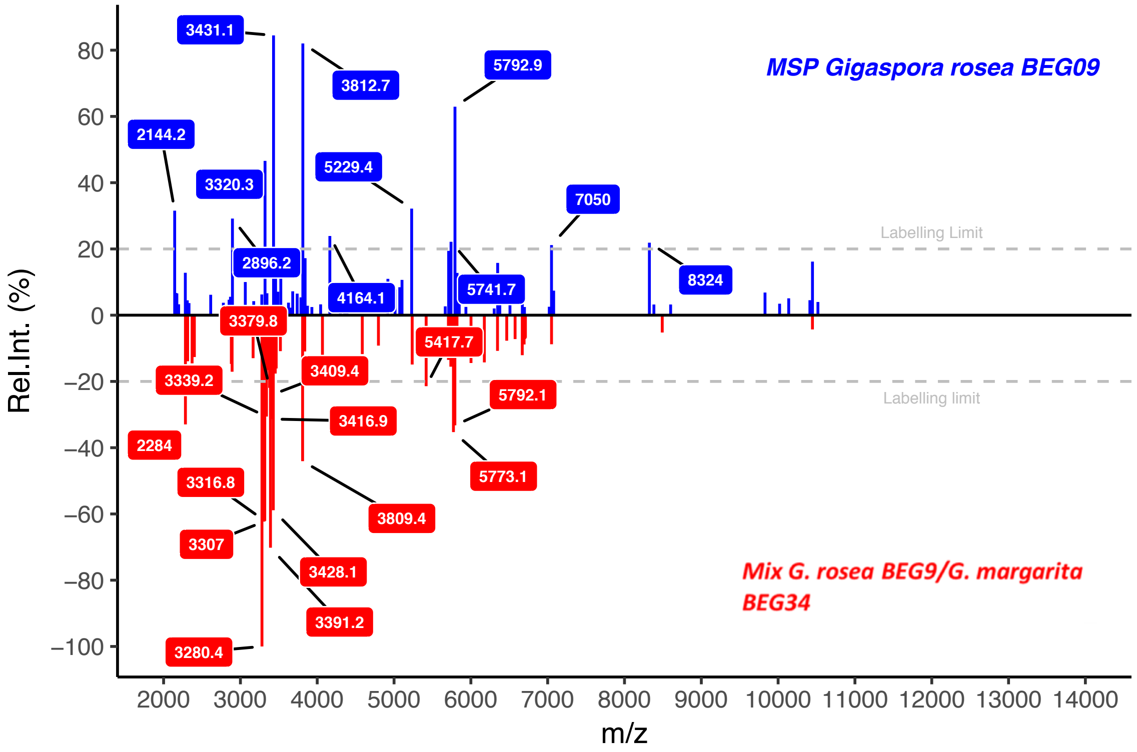

MSP Gigaspora rosea BEG09
Mix G. rosea BEG9/G. margarita BEG34
Labelling Limit
Labelling limit
Rel.Int. (%)
m/z
3431.1
3812.7
5792.9
2144.2
5229.4
3320.3
7050
2896.2
8324
5741.7
4164.1
3379.8
5417.7
3409.4
3339.2
5792.1
3416.9
2284
5773.1
3316.8
3809.4
3307
3428.1
3391.2
3280.4

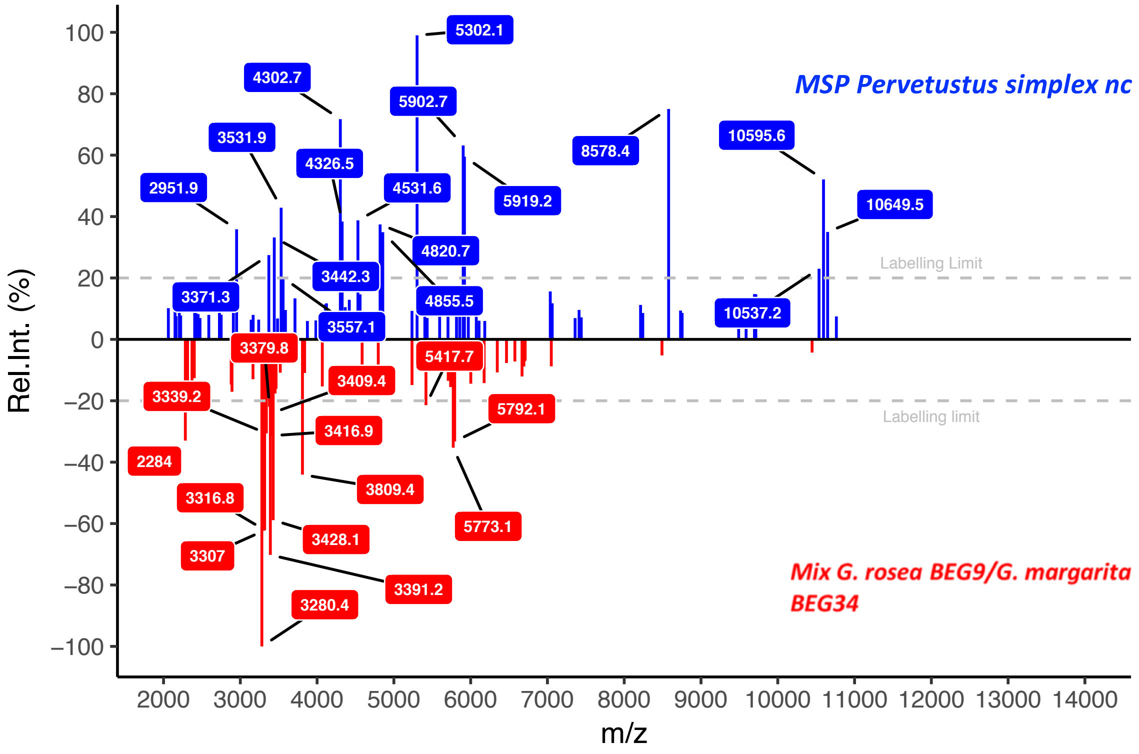
MSP Pervetustus simplex nc
Mix G. rosea BEG9/G. margarita BEG34
Rel.Int. (%)
m/z
Labelling Limit
Labelling limit
5302.1
4302.7
5902.7
3531.9
4326.5
8578.4
10595.6
2951.9
4531.6
5919.2
10649.5
4820.7
3442.3
3371.3
4855.5
10537.2
3557.1
3379.8
5417.7
3339.2
3409.4
5792.1
3416.9
2284
3809.4
3316.8
5773.1
3428.1
3307
3391.2
3280.4

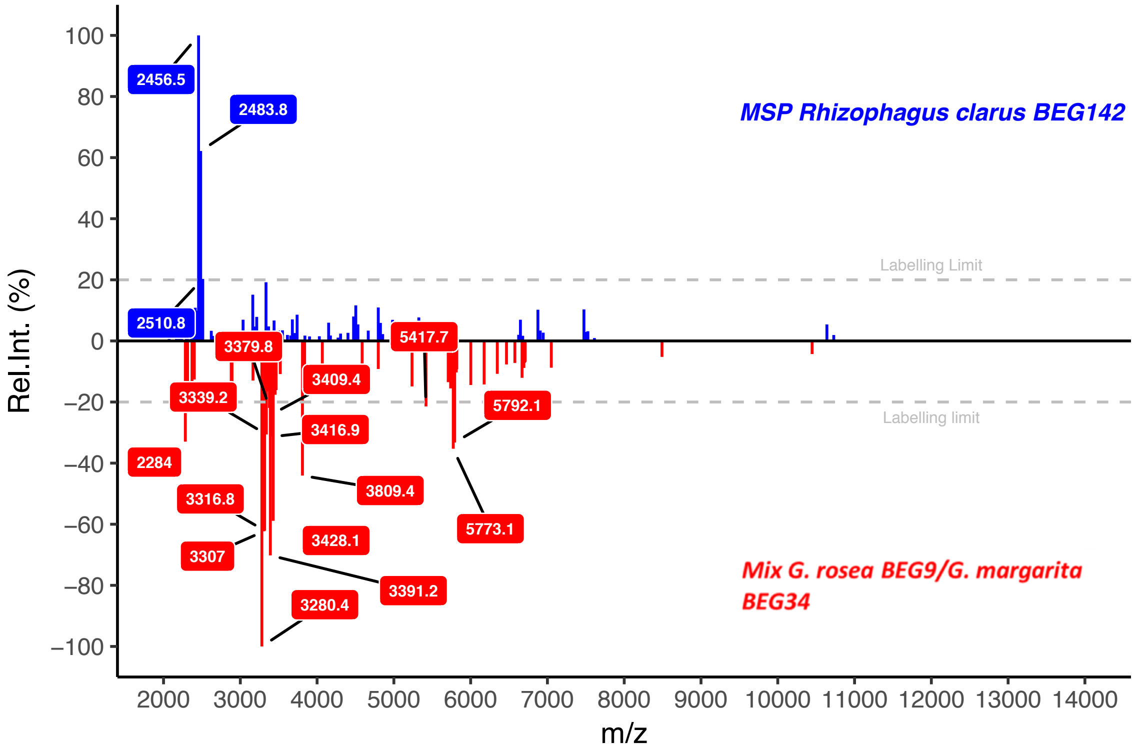
MSP Rhizophagus clarus BEG142
Mix G. rosea BEG9/G. margarita BEG34
Rel.Int. (%)
m/z
Labelling Limit
Labelling limit
2456.5
2483.8
2510.8
3379.8
5417.7
3339.2
3409.4
5792.1
3416.9
2284
3809.4
3316.8
5773.1
3428.1
3307
3391.2
3280.4

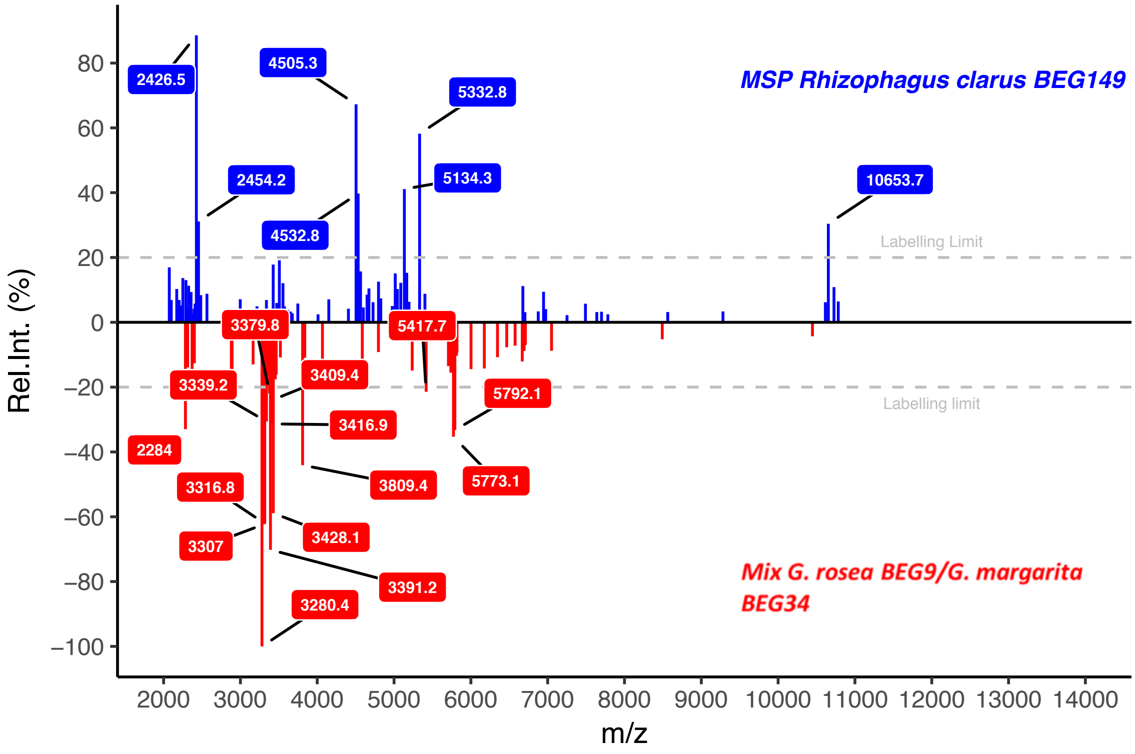
MSP Rhizophagus clarus BEG149
Mix G. rosea BEG9/G. margarita BEG34
Rel.Int. (%)
m/z
Labelling Limit
Labelling limit
2426.5
4505.3
5332.8
2454.2
5134.3
10653.7
4532.8
3379.8
5417.7
3409.4
3339.2
5792.1
3416.9
2284
3809.4
5773.1
3316.8
3428.1
3307
3391.2
3280.4
80
60
40
20
0
−20
−40
−60
−80
−100
2000 3000 4000 5000 6000 7000 8000 9000 10000 11000 12000 13000 14000

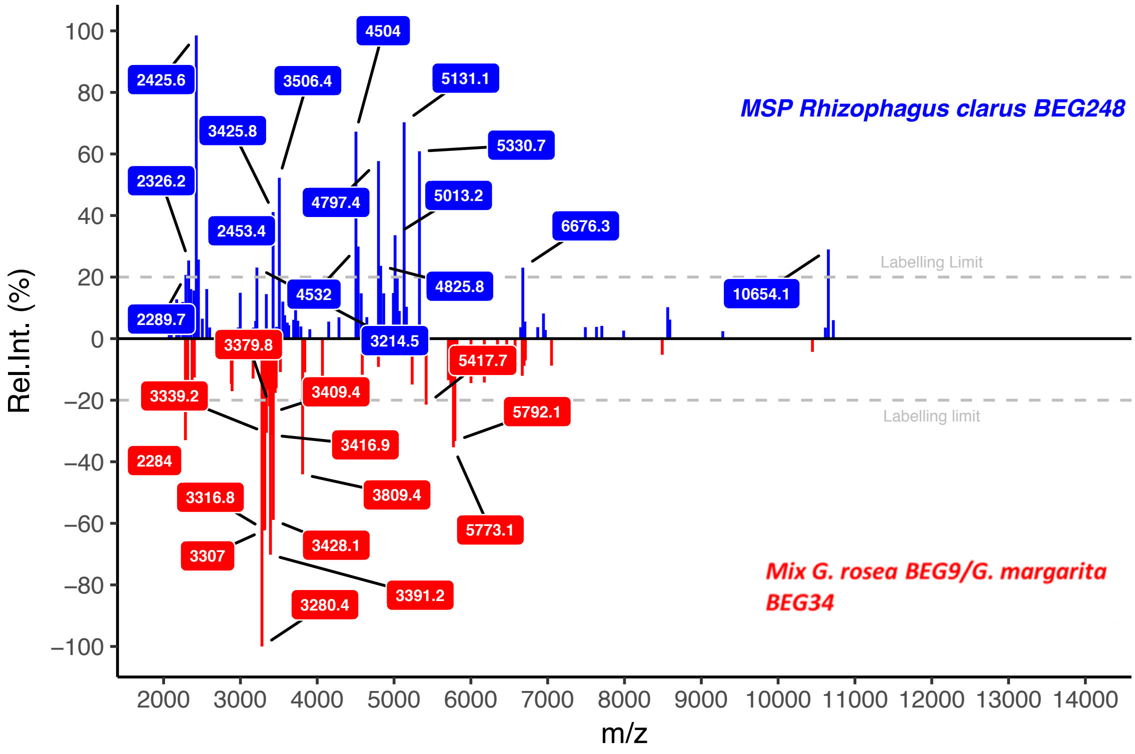
MSP Rhizophagus clarus BEG248
Mix G. rosea BEG9/G. margarita BEG34
Rel.Int. (%)
m/z
Labelling Limit
Labelling limit
4504
2425.6
3506.4
5131.1
3425.8
5330.7
2326.2
4797.4
5013.2
6676.3
2453.4
4532
4825.8
10654.1
2289.7
3379.8
3214.5
5417.7
3339.2
3409.4
5792.1
3416.9
2284
3809.4
3316.8
5773.1
3428.1
3307
3391.2
3280.4
100
80
60
40
20
0
−20
−40
−60
−80
−100
2000 3000 4000 5000 6000 7000 8000 9000 10000 11000 12000 13000 14000

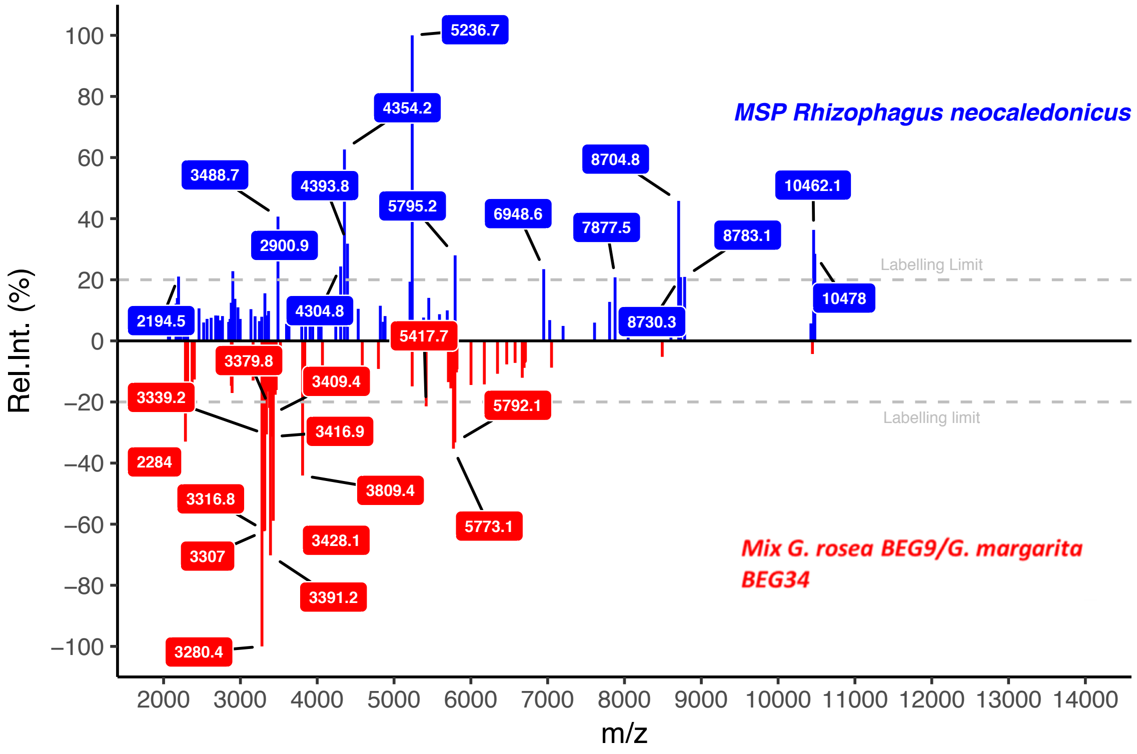
MSP Rhizophagus neocaledonicus
Mix G. rosea BEG9/G. margarita BEG34
Labelling Limit
Labelling limit
Rel.Int. (%)
m/z
5236.7
4354.2
3488.7
4393.8
5795.2
6948.6
8704.8
7877.5
10462.1
8783.1
2900.9
2194.5
4304.8
8730.3
10478
5417.7
3379.8
3339.2
3409.4
3416.9
5792.1
2284
3809.4
3316.8
5773.1
3428.1
3307
3391.2
3280.4

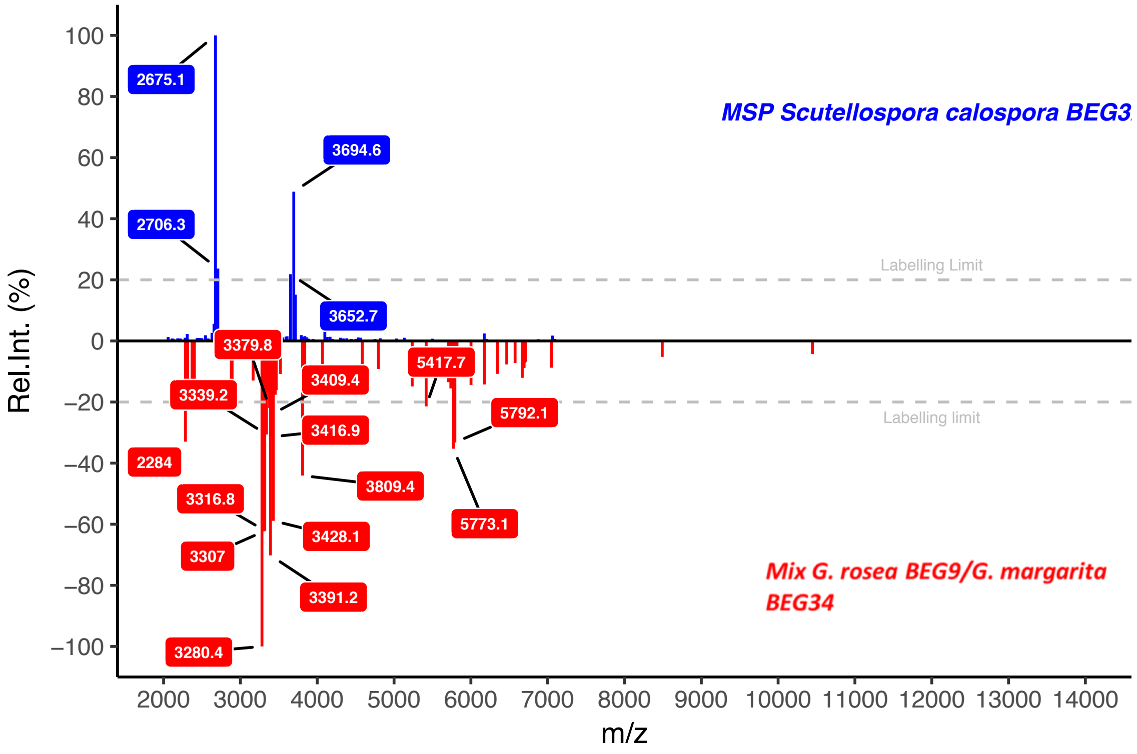
MSP Scutellospora calospora BEG3
Mix G. rosea BEG9/G. margarita BEG34
Labelling Limit
Labelling limit
Rel.Int. (%)
m/z
2675.1
3694.6
2706.3
3652.7
3379.8
5417.7
3339.2
3409.4
3416.9
5792.1
2284
3809.4
3316.8
5773.1
3428.1
3307
3391.2
3280.4

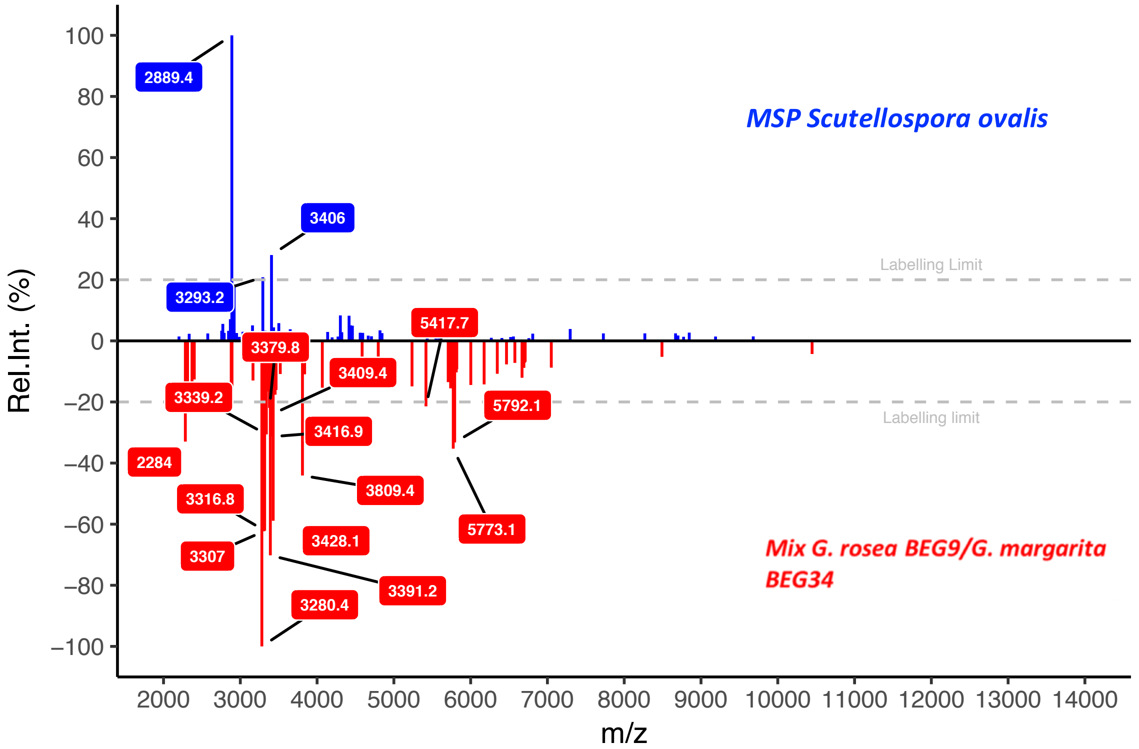

Supplementary Figure 4. Example of using the R (version 3.3.2[30]) MSP importation data base on a mixed-species sample. The "unknown sample" (red; 1 spore of *G. margarita* BEG34/ 1 spore of *G. rosea* BEG9) is matched to MSP reference spectra (blue).
